# Functional diversity of GPCR–Gustducin complexes controls signaling output and suppresses alternative pathways

**DOI:** 10.1038/s41467-026-79528-z

**Published:** 2026-11-14

**Authors:** Victoria Jaime Arce, Mira Lettmann, Mona Stefanie Tögl, Santiago Javier Bonn Garcia, Max Bauer, Maike Neukirch, Olivia Kleinstück, Anastasia Langolf, Isabell Kaczmarek, Daniel Hilger, Hannes Schihada

**Affiliations:** 1 Department of Pharmacy, Institute of Pharmaceutical Chemistry, Marburg University, Marburg, Germany; 2 Rudolf Schönheimer Institute of Biochemistry, Medical Faculty, Leipzig University, Leipzig, Germany

**Keywords:** Cell signalling, Sensors and probes, G protein-coupled receptors

## Abstract

Gustducin (G_gust_), the G protein mediating taste receptor signaling, is expressed not only in sensory taste cells but throughout the human body alongside numerous non-taste G protein-coupled receptors (GPCRs). Whether and how these receptors engage G_gust_, and the functional consequences of such interactions, remain poorly understood. Here, we develop two complementary biosensors that enable direct monitoring of GPCR–G_gust_ interaction and downstream signaling in living cells, allowing pharmacological characterization of G_gust_ activity induced by non-taste GPCRs. We find that several non-taste GPCRs do not activate G_gust_ but instead stabilize the G protein heterotrimer and suppress basal signaling by forming unproductive GPCR–G protein complexes, whereas others induce conventional G_gust_ activation and signal transduction. We further show that these unproductive GPCR–G_gust_ complexes suppress the activation of alternative signaling pathways by sequestering receptors from other G proteins. This mode of interaction functionally partitions GPCRs into activators and inactivators of G_gust_ and represents a mechanism for balancing concurrent signaling pathways within a cell.

## Abstract (web summary)

Gustducin mediates taste signaling but is also found throughout the body. Here, the authors show that non-taste GPCRs can activate or suppress gustducin signaling, revealing a mechanism that balances concurrent signaling pathways within cells.

## Introduction

Gα_gustducin_ (Gα_gust_), encoded by *GNAT3*, is a member of the G_i/o_ family of G proteins. Gα_gust_ is the major component of the G protein heterotrimer G_gust_ (Gα_gust_βγ), which is composed of Gα_gust_ and a Gβγ dimer, and plays a central role in taste perception by coupling bitter (TAS2Rs) and sweet/umami (TAS1Rs) tastant-binding G protein-coupled receptors (GPCRs) to intracellular signaling pathways^1–3^. Gα_gust_ is closely related to transducins Gα_t1_ and Gα_t2_ (encoded by *GNAT1* and *GNAT2*, respectively), which mediate vision in rod and cone photoreceptor cells. Transducins and gustducin, as well as other G_i/o_-family Gα subunits and Gα_s_, can enhance phosphodiesterase (PDE) activity primarily through binding to the inhibitory γ-subunit of PDEs^4–7^. This interaction relieves autoinhibition of the catalytic subunits, resulting in increased PDE activity and, consequently, reduced intracellular levels of the cyclic nucleotides cAMP and cGMP. In addition, Gβγ released from activated Gα_gust_ activates phospholipase Cβ2 (PLCβ2), promoting IP_3_- and Ca^2+^-dependent signaling and opening of transient receptor potential cation channels subfamily M member 5 (TRPM5) channels, which leads to depolarization and neurotransmitter release^8–10^. In addition to its role in type II taste receptor cells of the tongue and palate, Gα_gust_ is expressed in specialized epithelial cells throughout the stomach, small intestine, and colon. Here, Gα_gust_ links taste receptors to glucagon-like peptide 1 (GLP-1) secretion, ghrelin release, and expression of sodium-dependent glucose transporter isoform 1 (SGLT1)^11–13^.

The emergence of single-cell and single-nucleus sequencing techniques enabled organism-wide protein expression profiling, revealing that Gα_gust_ can also be co-expressed with non-taste receptors in the human body. Beyond the tongue, the human protein atlas reports Gα_gust_ expression in the brain, testis and adipose tissue, and – at high levels – in the duodenum and other parts of the small intestine^14,15^. These tissues also express numerous non-taste GPCRs. However, whether G_gust_ can couple to and mediate signaling of these receptors remains unclear.

In the present study, we address this knowledge gap using an integrative approach that combines the analysis of single-cell expression data with herein developed biosensors for pathway-unbiased G_gust_ activation and Gα_gust_-mediated signal transduction. Using these tools, we find that several non-taste GPCRs can couple to Gα_gust_-containing heterotrimers. Interestingly, this coupling results in two functionally distinct complexes: conventional signaling complexes that promote G_gust_ activation, such as those formed with the A_1_ adenosine receptor (A_1_R) and histamine H_3_ receptor (H_3_R), and complexes that suppress basal Gα_gust_ signal transduction, such as those formed with the histamine H_2_ receptor (H_2_R) or the A_2A_ adenosine receptor (A_2A_R). Nucleotide-sensitivity experiments confirm these GPCR–G_gust_ interactions. These experiments suggest a mechanism by which G_gust_ may modulate gastrointestinal physiological functions in response to non-taste receptor ligands.

## Results

### A biosensor for G_gust_ heterotrimer dissociation in cells

**Fig. 1:**
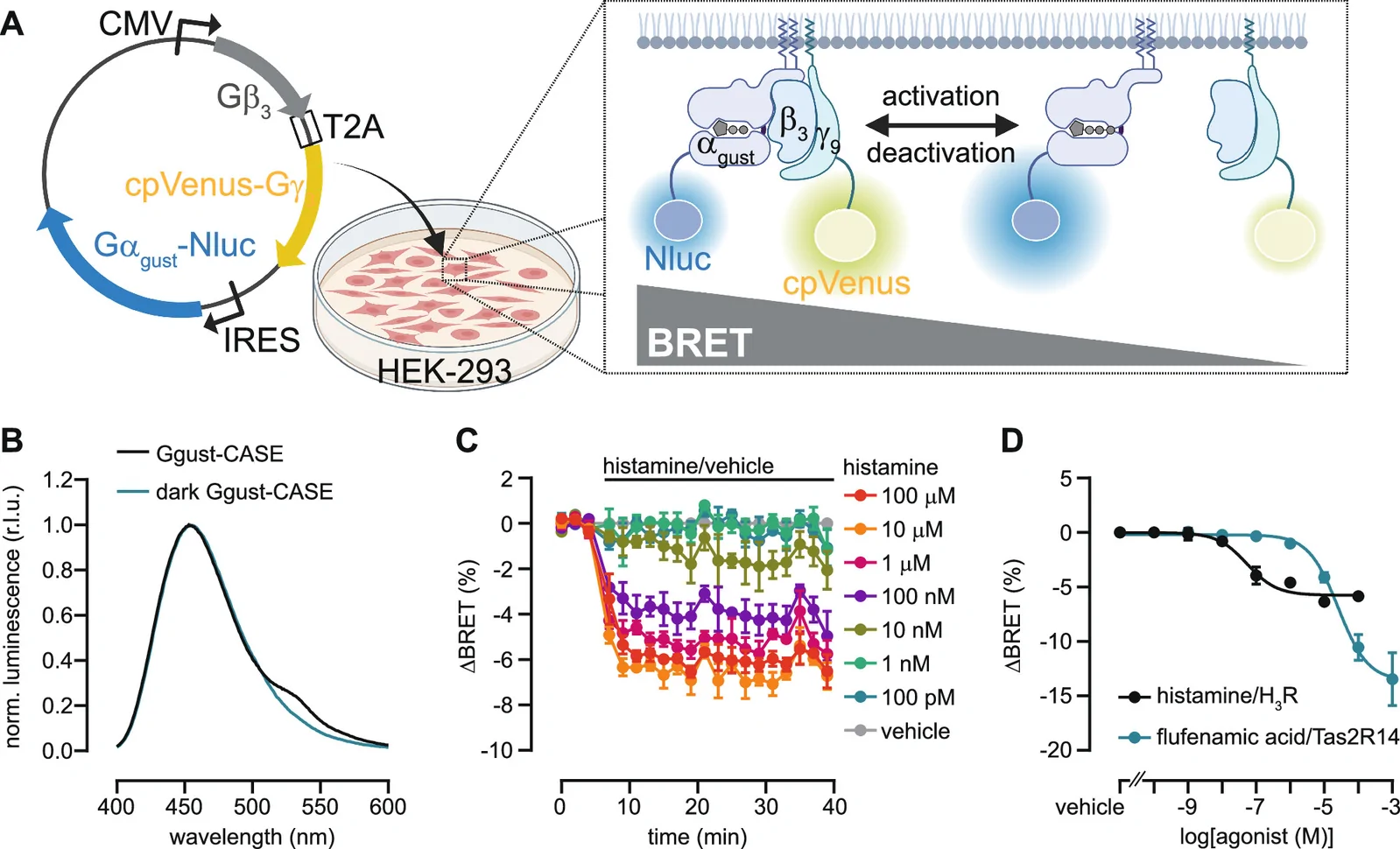
Development of the G_gust_ dissociation biosensor, Ggust-CASE. **A** Scheme of the plasmid design and biosensor principle. Created in BioRender. Schihada, H. (2026) https://BioRender.com/ygab41y. **B** Peak-normalized luminescence emission spectra of Ggust-CASE and dark Ggust-CASE harboring the Y218W point mutation in cpVenus. **C** ΔBRET time course of Ggust-CASE upon stimulation of co-expressed H_3_R with histamine. **D** ΔBRET concentration-response curves of histamine and flufenamic acid measured with Ggust-CASE in H_3_R- and Tas2R14-expressing cells, respectively. Data in (**B**) show one representative experiment. Data in (**C**) and (**D**) represent the mean ± SEM of three biological replicates. Experiments were conducted in HEK-293A cells that were transiently transfected with either Ggust-CASE (**B**–**D**) or dark Ggust-CASE (**B**), either without (**B**) or with H_3_R (**C**, **D**) or Tas2R14 (**D**). Source data are provided as a Source Data file. CMV: Cytomegalovirus promoter; T2A: Thosea asigna virus 2A peptide; IRES: Internal Ribosome Entry Site; HEK-293: human embryonic kidney-293 cells.

Given the divergent signaling cascades promoted upon G_gust_ dissociation into Gα_gust_ and Gβγ, we aimed to develop a signaling-pathway independent readout of G_gust_ activation. In line with the placement of energy partners in a TRUPATH G_gust_ heterotrimer dissociation sensor^16^, we fused NanoLuciferase^17^ (Nluc), flanked by flexible linkers, to Asp116 in Gα_gust_. Next, we combined the sequence of Gα_gust_-Nluc with the wild-type Gβ_3_ gene and N-terminally labeled Gγ_9_ on a plasmid backbone comprising a T2A self-cleavage site (between Gβ_3_ and cpVenus-Gγ_9_) and an internal ribosome entry site (IRES; upstream of Gα_gust_-Nluc), because this Gβγ dimer fused with the optimized energy acceptor cpVenus^18,19^ proved to be a sensitive module to detect the dissociation of other G protein heterotrimers^20,21^. This plasmid design enabled the simultaneous co-transfection and expression of all three G protein subunits (Fig. 1A, Supplementary Fig. 1A). To facilitate the future combination of this sensor, which we refer to as Ggust-CASE, with other optical biosensors, we created a version of G-CASE with a dimmed BRET acceptor (“dark Ggust-CASE”) by mutating Tyr218 in cpVenus to Trp^22^. The quenched emission of cpVenus was confirmed in the luminescence emission spectra of transfected cells (Fig. 1B). Starting from dark Ggust-CASE, we also generated a Ggust-CASE variant with quenched emission of Nluc by mutating Arg164 to Gln in the luciferase. This construct, called “double dark Ggust-CASE”, exhibited only 0.2 % of the luminescence emission compared to dark Ggust-CASE (comparison of the area under the curves from 400 – 600 nm; Supplementary Fig. 2). Next, we tested whether Ggust-CASE responds to agonist-mediated activation of a G_gust_-coupled taste receptor and activation of a prototypical G_i/o_-coupled receptor. Stimulation of the co-expressed receptor, either the taste receptor Tas2R14 with flufenamic acid, or the histamine H_3_ receptor (H_3_R) with histamine, resulted in a concentration-dependent decrease in BRET of Ggust-CASE (Fig. 1C, D). The potencies of flufenamic acid (mean ± SEM pEC_50_ = 4.62 ± 0.15) and of histamine (mean ± SEM pEC_50_ = 7.24 ± 0.23) were consistent with flufenamic acid-induced Tas2R14-miniGα_gust_ coupling reported earlier^23^ and with the activation of other G_i/o_ biosensors by histamine/H_3_R^20^, respectively.

### A biosensor for Gα_gust_-mediated signal transduction in cells

**Fig. 2:**
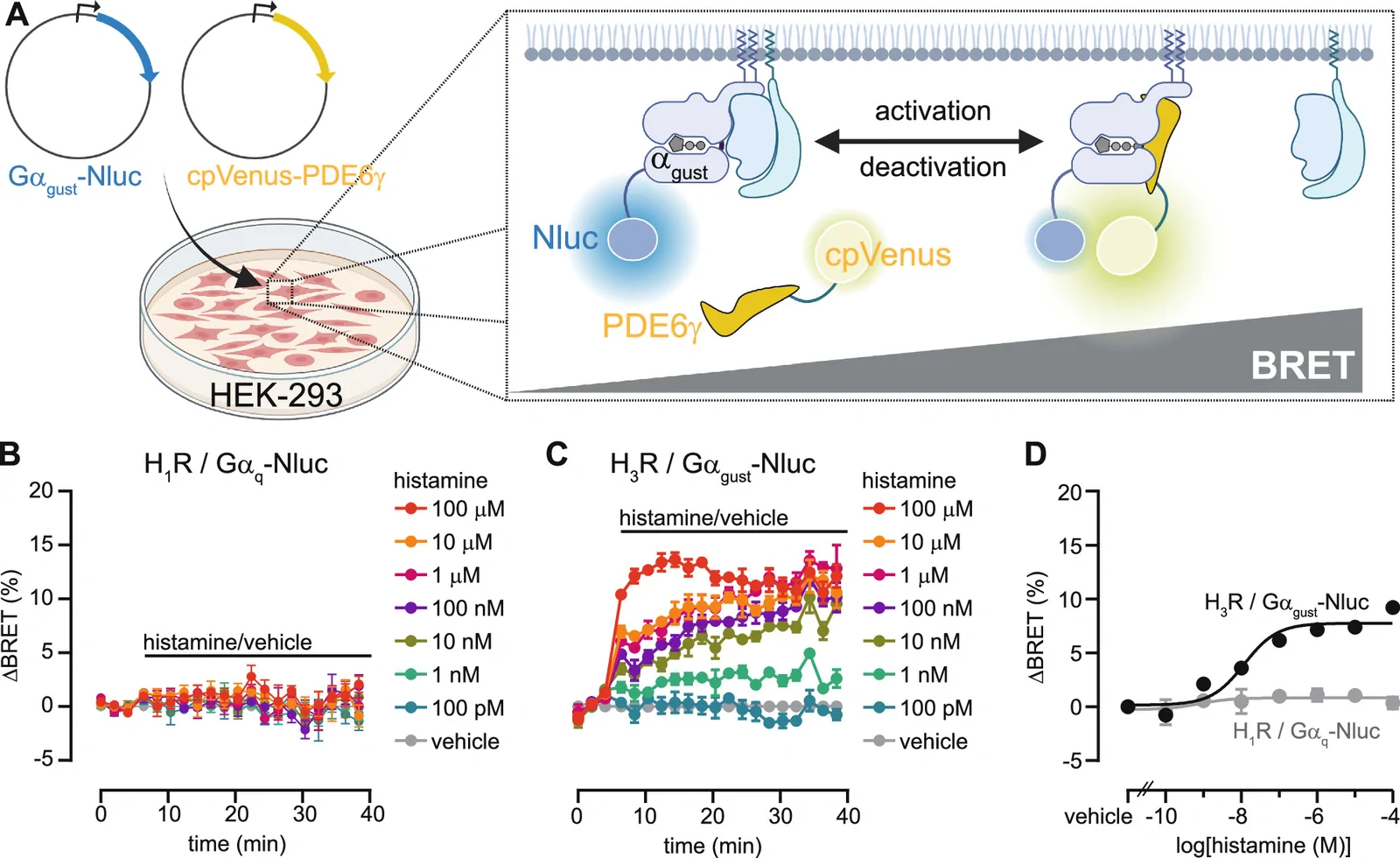
Development of a G_gust_–PDE6 interaction assay. **A** Scheme of the BRET-based protein–protein interaction assay. Created in BioRender. Schihada, H. (2026) https://BioRender.com/y418bw7. Time courses (**B**, **C**) and concentration-dependent BRET changes (**D**) induced by histamine in HEK-293A cells that were transiently transfected with PDE6γ-cpVenus, along with either H_1_R and Gα_q_-Nluc (**B**, **D**), or H_3_R and Gα_gust_-Nluc (**C**, **D**). Data represent the mean ± SEM of three biological replicates. Source data are provided as a Source Data file. PDE6γ: γ-subunit of phosphodiesterase 6; HEK-293; human embryonic kidney-293 cells.

To expand our G_gust_-targeted toolbox to include a cell-based readout of Gα_gust_-mediated signal transduction, we tested whether known effectors of the different Gα subtypes would interact with Gα_gust_ following heterotrimer dissociation. We used an intermolecular BRET setup to combine cpVenus- or YFP-labeled Gα effectors with Gα-Nluc. Our analysis included the Gα_q/11_ effectors PLCβ3, p63RhoGEF and GRK2^24–27^, the Gα_12/13_ effector PDZ-RhoGEF^24^, the Gα_i/o_(GTP) and Gα_s_(GTP) detectors KB1753 and KB1691^28^, and the Gα_i/o_- and Gα_s_-effector adenylyl cyclase 5 (AC5)^29^, labeled either N-terminally at the C1 domain (coupling site for Gα_i/o_) or C-terminally at the C2 domain (coupling site for Gα_s_), respectively (Supplementary Fig. 3A). We detected GPCR agonist-induced increases in BRET for all effectors with their cognate Gα-Nluc partners. However, Gα_gust_-Nluc only exhibited a very modest, histamine concentration-dependent increase in BRET with C1 domain-labeled AC5 and an about 10 % response with the cpVenus-tagged Gα_i/o_(GTP) detector KB1753 (Supplementary Fig. 3B–I). Alternatively, we also assessed whether activation of G_gust_, similar to other members of the G_i/o_ family, decreases intracellular cAMP levels as a consequence of adenylate cyclase inhibition. However, cAMP measurements in HEK-293A cells expressing Gα_gust_ as the sole Gα subtype^30^ showed that G_gust_ activation by Tas2R14 and H_3_R does not alter intracellular cAMP levels (Supplementary Fig. 3J). Due to the low signal-to-noise ratio obtained with Gα_gust_-Nluc/YFP-AC5 and the relatively low potency of histamine in the Gα_gust_-Nluc/KB1753-cpVenus protein–protein interaction assay (Gα_i2_-Nluc/KB1753-cpVenus mean ± SEM pEC_50_ = 6.93 ± 0.08 vs. Gα_gust_-Nluc/KB1753-cpVenus mean ± SEM pEC_50_ = 5.17 ± 0.19), we searched the literature for other potential interaction partners of active Gα_gust_. Co-immunoprecipitation experiments revealed that, similar to α- and β-transducin, Gα_gust_(GTP) physically interacts with the γ-subunit of PDE6^5–7^. We therefore co-expressed Gα_gust_-Nluc or Gα_q_-Nluc, the latter serving as a negative control, with cpVenus-labeled PDE6γ and H_3_R or the G_q_-coupled H_1_R, respectively (Fig. 2A). Here, histamine induced a concentration-dependent increase in BRET of approximately 10 %, with a more than 500-fold higher potency than observed for the Gα_gust_-Nluc/KB1753-cpVenus readout (mean ± SEM pEC_50_ = 7.92 ± 0.07). Other members of the Gα_i/o_ protein family (Gα_i2_ and Gα_i3_; Supplementary Fig. 4) also showed a significant BRET increase upon H_3_R stimulation with histamine, while co-expression of H_1_R/Gα_q_-Nluc, which served as a negative control, did not induce significant changes in BRET. These results are indicative of an enhanced interaction between Gα_gust_ and PDE6γ, and Gα_gust_-mediated signal transduction upon G protein activation (Fig. 2B–D). Histamine potency was approximately five-fold higher in the Gα_gust_–PDE6γ interaction assay than in the Ggust-CASE readout, consistent with signal amplification along the signaling cascade. We also titrated the plasmid ratios of co-transfected Gα_gust_-Nluc and cpVenus-labeled PDE6γ to identify the optimal conditions for this intermolecular BRET assay but found no significant differences between the test conditions (Supplementary Fig. 5).

### GPCR-specific conformational responses in the G_gust_ heterotrimer

**Fig. 3:**
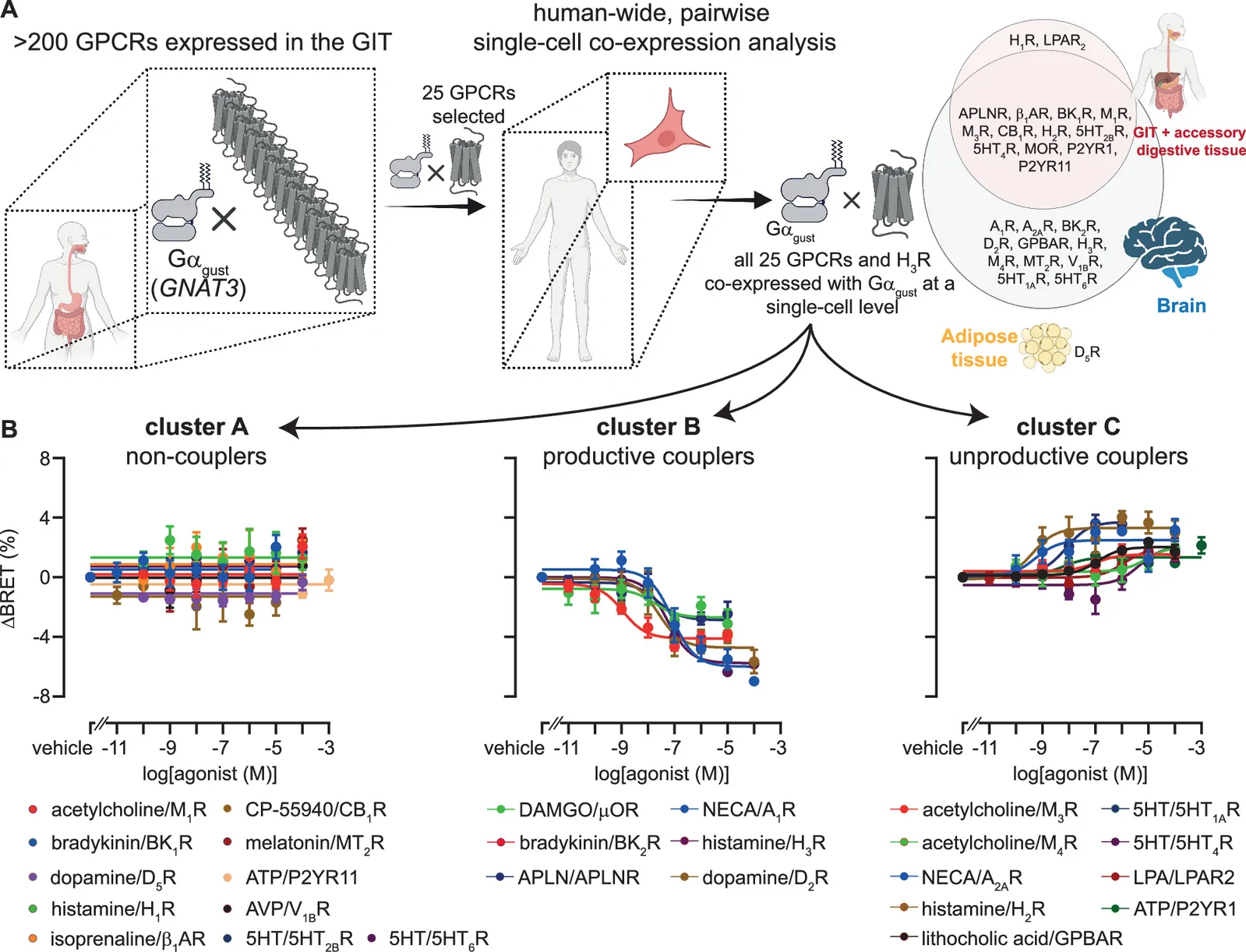
Profiling Ggust-CASE responses to gastrointestinal GPCR activation. **A** Workflow of the co-expression analysis of GPCRs with Gα_gust_ at tissue- and single-cell levels. Created in BioRender. Schihada, H. (2026) https://BioRender.com/tsiuw84. **B** Concentration-dependent BRET changes induced by non-taste GPCRs transiently expressed in HEK-293A cells alongside Ggust-CASE and stimulated with agonists. GPCRs are clustered into three groups, group A (non-couplers), group B (productive couplers), and group C (unproductive couplers), based on the BRET responses induced. Data represent the mean ± SEM of three biological replicates. Non-G_gust_-couplers (group A) were identified by comparing a linear model with a zero slope to a sigmoidal concentration–response model (with the Hill slope fixed to +1 or −1) using a two-sided extra sum-of-squares F-test (*p* > 0.05). Source data are provided as a Source Data file. GIT: gastrointestinal tract.

The primary physiological function of G_gust_ is linked to the sensation of taste, but its expression in epithelial cells that line the stomach, small intestine, and colon^14^ suggests that it could also act as a transducer of other extracellular signals when coupled to a non-gustatory GPCR. We therefore examined expression datasets from the Human Protein Atlas^14^ to identify GPCRs that may be coupled to G_gust_ in the small intestine and duodenum. In total, 202 GPCRs are co-expressed with Gα_gust_ (*GNAT3*) in the duodenum and 201 GPCRs are expressed in the small intestine (Supplementary Data 1). From this set of receptors, we selected 25 pharmacologically accessible GPCRs with known agonists and G protein coupling information, according to guidetopharmacology.org and gproteindb.org^31,32^. Our selection included GPCRs whose collective coupling profiles covered all four major Gα families^33^. Next, we performed an organism-wide co-expression analysis of these receptors with *GNAT3* at the single-cell level (Supplementary Data 2). Our analysis also included the H_3_R, which we had used as a model receptor for sensor validation before. Our investigation of publicly available single-cell RNA sequencing data^15^ confirmed that all 26 GPCRs are co-expressed with *GNAT3* at a single-cell level in humans. However, only 14 of these are co-expressed with *GNAT3* in gastrointestinal tract (GIT) cells or cells of accessory digestive tissue (ADT) (e.g., the salivary gland). The majority of GPCRs included in our analysis (23 out of 26) were co-expressed with *GNAT3* in brain cells; a subset of these receptors also exhibited co-expression with *GNAT3* in non-GIT/non-ADT cells outside the brain (Fig. 3A). For most GPCRs, the degree of co-expression with *GNAT3* was in the low single-digit percentage range of sequenced cells belonging to the same subtype. Maximum expression overlap with *GNAT3* was detected for *CHRM3* and *P2YR1* in stomach tuft cells (17%), while the minimum degree of co-expression was observed for *AVPR1B*, *BDKRB2* and *HTR1A* (0.02 – 0.03% in pancreatic islet cells, ependymal glial cells, and brain excitatory neurons, respectively (Supplementary Data 2). We confirmed that all 26 receptors were able to engage sensors of the primary and/or secondary G proteins upon co-expression and stimulation with agonist (Supplementary Fig. 6). Then, we subjected all 26 receptors to our cell-based pharmacological testing system, expressing them alongside Ggust-CASE in HEK-293A cells in order to measure the biosensor’s response to GPCR stimulation by agonists (Fig. 3B). Of the 26 receptors tested, 11 did not mediate BRET changes in Ggust-CASE and are therefore considered as non-G_gust_ couplers. In contrast, 13 GPCRs mediated agonist concentration-dependent BRET changes in Ggust-CASE. Surprisingly, GPCR activation induced not only decreases in Ggust-CASE BRET (e.g., for the adenosine A_1_ receptor, A_1_R), which were sensitive to pre-incubation with GPCR antagonists (Supplementary Fig. 7), but also positive BRET changes were observed. The latter are similar to earlier studies with other G protein biosensors^34–36^. The increase in BRET was most prominent for the histamine H_2_ receptor (H_2_R), a primary G_s_-coupled GPCR and important regulator of gastric acid secretion in the stomach^37^ – where the H_2_R is co-expressed with Gα_gust_. We profiled the H_2_R-mediated G-CASE responses of other G protein subtypes (Supplementary Fig. 8), including other members of the G_i/o_ protein family and previously developed and validated biosensors for G_11_ and G_o2_ heterotrimers (G11-CASE and Go2-CASE; Supplementary Fig. 9, Supplementary Fig. 10), to confirm that the positive BRET change was specific to G_gust_. Additionally, we validated that the responses of Ggust-CASE obtained with overexpressed adenosine, histamine, and serotonin receptors were not influenced by endogenous GPCRs expressed in HEK-293A cells (Supplementary Fig. 11). Of note, all receptors that induced a decrease in Ggust-CASE BRET have previously been shown to activate G_i/o_ family G proteins, including BK_2_R, which exhibits promiscuous G protein coupling^33^.

**Fig. 4:**
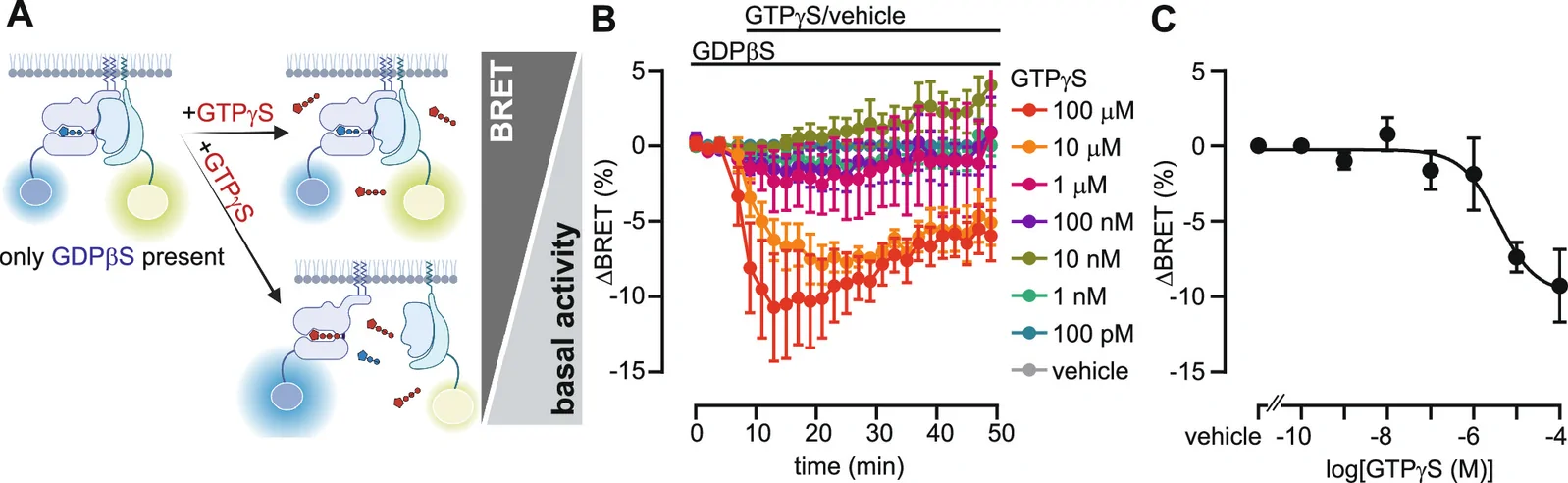
Confirmation of basal G_gust_ activity. **A** Scheme showing the principle of the assay to determine the basal activity of G_gust_ in permeabilized and endogenous nucleotide-depleted cells. Created in BioRender. Schihada, H. (2026) https://BioRender.com/4lk8udp. **B**, **C** Concentration-dependent BRET changes induced by GTPγS in HEK-293A cells that have been transiently transfected with Ggust-CASE. Data represent the mean ± SEM of three biological replicates. Source data are provided as a Source Data file. GDPβS: guanosine 5′-[β-thio]diphosphate; GTPγS: guanosine 5′-[γ-thio]triphosphate.

The receptor-dependent BRET responses of Ggust-CASE with opposing directions suggest distinct GPCR-mediated changes in conformation of the G_gust_ heterotrimer. While a decrease in FRET or BRET between Gα- and Gβγ-fused energy partners is widely accepted as representing heterotrimer dissociation and activation, an increase in FRET/BRET, as observed with the H_2_R, could be explained by receptor-mediated reduction of basal G_gust_ activity through the formation of unproductive GPCR–G protein complexes as previously observed with the vasopressin V_2_ receptor (V_2_R) and G_12_ heterotrimer biosensors^38^. To test the hypothesis that Gα_gust_ exhibits distinct responses to different GPCRs, we used an established intermolecular BRET assay based on masGRK3ct-Nluc and Venus-Gβγ to monitor Gβγ release from Gα_gust_ in cells lacking other Gα subtypes^30,39,40^. This assay confirmed that supposedly unproductive G_gust_ couplers, such as H_2_R and A_2A_R, do not induce Gβγ release from G_gust_ heterotrimers (Supplementary Fig. 12). Next, we aimed to investigate whether G_gust_ exhibits basal activity in HEK-293A cells. We expressed Ggust-CASE without additional GPCRs and depleted the endogenous guanosine nucleotides GDP and GTP using apyrase following digitonin-mediated cell permeabilization^41^. After loading the G protein sensors with the apyrase-resistant GDP analog GDPβS and recording the BRET baseline of our biosensor, we added a tenfold excess of GTPγS to restore the GDP/GTP ratio present in intact cells (Fig. 4A). GTPγS induced a concentration-dependent decrease in Ggust-CASE BRET (Fig. 4B, C). The apparently low potency of GTPγS observed in our experiments with Ggust-CASE (EC_50_ [95 % CI] = 3.7 μM [0.8 – 15.1 μM]) compared to biochemical analyses of purified Gα proteins (e.g., k_*D*_ of GTPγS at purified Gα_t1_ 50 – 100 nM^42^) could also be observed with other G-CASE biosensors (Supplementary Fig. 13) and may be explained by the presence of Gβγ and other modulators of G protein activity, such as regulators of G protein signaling (RGS) proteins and guanine nucleotide dissociation inhibitors (GDIs)^43,44^, in our experimental setup. The decrease in BRET induced by GTPγS indicates that G_gust_ is at least partially active in intact cells prior to the stimulation of GPCRs by an agonist.

**Fig. 5:**
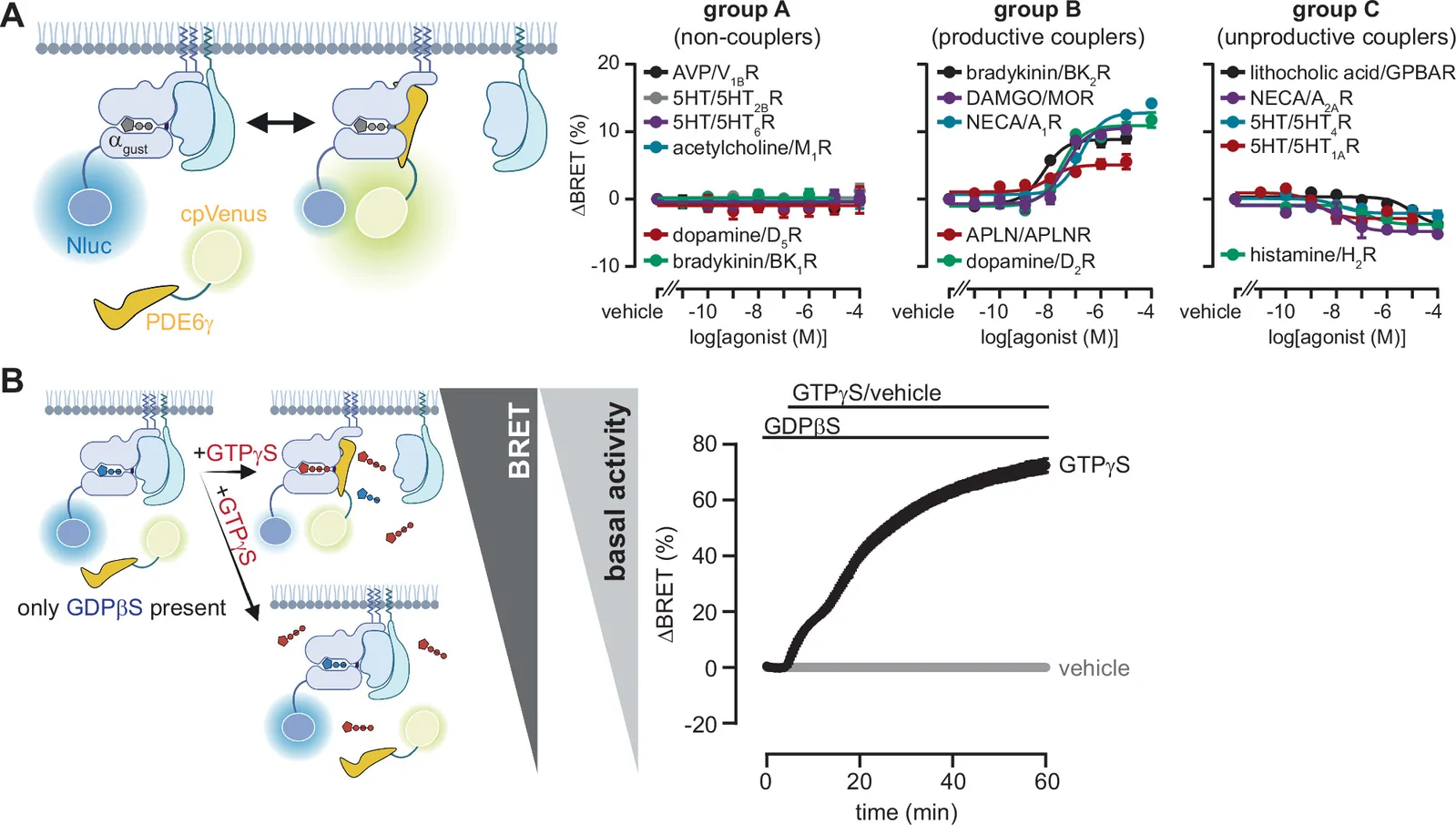
GPCR-mediated G_gust_ activation can enhance or reduce the basal interaction between G_gust_ and PDE6. **A** Scheme of the assay principle and concentration-dependent BRET changes induced by GPCR agonists in HEK-293A cells that have been transiently transfected with the indicated GPCRs, dark Ggust-CASE and PDE6γ-cpVenus. Created in BioRender. Schihada, H. (2026) https://BioRender.com/b314ysa. **B** Scheme of the assay principle and GTPγS-induced BRET changes in HEK-293A cells that have been transiently transfected with dark Ggust-CASE and PDE6γ-cpVenus. Created in BioRender. Schihada, H. (2026) https://BioRender.com/8sl8tch. Data represent the mean ± SEM of three biological replicates. Source data are provided as a Source Data file. PDE6γ: γ-subunit of phosphodiesterase 6; GDPβS: guanosine 5′-[β-thio]diphosphate; GTPγS: guanosine 5′-[γ-thio]triphosphate.

To validate our hypothesis that receptors mediate the suppression of basal G_gust_ activity, we used our Gα_gust_–PDE6γ interaction assay to probe a selected subset of receptors from each of the three groups identified in the G_gust_ dissociation assay – non-G_gust_-couplers (group A; no change in BRET with Ggust-CASE), productive G_gust_-couplers (group B; decrease in BRET of Ggust-CASE) and unproductive G_gust_-couplers (group C; increase in BRET of Ggust-CASE). Experiments were performed using dark Ggust-CASE (carrying the loss-of-fluorescence mutation Y218W in cpVenus and wild-type Nluc on Gα_gust_) and PDE6γ-cpVenus. Interestingly, we found that receptors that induced an increase in Ggust-CASE (group C) reduced the interaction between Gα_gust_ and PDE6γ, and vice versa (Fig. 5A). This correlation suggests (i) basal interaction between Gα_gust_ and PDE6γ already prior to GPCR activation by an agonist and that (ii) the activation of these receptors by their agonists shifts the balance of G_gust_ activity towards inactive states. Using the experimental setup of permeabilized and nucleotide-controlled cells that we established earlier, we confirmed the basal interaction between Gα_gust_ and PDE6γ. The addition of GTPγS to GDPβS-preloaded cells enhanced BRET between these labeled interaction partners (Fig. 5B), suggesting that Gα_gust_ and PDE6γ already interact prior to GPCR-mediated G_gust_ activation in intact cells, where both GDP and GTP are present.

### Productive and unproductive GPCR–G_gust_ complexes revealed by nucleotide sensitivity experiments

**Fig. 6:**
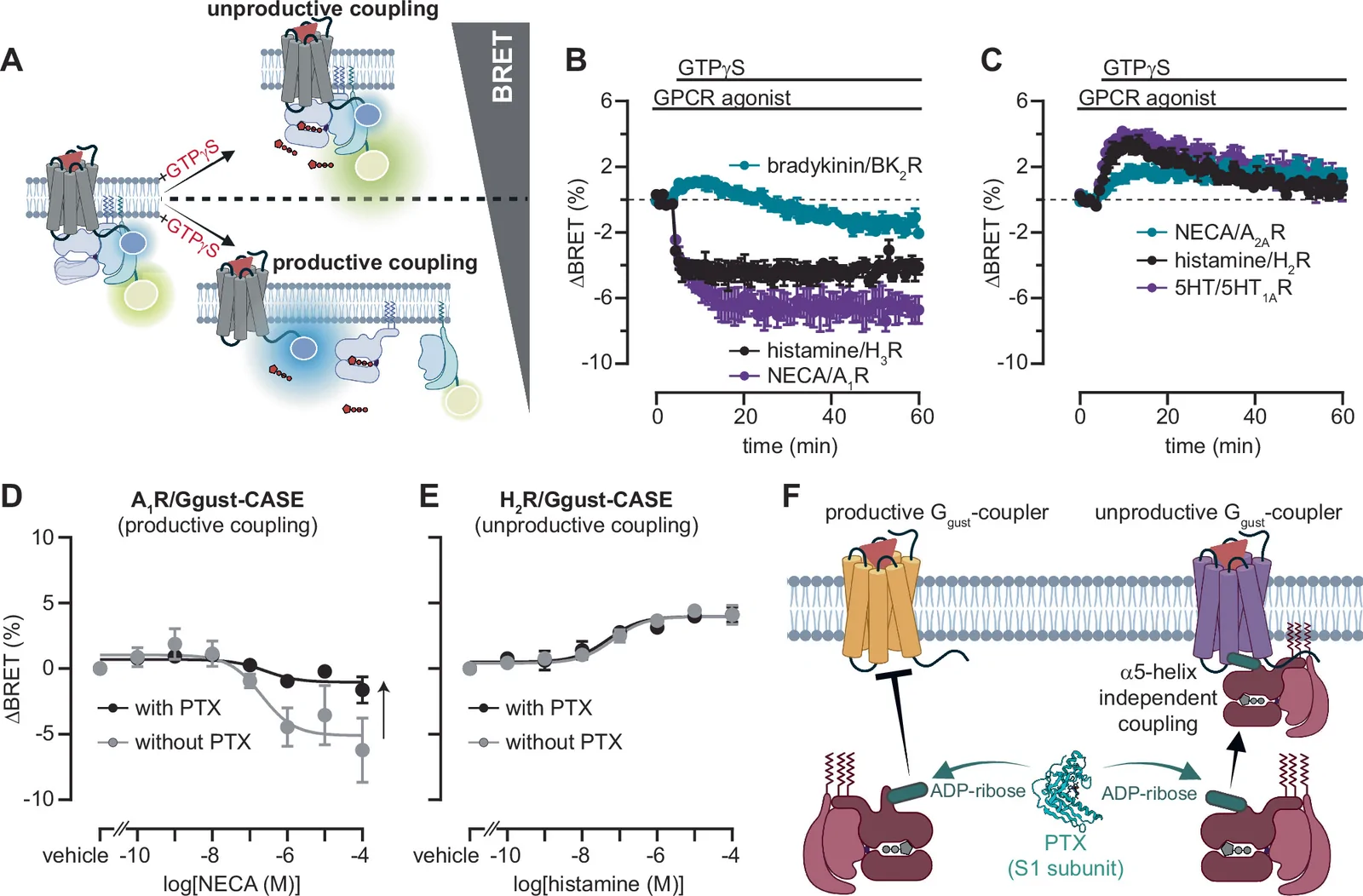
Nucleotide and PTX sensitivity of GPCR–G_gust_ complexes. **A** Scheme of the assay principle. Created in BioRender. Schihada, H. (2026) https://BioRender.com/6079u0p. GTPγS-induced BRET changes between G_gust_ heterotrimers and supposedly productive (**B**) and unproductive (**C**) G_gust_ couplers. **D**, **E** Concentration-dependent BRET changes induced by NECA and histamine agonists in HEK-293A cells that have been transiently transfected with the indicated GPCRs, Ggust-CASE and the S1 subunit of PTX, or vehicle control. **F** Scheme of the Gα_gust_ α5-helix-dependent and -independent coupling modes for productive and unproductive GPCR-G_gust_ complexes, respectively. Created in BioRender. Schihada, H. (2026) https://BioRender.com/8wpvx1r. Experiments in (**B**, **C**) were conducted in endogenous Gα-deficient HEK-293A cells that had been transiently transfected with the indicated GPCR-Nluc constructs, as well as Gα_gust_ and Venus-labeled Gβ_1_γ_2_. Experiments in (**D**, **E**) were conducted in HEK-293A cells that had been transiently transfected with Ggust-CASE, and A_1_R or H_2_R with or without the S1 subunit of pertussis toxin (PTX). Data represent the mean ± SEM of three biological replicates. Statistical significance between the plateau responses in (**D**, **E**) was tested using a two-sided extra-sum-of-squares F-test and is indicated with the black arrow (*p* < 0.05). Source data are provided as a Source Data file. GTPγS: guanosine 5′-[γ-thio]triphosphate. PTX: pertussis toxin.

The enhancement and reduction of the GPCR-specific Gα_gust_–PDE6γ interaction were found to be anti-correlated with Ggust-CASE BRET responses. This suggests that some GPCRs form productive complexes with G_gust_, while others form unproductive complexes, as previously described for some G_13_-coupled GPCRs^38^ and virus-encoded, G_i_-coupled receptors^45^. To test this hypothesis, we adapted an established intermolecular BRET approach, which detects the proximity of a luciferase-labeled GPCR with fluorescent Gβ_1_γ_2_^38,46,47^, to assay the nucleotide sensitivity of receptor-G_gust_ engagement. The C-terminally Nluc-tagged receptors were co-expressed with the Venus-labeled Gβγ dimer and wild-type Gα_gust_ in HEK-293A cells lacking endogenous Gα subtypes^30^. Due to the enhanced affinity of nucleotide-free G proteins for active-state receptors^41^, depleting endogenous nucleotides with apyrase and stimulating GPCRs with agonists should stabilize a high BRET state between GPCR-Nluc and fluorescently labeled G proteins. The addition of GTPγS would then dissociate productive GPCR–G protein complexes (leading to a decrease in BRET). In contrast, the addition of GTPγS to unproductive GPCR-G_gust_ pairings would either (i) rearrange or (ii) have no effect on preformed complexes, or (iii) enhance the assembly of these GPCR–G_gust_ complexes. All of these scenarios would lead to no change or an increase in BRET between GPCR-Nluc and Venus-Gβγ, and these unproductive complexes would not be able to promote Gα(GTP)- or Gβγ-mediated signal transduction (Fig. 6A). We performed this analysis with the presumed productive G_gust_-couplers H_3_R, BK_2_R and A_1_R, as well as the presumed unproductive G_gust_-couplers A_2A_R, H_2_R and 5HT_1A_R. GTPγS dissociated the receptor complexes with G_gust_ for H_3_R and A_1_R, while BK_2_R exhibited a two-phase temporal response, indicating initial complex rearrangement followed by dissociation (Fig. 6B). In contrast, enhanced complex formation or complex rearrangement was observed for A_2A_R, H_2_R and 5HT_1A_R (Fig. 6C). These results are consistent with our findings obtained using biosensors for G_gust_ heterotrimer dissociation (Ggust-CASE) and Gα_gust_-mediated signal transduction (Gα_gust_–PDE6γ interaction). Taken together, our pharmacological data show that G_gust_ can form both productive and unproductive complexes with GPCRs that are co-expressed in the same cell in the GIT or brain, when tested in HEK-293A cells. To understand whether these functionally distinct complexes are characterized by different modes of engagement of the GPCR and G_gust_, we next assessed the effect of pertussis toxin (PTX)-mediated ADP-ribosylation of the C-terminal α5 helix of Gα_gust_. As expected, Ggust-CASE BRET changes induced by the productive coupler A_1_R were blocked by PTX (Fig. 6D), compatible with the conventional mode of GPCR–G protein coupling involving the engagement of the α5 helix of Gα_gust_ with the open receptor core. In contrast, BRET increases mediated by the unproductive coupler H_2_R were insensitive to PTX (Fig. 6E). This data suggests that the unproductive H_2_R-G_gust_ complex does not depend on Gα_gust_ α5 helix interactions with the receptor’s transmembrane core (Fig. 6F)– a concept, which is further supported by cryo-EM structures of unproductive complexes of virus-encoded GPCRs with G_i1_^38,45^.

### Unproductive GPCR–G_gust_ complexes sequester receptors from other G proteins

**Fig. 7:**
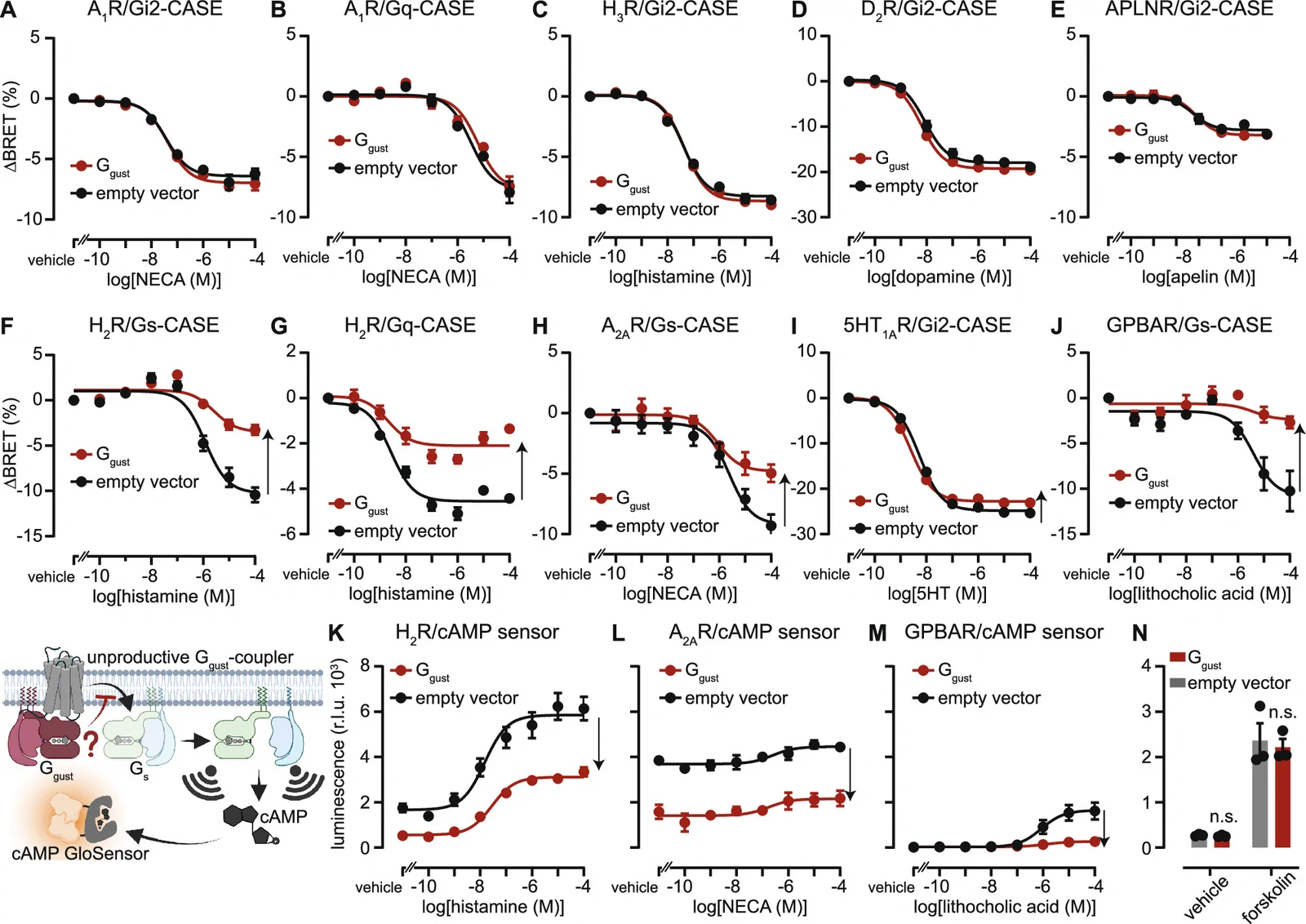
Unproductive GPCR–G_gust_ complexes suppress activation of and signal transduction by alternative G proteins. GPCR agonist concentration-dependent BRET changes induced in sensors of primary and secondary G proteins by productive G_gust_-couplers A_1_R (**A**: primary G protein G_i2_; **B**: secondary G protein G_q_), H_3_R (**C**: G_i2_), D_2_R (**D**: G_i2_) and APLNR (**E**: G_i2_) in the presence and absence of overexpressed G_gust_. GPCR agonist concentration-dependent BRET changes induced in sensors of primary and secondary G proteins by unproductive G_gust_-couplers H_2_R (**F**: primary G protein G_s(long)_; **G**: secondary G protein G_q_), A_2A_R (**H**: G_s(long)_), 5HT_1A_R (**I**: G_i2_) and GPBAR (**J**: G_s(long)_) in the presence and absence of overexpressed G_gust_. Constitutive and GPCR agonist-induced cAMP production induced by unproductive G_gust_-couplers H_2_R (**J**), A_2A_R (**K**) and GPBAR (**L**) in the presence and absence of overexpressed G_gust_. **N** Effect of G_gust_ overexpression on cAMP levels without co-expression of unproductive GPCRs, with and without forskolin treatment (100 μM). Data show mean ± SEM of three to six independent experiments conducted in HEK-293A cells (**A**–**J**) or HEK-293A cells devoid of endogenous Gα proteins and co-transfected with Gα_s(long)_ (**K**–**N**). Statistical significance between the plateau responses in (**A**–**M**) was tested using the extra-sum-of-squares F-test and is indicated with the black arrows (*p* < 0.05). Statistical difference between the G_gust_ and empty vector condition in (**N**) was tested using a two-sided 2-way ANOVA followed by Sidak’s multiple comparison (n.s.: *p* ≥ 0.05). Source data are provided as a Source Data file. cAMP: cyclic adenosine monophosphate. Scheme created in BioRender. Schihada, H. (2026) https://BioRender.com/h4iswy0.

**Fig. 8:**
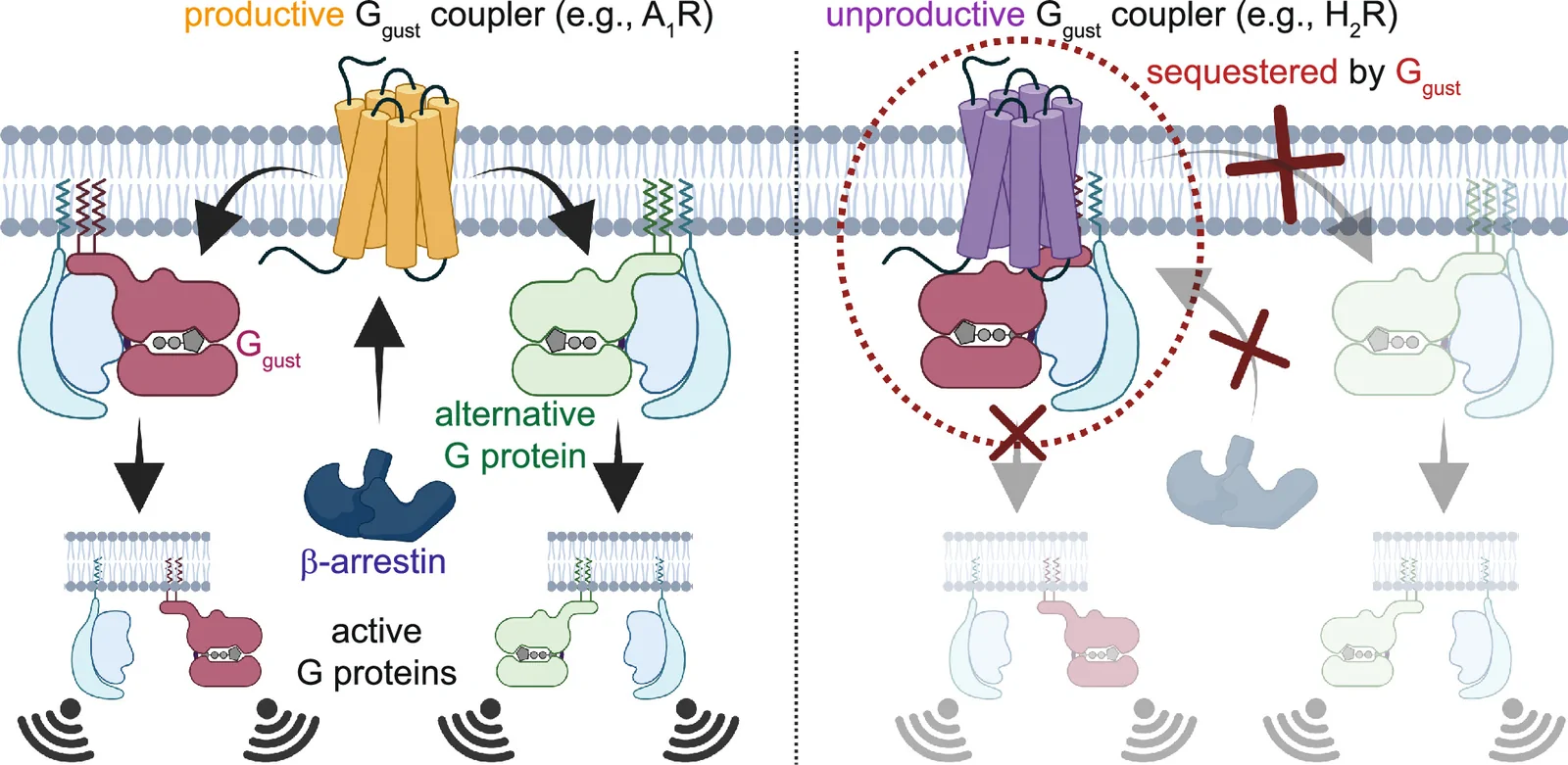
Cartoon representation of how G_gust_ sequesters GPCRs and suppresses alternative signaling pathways. Model for G_gust_-enabled suppression of GPCR-mediated signaling through primary G proteins. Created in BioRender. Schihada, H. (2026) https://BioRender.com/hquqts8.

The formation of unproductive GPCR–G_gust_ complexes may have important implications for signal transduction in cells mediated by other G proteins or GPCR coupling partners^38^. Therefore, we investigated the effect of G_gust_ heterotrimer (double dark Ggust-CASE) overexpression on H_2_R- and A_1_R-mediated activation of other G proteins and recruitment of β-arrestin2. While the activation of primary and secondary G proteins by productive G_gust_-couplers was not impaired by G_gust_ overexpression (Fig. 7A–E), the amplitude of G protein activation by unproductive G_gust_-couplers was substantially reduced in the presence of G_gust_ (Fig. 7F–J). G_gust_-mediated inhibition of G_s_ activation by H_2_R, A_2A_R and GPBAR directly translated into reduced constitutive and ligand-induced production of the second messenger cAMP (Fig. 7K–M). In contrast, overexpression of G_gust_ in the absence of these receptors did not affect intracellular levels of cAMP (Fig. 7N). Similarly, inhibition of alternative pathways was observed when receptor-mediated β-arrestin2 translocation to the plasma membrane was assayed with and without G_gust_ overexpression (Supplementary Fig. 14). Importantly, the surface expression levels of the H_2_R were similar in experiments with and without double dark Ggust-CASE co-expression (Supplementary Fig. 14G) and the raw BRET values confirmed the effects of G_gust_ overexpression independent of altering basal BRET values (Supplementary Fig. 14H). Overall, these findings, together with our previous data, suggest that unproductive GPCR–G_gust_ complexes sequester receptors away from alternative G proteins, thereby attenuating their activation. Consequently, the reduced release of Gβγ subunits limits the recruitment of cytosolic GRKs to activated receptors^48,49^, resulting in diminished β-arrestin engagement with phosphorylated receptors (Fig. 8). Mechanistically, the observation that receptors, such as the H_2_R, which form unproductive complexes with G_gust_, conventionally activate other G_i/o_ family members despite the high sequence similarity of the Gα subunits in their C-terminal region (Supplementary Fig. 15), suggests that structural determinants outside the Gα C-terminus contribute to the ability of receptors to promote productive nucleotide exchange of G_gust_.

## Discussion

Many GPCRs are able to couple to and activate more than just one type of G protein in a given cellular setting^33^. While the preferences of GPCRs for certain G protein subtypes have been a body of intense research in past decades, the possibility that different GPCRs can also tune G protein activity in different directions has, however, only recently gained attention with reports of unproductive GPCR-G protein engagements^38,45^. The present study shows that G_gust_ can couple to and be regulated by several non-tastant GPCRs when expressed in HEK-293A cells. Importantly, several receptors reduced basal G_gust_ activity and signal transduction. This observation complements recent reports of unproductive GPCR–G protein complexes, raising intriguing questions regarding the physiological consequences of these assemblies in the context of physiology and pathophysiology.

The G protein G_gust_ has long been exclusively viewed as a sensor of taste due to its coupling to tastant-binding GPCRs in gustatory cells. G_gust_’s roles in other parts of the human body remain poorly characterized. Our study begins to illuminate these poorly understood roles and suggests a dual mode of GPCR regulation in cells in which G_gust_ acts as a determinant of divergent signaling outcomes. We show that G_gust_ not only couples to non-tastant GPCRs, but that its interaction with GPCRs can be either productive – leading to enhanced signal transduction – or unproductive, which reduces basal G_gust_-dependent signaling and suppresses the activation of alternative signaling pathways. Previous co-expression analyses suggested that G_gust_ may couple to non-tastant GPCRs in pancreatic β-cells and the colon to promote central physiological responses, such as GLP1 release and insulin secretion, respectively^50,51^, but direct evidence for these interactions has been lacking until today.

Our findings raise the question of how suppression of G_gust_ signaling can occur mechanistically and what physiological consequences such inhibition of basal G_gust_ signaling and other pathways may have. Reduction of basal G_gust_ activity and signal transduction requires a sufficient level of basal G_gust_ signaling capacity in cells, as shown for other members of the G_i/o_ family, which all elicit high levels of GDP/GTP exchange without external stimulation in biochemical experiments^52,53^.

In fact, basal signaling activity of Gα_gust_ is required to maintain low levels of cAMP and insulin secretion in and from pancreatic β-cells^54^ and ensures low Ca^2+^ signaling in taste buds indicating that basal G_gust_ activity is of vital physiological importance across tissues. Productive and unproductive GPCRs coupling to G_gust_ could modulate the sensitivity of these cells prior to additional external (GPCR) stimuli. Likewise, G_gust_-mediated sequestration of GPCRs may serve as a physiological mechanism to prevent concurrent activation of parallel signaling pathways within the same cell in the presence of multiple agonists. Yet, further investigations in more native systems are required to understand the physiological importance of these complexes. In particular, the molecular stoichiometry of GPCRs and different Gα subtypes present in the same cell may influence whether G_gust_ couples to these GPCRs in a physiological setting. Because our experiments were performed in an overexpression system, the relative abundance of receptors and G proteins likely differs from that in native tissues and may therefore affect the formation of these complexes.

On a mechanistic level, it needs to be determined which molecular or atomistic components define a GPCR as a productive or unproductive interaction partner for G_gust_. In our profiling of 26 different GPCRs, we did not observe striking correlations between unproductive G_gust_ coupling and primary coupling preferences across the four G protein families (Fig. 3), suggesting that other determinants than those defining G protein coupling preference underlie the productivity of a given GPCR-G_gust_ interaction. While unproductive coupling of GPCRs to G_12_ is influenced by the C-terminal α5 helix of Gα_12_^38^, the observation that G_i1_ and G_o1_ – in contrast to G_gust_ – form productive complexes with H_2_R despite the high sequence similarity of the Gα subunits in their C-terminal region (Supplementary Fig. 15), suggests that also other regions influence the signaling capacity of G_gust_ after receptor engagement. Structural studies and mutational mapping of receptor-G_gust_ pairs could provide these mechanistic insights and open avenues to target these interactions.

Together, our work identifies the G protein G_gust_ as a broadly interacting G_i/o_-family member whose signaling output can be either enhanced or suppressed by non-tastant GPCRs, and which can function as a delimiter of alternative signaling pathways. Future studies combining mutational analysis, structural approaches, and experiments in native cell systems will be required to determine whether the regulatory principles uncovered here contribute to physiological processes, such as nutrient sensing and hormone secretion, in vivo.

## Methods

Reagents Apyrase, grade VI from potato, digitonin, and PEI (polyethyleneimine) were obtained from Sigma-Aldrich. GPCR ligands, forskolin, and D-luciferin potassium salt were purchased from MedChemExpress or Sigma-Aldrich. Furimazine and the Nano-Glo® HiBiT Extracellular Detection System were from Promega. GDPβS and GTPγS were from Jena Bioscience. Clobenpropit was from Tocris. Cell culture media (DMEM, HBSS, DPBS, FBS, penicillin/streptomycin, L-glutamine, Trypsin-EDTA and OptiMEM) were from Capricorn. All cloning reagents, except for oligonucleotides, were from NEB. Oligonucleotides were obtained from IDT. Cell culture flasks and white 96-well plates were from Greiner Bio-One.

Plasmids and molecular cloning: Plasmids encoding Gα_gust_-Nluc, Gα_11_-Nluc, Gα_o2_-Nluc, PDE6γ-cpVenus, p63RhoGEF-cpVenus, PDZ-RhoGEF-cpVenus, KB1694-cpVenus, KB1753-cpVenus and N-terminally HiBiT-labeled GPCRs were synthesized and subcloned into a pcDNA3.1(+) by Genscript. YFP-labeled Gα effector constructs were kindly provided by Moritz Bünemann (Marburg, Germany), and the plasmid encoding masGRK3ct-Nluc was kindly provided by Rob Leurs (Amsterdam, The Netherlands). Plasmids encoding β-arrestin2-RlucII and rGFP-CAAX were kindly provided by Michel Bouvier (Montreal, Canada). Ggust-CASE, G11-CASE and Go2-CASE were cloned by subcloning the respective Gα-Nluc sequences into the G_o1_-CASE template using Gibson assembly, thereby replacing Gα_o1_-Nluc. The point mutations in cpVenus and Nluc to generate dark Ggust-CASE and double dark Ggust-CASE were introduced into Ggust-CASE using site-directed mutagenesis. Gα_q_-Nluc was PCR-amplified from Gq-CASE^20^ and sub-cloned into pcDNA3.1(+) using Gibson assembly. Unlabeled Gα_gust_ was generated through blunt-ended ligation using Gα_gust_-Nluc in pcDNA3.1(+) as a template. Plasmid encoding Venus(NT)-Gβ_1_-Venus(CT)-Gγ_2_ was cloned by replacing the Gγ sequence fragment in Venus(NT)-Gβ_1_-Venus(CT)-Gγ_4_ (synthesized and sub-cloned into pcDNA3.1(+) by Twist Bioscience) using Gibson assembly. C-terminally Nluc-tagged GPCRs were cloned through PCR amplification of the HiBiT-GPCR fragments and insertion into an Nluc sequence-containing pcDNA3.1(+) template. All plasmids were confirmed by sequencing.

HEK-293A cells were from ThemoFisher (cat# R70507), and Gα knockout cells were kindly provided by Asuka Inoue^30^ (Kyoto, Japan). All cell lines were grown in Dulbecco’s modified Eagle’s medium (DMEM) supplemented with 2 mM glutamine, 10 % fetal calf serum, streptomycin (0.1 mg/mL), and penicillin (100 U/mL) at 37 °C with 5 % CO_2_. Absence of mycoplasma contamination was routinely confirmed by PCR.

Transient transfection and plating: Resuspended cells (300,000 cells/mL) were transfected in suspension with a total of 1 μg DNA/mL suspension using PEI (1 mg/ml stock solution; 3 μL PEI solution per μg DNA). The exact DNA ratios for co-expression of different proteins are stated in the respective methods sections. Cells mixed with the transfection reagents were seeded onto 96-well plates (100 mL cell suspension / well) and grown for 48 h at 37 °C with 5 % CO_2_. White plates were used for our BRET-based experiments.

Permeabilization procedure Cells grown in 96-well microtiter plates were washed with 120 μL HBSS and then incubated with 0.02 U/mL apyrase and 0.001% digitonin (w/v) dissolved in internal buffer (100 mM K^+^-aspartate, 30 mM KCl, 10 mM HEPES, 5 mM EGTA, 1 mM MgCl_2_, 10 mM NaCl, pH 7.35) for 25 minutes at room temperature. Prior to the experiments, permeabilized cells were then supplemented with 1 μM GDPβS and 1/1000 dilution of furimazine (only for BRET experiments).

Luminescence spectra recording of Ggust-CASE and dark Ggust-CASE in intact cells: HEK-293A cells were transfected with a plasmid encoding Ggust-CASE or dark Ggust-CASE. 48 hours post-transfection, cells were washed with 120 μL/well HBSS and incubated with 80 μL/well of a 1/1000 dilution of furimazine for 2 minutes at room temperature. Next, luminescence emission intensities were scanned with 1 nm resolution from 400 nm – 600 nm. All experiments were performed using a ClarioStar Plus plate reader with an integration time of 0.3 seconds.

Luminescence-based quantification of HiBiT-H_2_R surface expression: HEK-293A cells were transfected with 25% HiBiT-H_2_R plasmid, 25% pcDNA, and 50% pcDNA or double dark Ggust-CASE. 48 hours post-transfection, intact cells were washed with 120 μL/well HBSS and incubated with a 1/200 dilution of the Nano-Glo® HiBiT Extracellular Substrate and a 1/400 dilution of the LgBiT protein for 30 minutes at room temperature. Nluc emission was then quantified using a ClarioStar Plus plate reader with the following settings: λ(emission): 450-80 nm; integration time: 0.9 seconds.

Luminescence-based cAMP GloSensor experiments. To assess direct effects of G_gust_ on cAMP levels, HEK-293A cells lacking endogenous Gα proteins^30^ were transfected with 25% cAMP GloSensor -22F (Promega), 25% Gα_gust_ or pcDNA, and 50% pcDNA. 25% cAMP GloSensor -22F (Promega), 25% Gα_gust_ or pcDNA, and 50% pcDNA. To assess the effect of unproductive GPCR–G_gust_ complexes on cAMP production through Gα_s_-mediated pathways, the same cells were transfected with 25% cAMP GloSensor -22F (Promega), 25% Gα_s(long)_, 10% GPCR, and 40% double dark Ggust-CASE or pcDNA. To determine whether productive GPCR–G_gust_ could alter cAMP, these cells were transfected with 25% cAMP GloSensor -22F (Promega), 25% Gα_gust_, 10% GPCR, and 40% pcDNA. 48 hours post-transfection, these cells were washed with 120 mL/well HBSS and incubated with 72 μL/well of a 1 mM D-luciferin potassium salt for 20 minutes at room temperature. To assess direct effects of G_gust_ on cAMP levels 1 mM forskolin or vehicle control was then added right before the measurement. Next, luminescence was recorded in two consecutive reads before (serial dilutions of) agonists or vehicle control were added, followed by additional luminescence reads. The peak response of the time course data was considered for the generation of concentration response curves. All experiments were performed using a ClarioStar Plus plate reader set to 28 °C. GloSensor luminescence intensity was quantified with a 580-80 nm monochromator; an integration time of 1.1 seconds and spiral averaging with a scan diameter of 5 mm.

BRET measurements in intact cells HEK-293A cells were performed with 50% receptor plasmid and 50% G-CASE, or a mix of 10% Gα-Nluc or dark Ggust-CASE with 40% PDE6γ-cpVenus or any of the other YFP/cpVenus-labeled potential effectors. For experiments detecting the release of Gβγ dimers from G_gust_ heterotrimers, HEK-293A cells lacking endogenous Gα proteins^30^ were transfected with 50% receptor, 24% unlabeled Gα_gust_, 24% Venus-Gβ_1_γ_2_, and 2% masGRK3ct-Nluc. For experiments assessing the effect of pertussis toxin on G_gust_ activity, HEK-293A cells were transfected with 50% receptor plasmid, 45% Ggust-CASE, and 5% PTX-S1 or pcDNA. For competition experiments, 25% receptor plasmid was mixed with 25% G-CASE or 5% β-arrestin2-RlucII and 20% rGFP-CAAX,^56^, along with 50% double dark Ggust-CASE or pcDNA. To assess the potential involvement of endogenously expressed GPCRs on Ggust activation and β-arrestin2 translocation, 50% pcDNA was co-transfected with 50% Ggust-CASE or a mix of 10% β-arrestin2-RlucII and 40% rGFP-CAAX. 48 hours post-transfection, intact cells were washed with 120 μL/well HBSS and incubated with 72 μL/well of a 1/1000 dilution of furimazine for 2 minutes at room temperature. When the effect of antagonists on GPCR agonist-induced G_gust_ activation was assessed, the indicated antagonist concentrations or vehicle control were added to the cells together with furimazine. Next, BRET was recorded in three consecutive reads before (serial dilutions of) agonists or vehicle control were added, followed by additional BRET reads. The peak response of the time course data was considered for the generation of concentration response curves. All experiments were performed using a ClarioStar Plus or PheraStar FSX plate reader. For experiments with the BRET donor Nluc and YFP or cpVenus as BRET acceptors, the following settings were used: λ(donor emission): 450-80 nm; λ(acceptor emission): 535-30 nm; integration time: 0.3 seconds. For experiments with the BRET donor RlucII and GFP as the BRET acceptor, the following settings were used: λ(donor emission): 400-100 nm; λ(acceptor emission): 520-30 nm; integration time: 0.9 seconds.

BRET measurements in permeabilized cells: HEK293A cells were transfected with 50% pcDNA and Ggust-CASE, or 10% dark Ggust-CASE with 40% PDE6γ-cpVenus. 48 hours post-transfection, cells were permeabilized and nucleotide-depleted by incubating with 0.001 % (m/v) digitonin and 0.02 U/ml apyrase for 25 minutes at room temperature. Then, permeabilized cells were loaded with 1 μM GDPβS and 1/1000 furimazine. Next, BRET was recorded in several consecutive reads before 10 μM GTPγS or vehicle control was added with automated injectors, followed by additional BRET reads. All experiments were performed using a PheraStar FSX plate reader with the following settings: λ(donor emission): 450-80 nm; λ(acceptor emission): 535-30 nm; integration time: 0.3 seconds.

Nucleotide-sensitivity of GPCR-Nluc/G_gust_ complexes in permeabilized HEK-293A cells lacking endogenous Gα proteins^30^ were transfected with 25% GPCR-Nluc, 25% unlabeled Gα_gust_, and 50% Venus-Gβ_1_γ_2_. 48 hours post-transfection, cells were permeabilized, loaded with 1/1000 furimazine, and saturated with GPCR agonists (i.e., 10 μM bradykinin for BK_2_R; 100 μM NECA for A_1_R and A_2A_R; 100 μM serotonin for 5HT_1A_R; 100 μM histamine for H_2_R and H_3_R). After five minutes of incubation at room temperature, BRET was recorded in several consecutive reads before 10 μM GTPγS or vehicle control was added with automated injectors, followed by additional BRET reads. All experiments were performed using a PheraStar FSX plate reader with the following settings: λ(donor emission): 450-80 nm; λ(acceptor emission): 535-30 nm; integration time: 0.3 seconds.

Quantification and statistical analysis: BRET was defined as acceptor emission over donor emission. All baseline BRET values were averaged to obtain BRET_basal_. Before applying further normalization or correction, the raw values were compared to assess potential differences in baseline conditions among the samples. If no differences were detected, the raw values were then subjected to further normalization and correction. Raw ΔBRET at any given time point t was calculated as follows: raw ΔBRET_t_ = ((BRET_t_-BRET_basal_)/BRET_basal_)*100. The average raw ΔBRET_t_ of all vehicle-treated technical replicates was calculated for any given time point t. These values were subtracted from all raw ΔBRET_t_ to obtain corrected ΔBRET_t_ values (named only “ΔBRET” in figure axes for simplicity) for each test condition. Corrected ΔBRET values were plotted over time, and time points showing a saturated response for the highest ligand concentrations tested were selected for the generation of concentration-response curves. Semi-logarithmic concentration-response curves were plotted and fitted using a sigmoidal concentration–response model (with the Hill slope fixed to +1 or −1). For the data in Fig. 3, non-G_gust_-couplers (nc) were identified by comparing a linear model with a zero slope to a sigmoidal concentration–response model using an extra-sum-of-squares-F test (*p* > 0.05).

Tissue-level GPCR expression analysis in the small intestine and duodenum. We queried the Human Protein Atlas with the following prompts: (1) protein_class: G-protein coupled receptors; Any AND normal_expression: Duodenum; Any; (2) protein_class: G-protein coupled receptors; Any AND normal_expression: Small intestine; Any. The results were exported as TSV files and imported into Microsoft Excel for inspection (compiled in Supplementary Data 1).

Single-cell-level co-expression analysis of GPCRs with Gα_gust_. Publicly available single-cell RNA-seq data were obtained from the Human Protein Atlas^15^. Data processing and analysis were performed using R. Read count tables for 36 datasets and the respective metadata were downloaded before co-expression analysis of *GNAT3* and the selected GPCRs. A gene was considered expressed if the read count was greater than zero. Cells were classified into four categories: both genes expressed, only *GNAT3* expressed, only the GPCR expressed, or neither gene expressed. Cell counts were calculated for each cell subtype and tissue.

### Reporting summary

Further information on research design is available in the Nature Portfolio Reporting Summary linked to this article.

## Supplementary information

Supplementary Information

Description of Additional Supplementary Files

Supplementary Data 1

Supplementary Data 2

Reporting Summary

Transparent Peer Review file

## Source data

Source Data File 1

Source Data File 2

## Data Availability

All data generated or analyzed during this study are provided in the supplementary information files and source data files. Source data are provided with this paper.
