# Supplementary material for: Functional diversity of GPCR–Gustducin complexes controls signaling output and suppresses alternative pathways
Source: Nat Commun. 2026 Nov 14;17:11681. doi: 10.1038/s41467-026-79528-z (PMC13769768; doi:10.1038/s41467-026-79528-z)
Supplement: Supplementary file 1 [file 41467_2026_79528_MOESM1_ESM.pdf]

Supplementary Information

for

Functional diversity of GPCR-Gustducin complexes controls signaling output and suppresses alternative pathways

Victoria Jaime Arce[1,*], Mira Lettmann[1,*], Mona Stefanie Tögl[1,*], Santiago Javier Bonn Garcia[1], Max Bauer[1], Maike Neukirch[1], Olivia Kleinstück[1], Anastasia Langolf[1], Isabell Kaczmarek[1,2], Daniel Hilger[1], Hannes Schihada[1,#]

[1]: Department of Pharmacy, Institute of Pharmaceutical Chemistry, Marburg University, Germany

[2]: Rudolf Schönheimer institute of Biochemistry, Leipzig University, Germany

*: contributed equally

[#]: correspondence to hannes.schihada@uni-marburg.de

Supplementary Figure 1

Gα_{gust}-Nluc:
MGSGISSESKESAKRSKELEKKLQEDAERDARTVKLLLLGAGESGKSTIVKQMKIIHK
NGYSEQECMEFKAVIYSNTLQSILAIVKAMTTLGIDYVNPRSAEDQRQLYAMANTLED
SGGGGTRSVFTLEDFVGDWRQTAGYNLDQVLEQGGVSSLFQNLGVSVTPIQRIVLS
GENGLKIDIHVIIPYEGLSGDQMGQIEKIFKVVYPVDDHHFKVILHYGTLVIDGVTPNMI
DYFGRPYEGIAVFDGKKITVTGTLWNGNKIIDERLINPDGSLLFRVTINGVTGWRLCE
RILATGGGGSGGMTPQLAEVIKRLWRDPGIQACFERASEYQLNDSAAYYLNDLDRIT
ASGYVPNEQDVLHSRVKTTGIIETQFSFKDLHFRMFDVGGQRSERKKWIHCFEGVT
CIIFCAALSAYDMVLVEDEEVNRMHESLHLFNSICNHKYFSTTSIVLFLNKKDIFQEKV
TKVHLSICFPEYTGPNTFEDAGNYIKNQFLDLNLKKEDKEIYSHMTCATDTQNVKFVF
DAVTDIIIKENLKDCGLF*

Gβ_3-T2A-cpVenus-Gγ_9:

MGEMEQLRQEAEQLKKQIADARKACADVTLAELVSGLEVVGRVQMRTRRTLRGHL
AKIYAMHWATDSKLLVSASQDGKLIVWDSYTTNKVHAIPLRSSWVMTCAYAPSGNFV
ACGGLDNMCSIYNLKSREGNVKVSRELSAHTGYLSCCRFLDDNNIVTSSGDTTCAL
WDIETGQQKTVFVGHTGDCMSLAVSPDFNLFISGACDASAKLWDVREGTCRQTFT
GHESDINAICFFPNGEAICTGSDDASCRLFDLRADQELICFSHESIICGITSVAFSLSG
RLLFAGYDDFNCNVWDSMKSERVGILSGHDNRVSCLGVTADGMAVATGSWDSFLKI
WNEGRGSLLTCGDVEENPGPTGMDGGVQLADHYQQNTPIGDGPVLLPDNHYLSY
QSALSKDPNEKRDHMVLLEFVTAAGITLGMDELYKGGSGGMVSKGEELFTGVVPILV
ELDGDVNGHKFSVSGEGEGDATYGKLTLKLICTTGKLPVPWPTLVTTLGYGLQCFAR
YPDHMKQHDFFKSAMPEGYVQERTIFFKDDGNYKTRAEVKFEGDTLVNRIELKGIDF
KEDGNILGHKLEYNYNSHNVYITADKQKNGIKANFKIRHNIESGLRSAQDLSEKDLLK
MEVEQLKKEVKNTRIPISKAGKEIKEYVEAQAGNDPFLKGIPEDKNPFKEKGGCLIS*

Supplementary Figure 1. Amino acid sequence of G$_{gust}$-CASE. Amino acid sequences of Gα_{gust}-Nluc and Gβ_3-T2A-cpVenus-Gγ_9.

Supplementary Figure 2

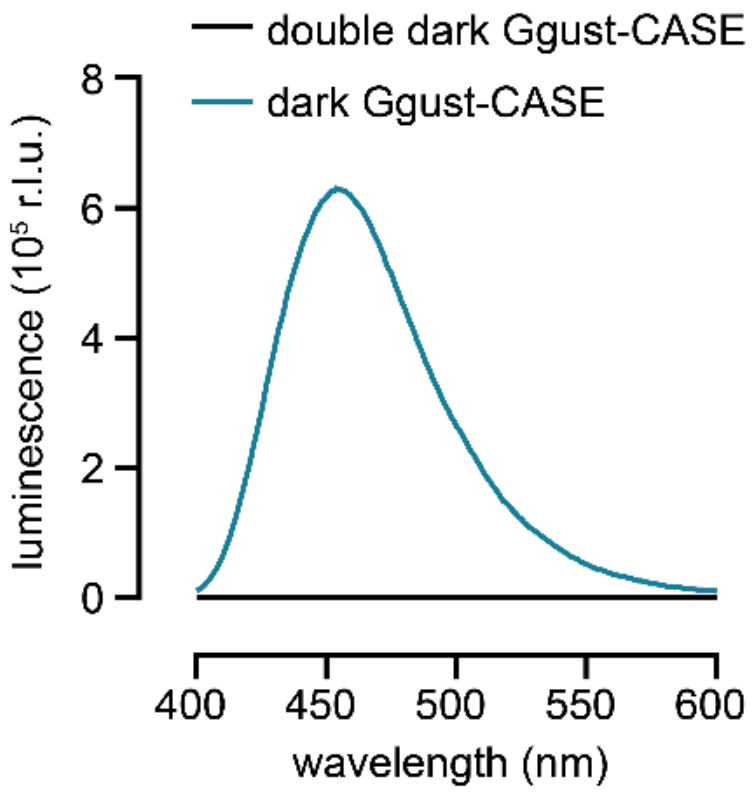

Supplementary Figure 2. Luminescence emission spectra of dark Ggust-CASE and double dark Ggust-CASE. Data represents the emission intensity in one replicate conducted in HEK-293A cells transiently transfected with plasmids encoding the indicated proteins.

Supplementary Figure 3

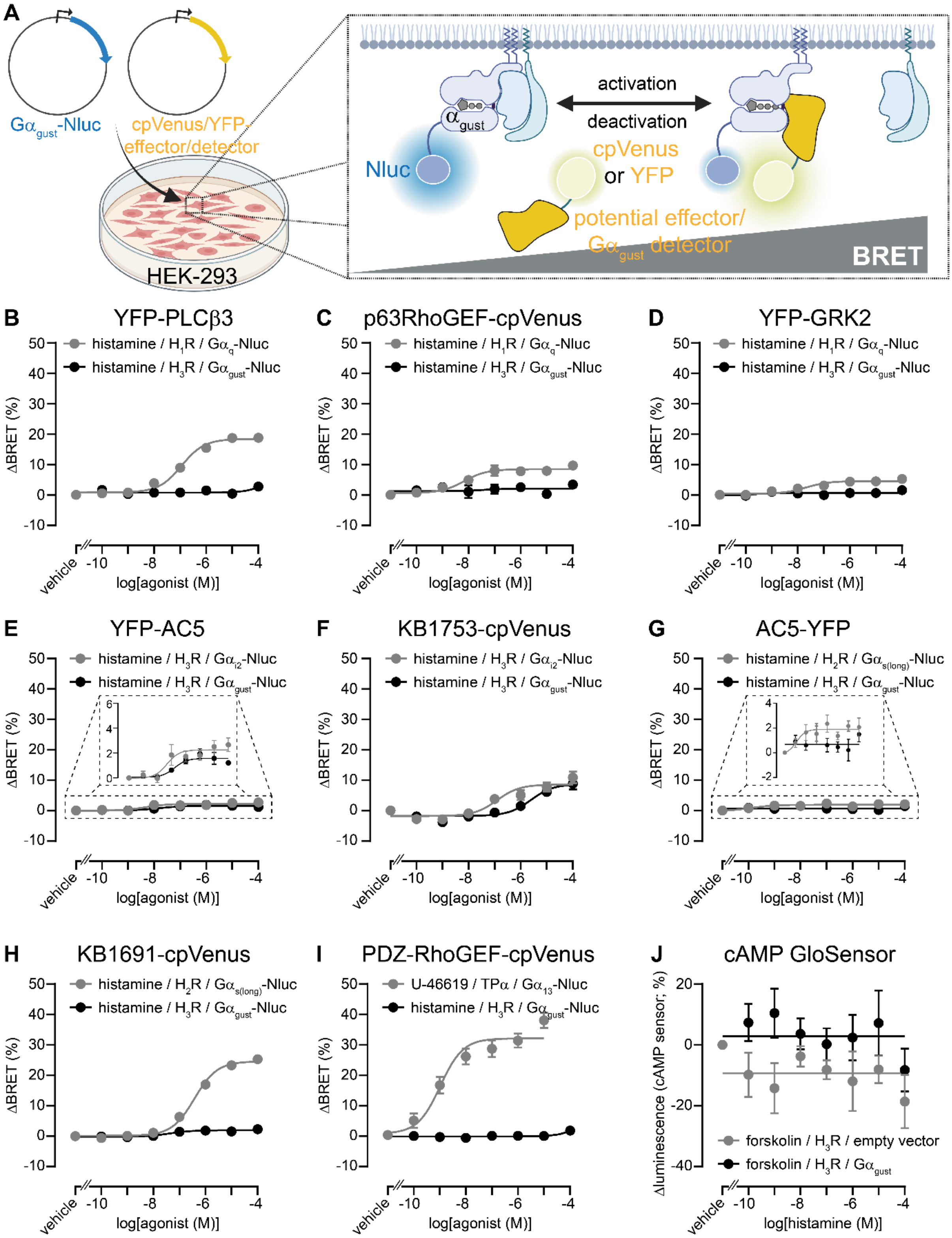

Supplementary Figure 3. Testing Gα effector proteins for interactions with $G\alpha_{gust}$ and modulation of cAMP levels. A) Scheme of the BRET-based protein-protein interaction assay. **B-I)** Concentration-dependent BRET changes induced by GPCR agonists. **J)** Concentration-dependent luminescence changes induced by histamine in forskolin-pretreated cells (100 μM) expressing the cAMP GloSensor, the H_3R and $G\alpha_{gust}$. Data represent mean ± SEM of three to five biological replicates conducted in HEK-293A cells (B-I) or gene-edited HEK-293A cells lacking endogenous Gα proteins (J) transiently transfected with plasmids encoding the indicated proteins.

Supplementary Figure 4

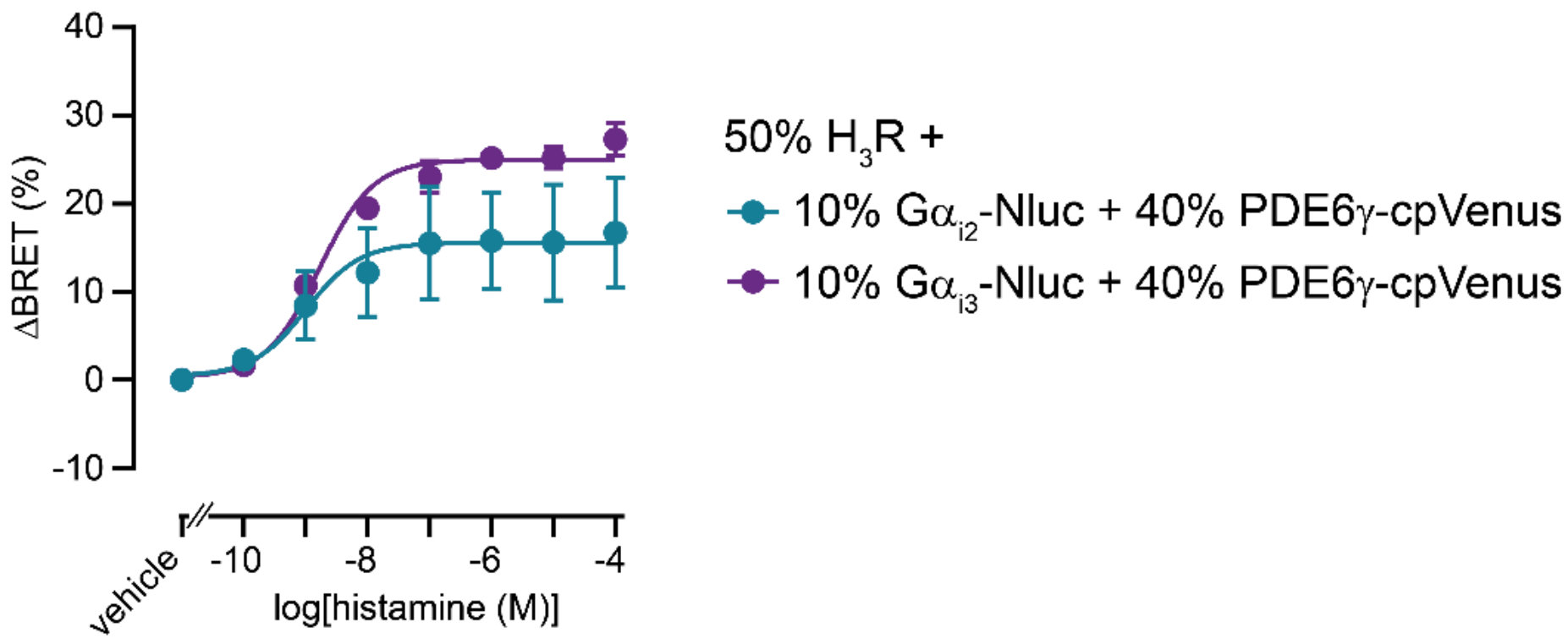

Supplementary Figure 4. Interaction of other G$\alpha_{i/o}$ protein members with PDE6γ. Histamine-induced, concentration-dependent BRET changes in HEK-293A cells transiently transfected with H_3R, Gα_{i2}- or Gα_{i2}-Nluc and PDE6γ-cpVenus. Data show mean ± SEM of three independent experiments.

Supplementary Figure 5

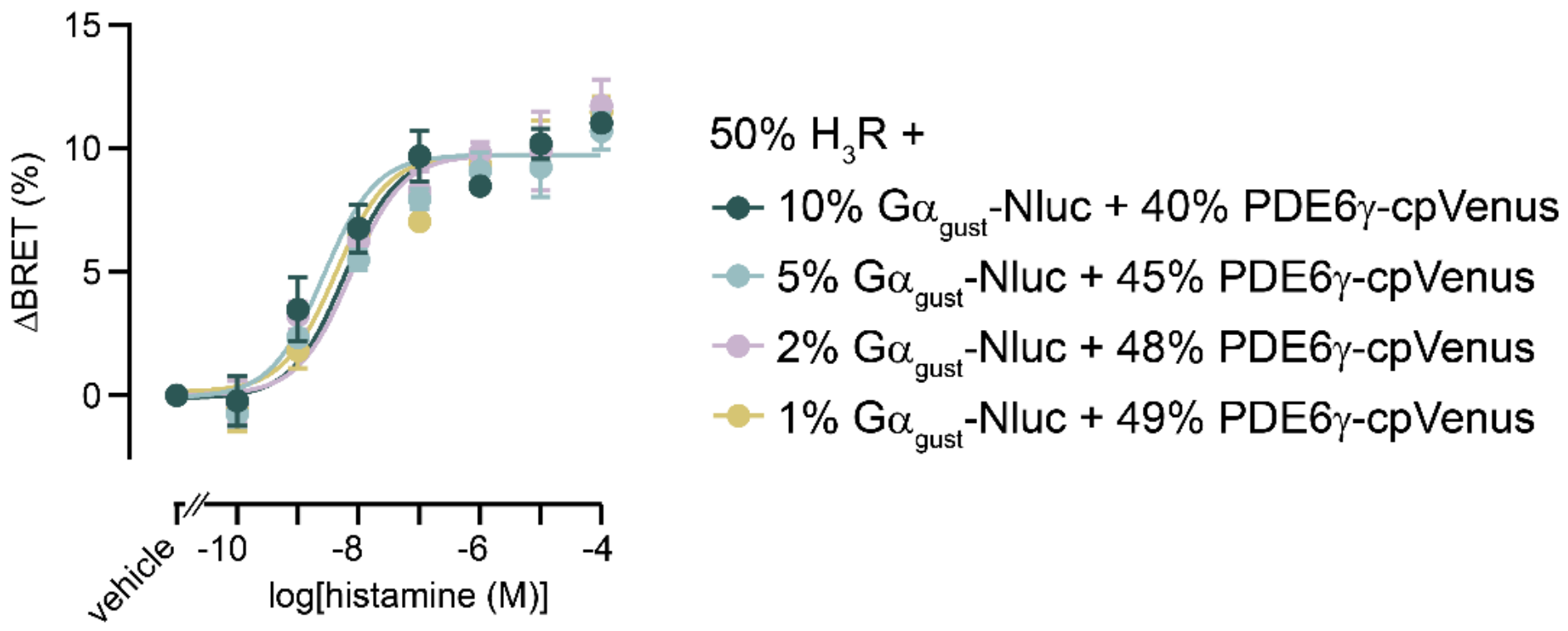

Supplementary Figure 5. BRET donor/acceptor titration for the $G\alpha_{gust}$–PDE6γ interaction assay. Histamine-induced, concentration-dependent BRET changes in HEK-293A cells transiently transfected with different plasmid ratios of H_3R, $G\alpha_{gust}$-Nluc and PDE6γ-cpVenus. Data show mean ± SEM of three independent experiments.

Supplementary Figure 6

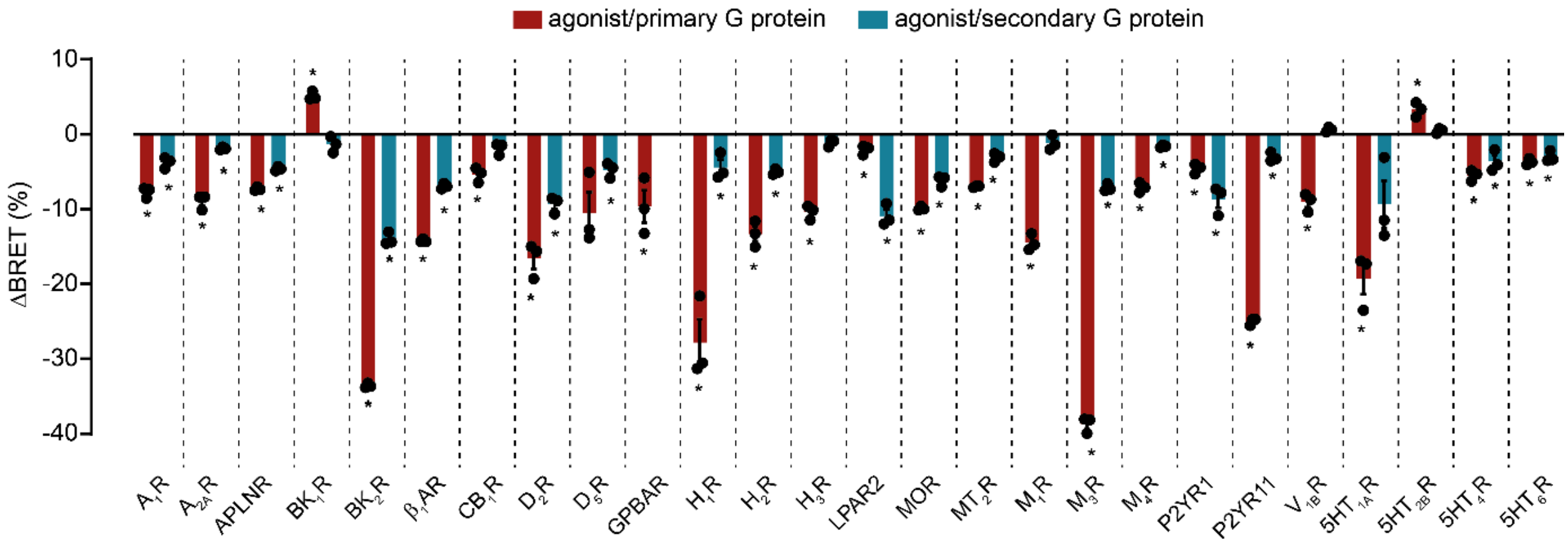

GPCR	agonist (concentration in μM)	G-CASE for primary G protein	G-CASE for secondary G protein
A_1R	NECA (100)	Gi2-CASE	Gq-CASE
$A_{2A}R$	NECA (100)	Gs(long)-CASE	Gq-CASE
APLNR	[Pyr-]apelin-13 (10)	Gi2-CASE	Gq-CASE
BK_1R	bradykinin (10)	Gq-CASE	Gi2-CASE
BK_2R	bradykinin (10)	Gq-CASE	G13-CASE
β_1AR	isoprenaline (100)	Gs(long)-CASE	Gq-CASE
CB_1R	CP-55940 (10)	Gi2-CASE	G13-CASE
D_2R	dopamine (100)	Gi2-CASE	Gq-CASE
D_5R	dopamine (100)	Gi2-CASE	Gq-CASE
GPBAR	lithocholic acid (100)	Gs(long)-CASE	
H_1R	histamine (100)	Gq-CASE	Gi2-CASE
H_2R	histamine (100)	Gs(long)-CASE	Gq-CASE
H_3R	histamine (100)	Gi2-CASE	Gs(long)-CASE
LPAR2	lysophosphatidic acid (10)	Gi2-CASE	G13-CASE
MOR	DAMGO (10)	Gi2-CASE	Gq-CASE
MT_2R	melatonin (100)	Gi2-CASE	Gq-CASE
M_1R	acetylcholine (100)	Gq-CASE	Gi2-CASE
M_3R	acetylcholine (100)	Gq-CASE	Gi2-CASE
M_4R	acetylcholine (100)	Gi2-CASE	Gq-CASE
P2YR1	ATP (100)	Gi2-CASE	Gq-CASE
P2YR11	ATP (100)	Gq-CASE	Gs(long)-CASE
$V_{1B}R$	AVP (10)	Gq-CASE	Gi2-CASE
$5HT_{1A}R$	5HT (100)	Gi2-CASE	Gq-CASE
$5HT_{2B}R$	5HT (100)	Gq-CASE	Gi2-CASE
$5HT_4R$	5HT (100)	Gs(long)-CASE	Gq-CASE
$5HT_6R$	5HT (100)	Gs(long)-CASE	Gq-CASE

Supplementary Figure 6. Activation of primary and secondary G proteins by GPCRs included in this study. Agonist-induced BRET changes in G-CASE sensors of primary and secondary G proteins (according to gpcrdb.org) in HEK-293A cells transiently transfected with the indicated GPCR/G-CASE combination. Data show mean ± SEM of three independent experiments. Statistical significance against the hypothetical value of zero was tested using the one-sample t-test (*$p < 0.05$).

Supplementary Figure 7

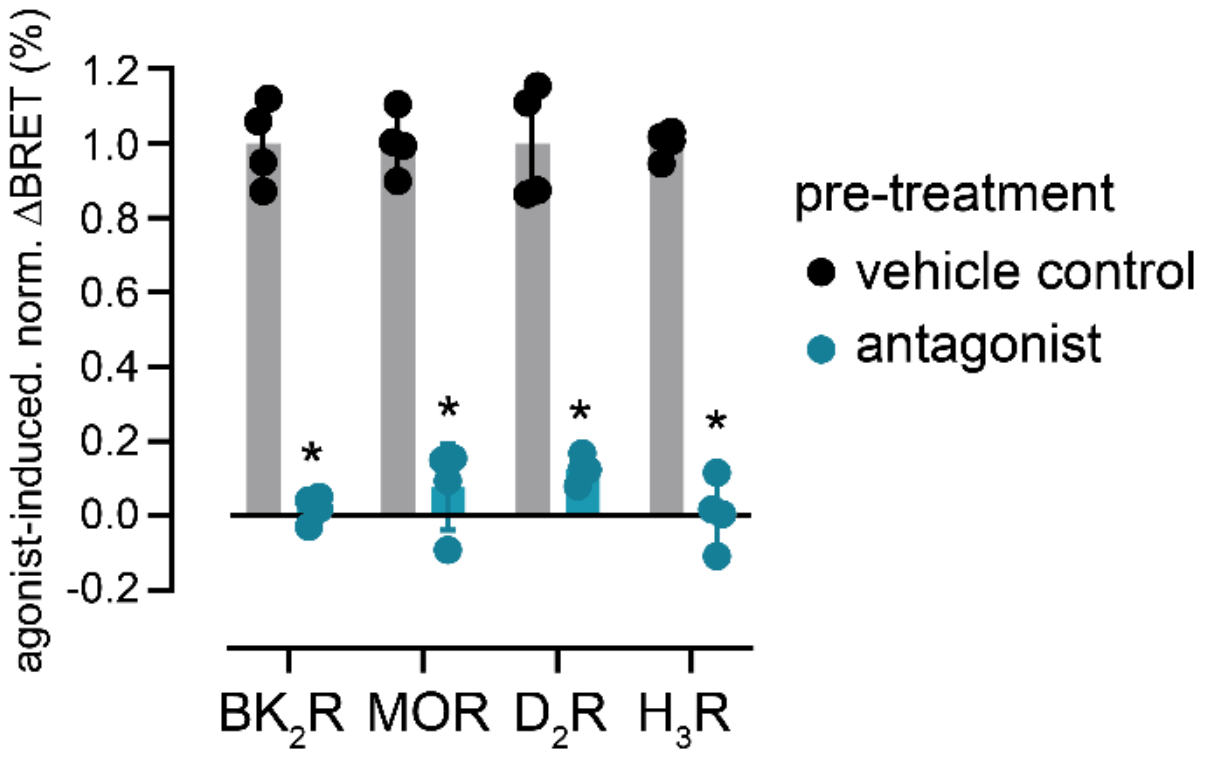

GPCR	agonist		antagonist	
	compound	concentration (nM)	compound	concentration (μM)
BK_2R	bradykinin	10	icatibant acetate	10
MOR	DAMGO	100	naloxone	10
D_2R	dopamine	100	risperidone	10
H_3R	histamine	1000	clobenpropit	10

Supplementary Figure 7. Antagonists block Ggust-CASE activation induced by productive G_{gust}-couplers. Normalized, agonist-induced BRET changes in HEK-293A cells transiently transfected with Ggust-CASE and the indicated GPCRs, with and without antagonist pre-treatment. Data show mean ± SEM of four independent experiments. Statistical significance against vehicle control was tested using Two-way ANOVA followed by Fisher's LSD multiple comparison (*$p < 0.0001$).

Fig. S8

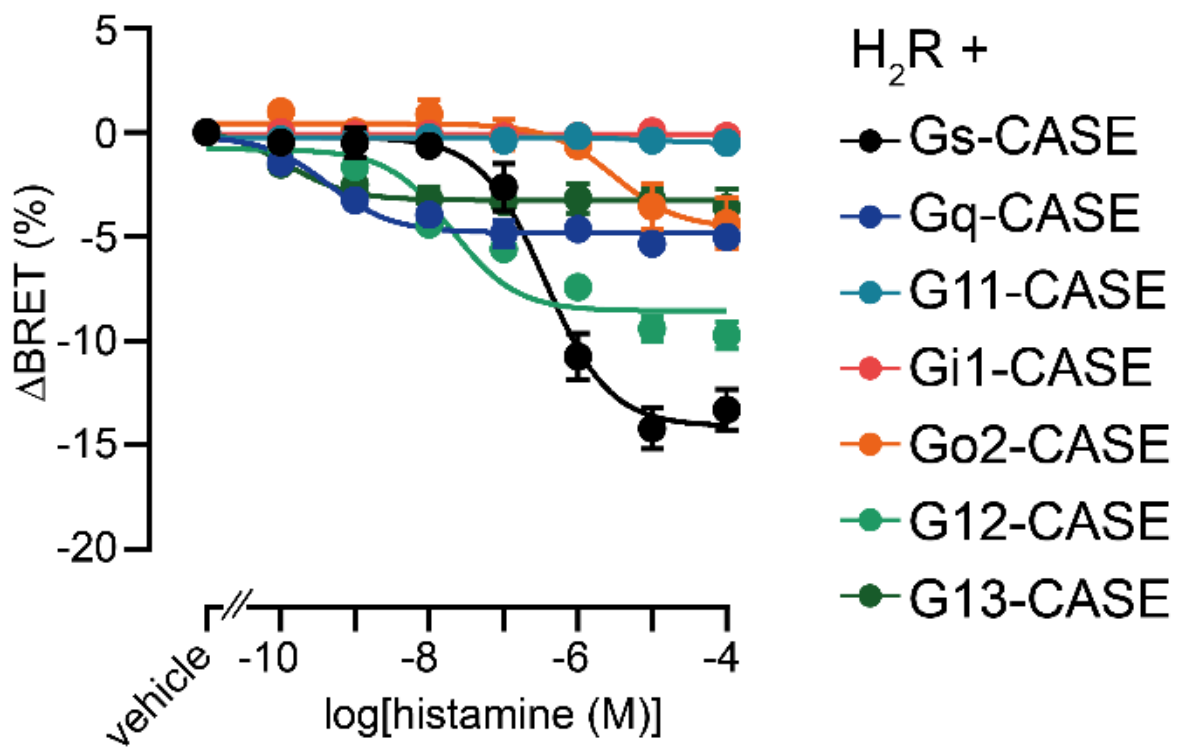

Supplementary Figure 8. H_2R-mediated G-CASE responses. Histamine-induced, concentration-dependent BRET changes in HEK-293A cells transiently transfected with the H_2R and different G protein biosensors. Data show mean ± SEM of three independent experiments.

Supplementary Figure 9

A

Gα$_{11}$-Nluc:
MTLESMMACCLSDEVKESKRINAEIEKQLRRDKRDARRELKLLLLGTGESGKSTFIK
QMRIIHGAGYSEEDKRGFTKLVYQNIFTAMQAMIRAMETLKILYKYEQNKANALLIRE
VDVEKVTTFEHQYVSAIKTLWEDPGIQECYDRRREYQLSDSAKYYLTDVDRIATLGY
LPTQQDVLRVRVPTTGIIEYPFDLENIIFRMVDVGGQRSERRKWIHCFENVTSIMFLV
ALSEYDQVLVESDNESGGGGTRSVFTLEDFVGDWRQTAGYNLDQVLEQGGVSSLF
QNLGVSVTPIQRIVLSGENGLKIDIHVIIPYEGLSGDQMGQIEKIFKVVYPVDDHHFKVI
LHYGTLVIDGVTPNMIDYFGRPYEGIAVFDGKKITVTGTLWNGNKIIDERLINPDGSLL
FRVTINGVTGWRLCERILATGGGGSNRMEESKALFRTIITYPWFQNSSVILFLNKKDL
LEDKILYSHLVDYFPEFDGPQRDAQAAREFILKMFVDLNPDSDKIIYSHFTCATDTENI
RFVFAAVKDTILQLNLKEYNLV*

Gβ$_3$-T2A-cpVenus-Gγ$_9$:

MGEMEQLRQEAEQLKKQIADARKACADVTLAELVSGLEVVGRVQMRTRRTLRGHL
AKIYAMHWATDSKLLVSASQDGKLIVWDSYTTNKVHAIPLRSSWVMTCAYAPSGNFV
ACGGLDNMCSIYNLKSREGNVKVSRELSAHTGYLSCCRFLDDNNIVTSSGDTTCAL
WDIETGQQKTVFVGHTGDCMSLAVSPDFNLFISGACDASAKLWDVREGTCRQTFT
GHESDINAICFFPNGEAICTGSDDASCRLFDLRADQELICFSHESIICGITSVAFSLSG
RLLFAGYDDFNCNVWDSMKSERVGILSGHDNRVSCLGVTADGMAVATGSWDSFLKI
WNEGRGSLLTCGDVEENPGPTGMDGGVQLADHYQQNTPIGDGPVLLPDNHYLSY
QSALSKDPNEKRDHMVLLEFVTAAGITLGMDELYKGGSGGMVSKGEELFTGVVPILV
ELDGDVNGHKFSVSGEGEGDATYGKLTLKLICTTGKLPVPWPTLVTTLGYGLQCFAR
YPDHMKQHDFFKSAMPEGYVQERTIFFKDDGNYKTRAEVKFEGDTLVNRIELKGIDF
KEDGNILGHKLEYNYNSHNVYITADKQKNGIKANFKIRHNIESGLRSAQDLSEKDLLK
MEVEQLKKEVKNTRIPISKAGKEIKEYVEAQAGNDPFLKGIPEDKNPFKEKGGCLIS*

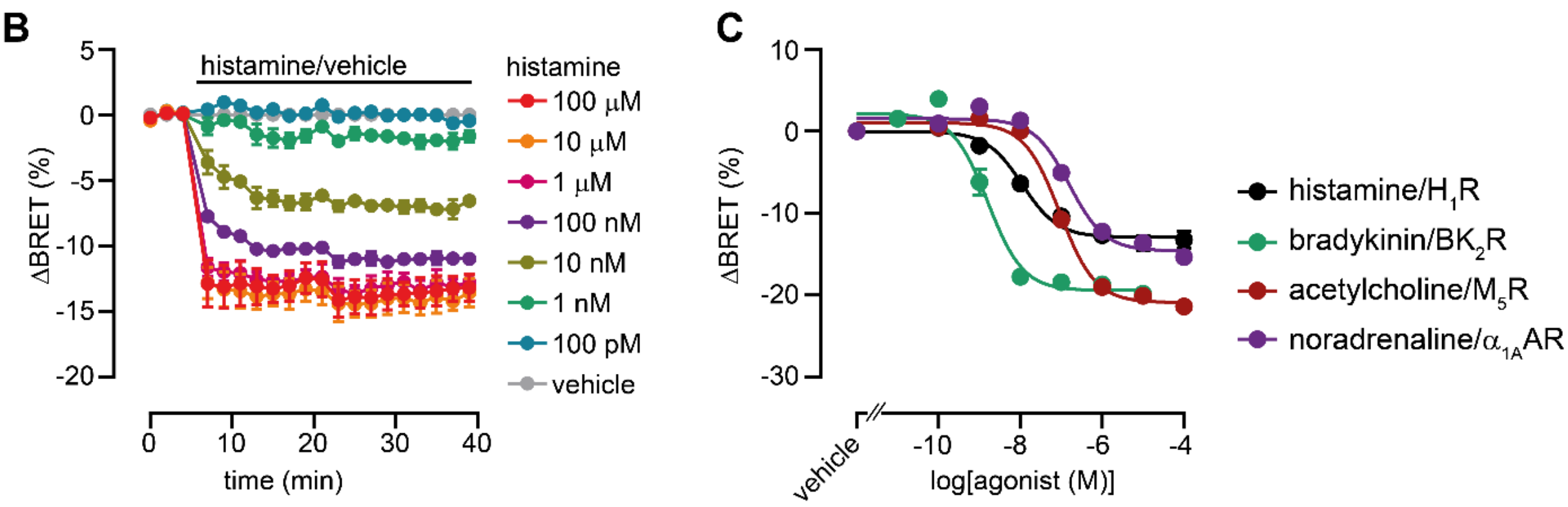

Supplementary Figure 9. Design and validation of G11-CASE. A) Amino acid sequence of G11-CASE. **B)** Time course of the histamine-induced BRET changes in HEK-293A cells that have been transiently transfected with the H_1R and G11-CASE. **C)** Concentration-dependent BRET changes induced by GPCR agonists in HEK-293A cells that have been transiently transfected with the indicated GPCR and G11-CASE. Data show mean ± SEM of three independent experiments.

Supplementary Figure 10

Gα$_{o2}$-Nluc:

MGCTLSAEERAALERSKAIEKNLKEDGISAAKDVKLLLLGAGESGKSTIVKQMKIIHE
DGFSGEDVKQYKPVVYSNTIQSLAAIVRAMDTLSGGGGTRSVFTLEDFVGDWRQTA
GYNLDQVLEQGGVSSLFQNLGVSVTPIQRIVLSGENGLKIDIHVIIPYEGLSGDQMGQ
IEKIFKVVYPVDDHHFKVILHYGTLVIDGVTPNMIDYFGRPYEGIAVFDGKKITVTGTL
WNGNKIIDERLINPDGSLLFRVTINGVTGWRLCERILATGGGGSGIEYGDKERKADA
KMVCDVVSRMEDTEPFSAELLSAMMRLWGDSGIQECFNRSREYQLNDSAKYYLDS
LDRIGAADYQPTEQDILRTRVKTTGIVETHFTFKNLHFRLFDVGGQRSERKKWIHCF
EDVTAIIFCVALSGYDQVLHEDETTNRMHESLKLFDSICNNKWFTDTSIILFLNKKDIF
EEKIKKSPLTICFPEYTGPSAFTEAVAYIQAQYESKNKSAHKEIYTHVTCATDTNNIQF
VFDAVTDVIIAKNLRGCGLY*

Gβ$_3$-T2A-cpVenus-Gγ$_9$:

MGEMEQLRQEAEQLKKQIADARKACADVTLAELVSGLEVVGRVQMRTRRTLRGHL
AKIYAMHWATDSKLLVSASQDGKLIVWDSYTTNKVHAIPLRSSWVMTCAYAPSGNFV
ACGGLDNMCSIYNLKSREGNVKVSRELSAHTGYLSCCRFLDDNNIVTSSGDTTCAL
WDIETGQQKTVFVGHTGDCMSLAVSPDFNLFISGACDASAKLWDVREGTCRQTFT
GHESDINAICFFPNGEAICTGSDDASCRLFDLRADQELICFSHESIICGITSVAFSLSG
RLLFAGYDDFNCNVWDSMKSERVGILSGHDNRVSCLGVTADGMAVATGSWDSFLKI
WNEGRGSLLTCGDVEENPGPTGMDGGVQLADHYQQNTPIGDGPVLLPDNHYLSY
QSALSKDPNEKRDHMVLLEFVTAAGITLGMDELYKGGSGGMVSKGEELFTGVVPILV
ELDGDVNGHKFSVSGEGEGDATYGKLTLKLICTTGKLPVPWPTLVTTLGYGLQCFAR
YPDHMKQHDFFKSAMPEGYVQERTIFFKDDGNYKTRAEVKFEGDTLVNRIELKGIDF
KEDGNILGHKLEYNYNSHNVYITADKQKNGIKANFKIRHNIESGLRSAQDLSEKDLLK
MEVEQLKKEVKNTRIPISKAGKEIKEYVEAQAGNDPFLKGIPEDKNPFKEKGGCLIS*

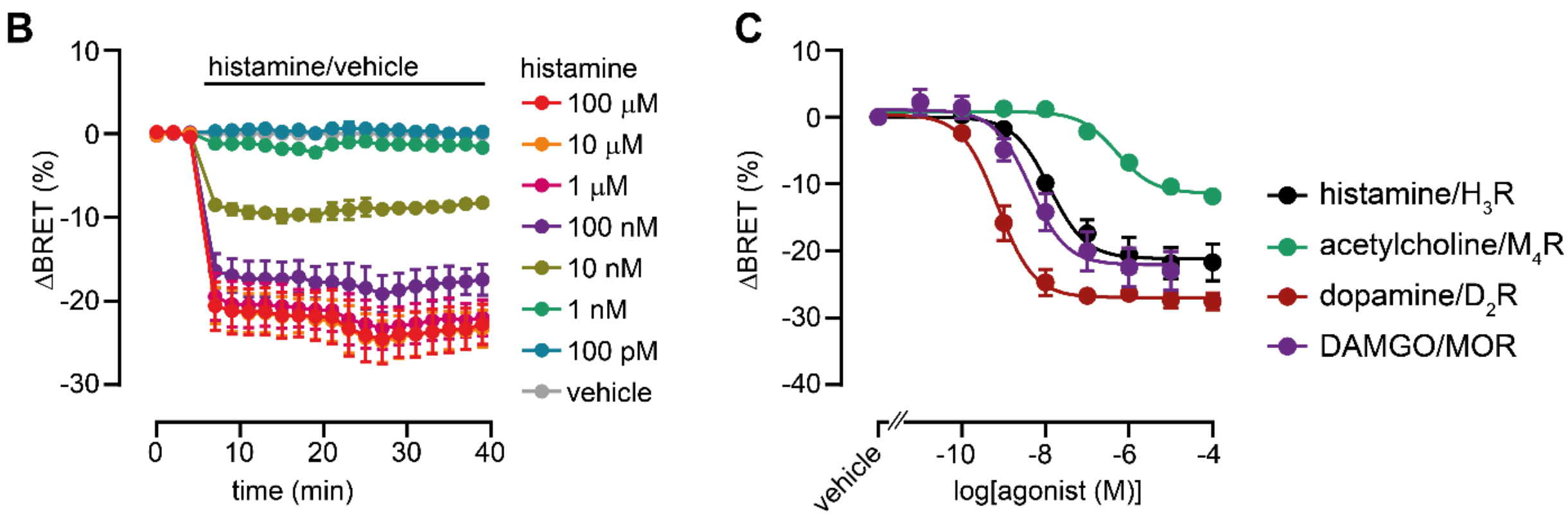

Supplementary Figure 10. Design and validation of Go2-CASE. A) Amino acid sequence of Go2-CASE. **B)** Time course of the histamine-induced BRET changes in HEK-293A cells that have been transiently transfected with the H_3R and Go2-CASE. **C)** Concentration-dependent BRET changes induced by GPCR agonists in HEK-293A cells that have been transiently transfected with the indicated GPCR and Go2-CASE.Data show mean ± SEM of three independent experiments.

Supplementary Figure 11

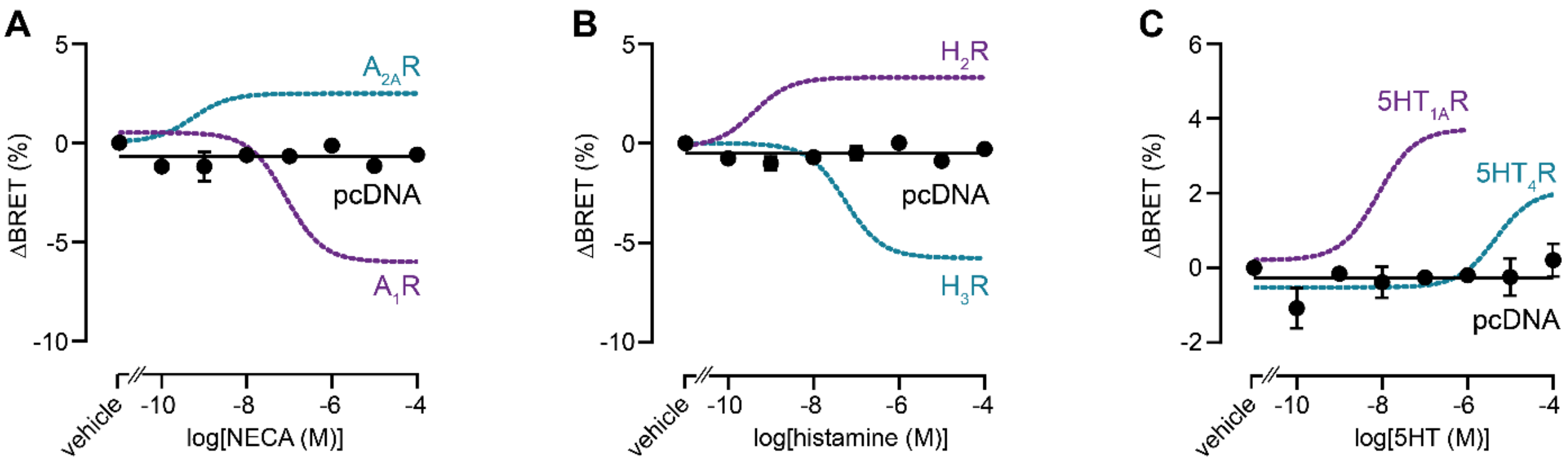

Supplementary Figure 11. G_{gust}-CASE response to endogenous receptor stimulation. Concentration-dependent BRET changes induced by NECA (A), histamine (B) and serotonin (C) in HEK-293A cells that have been transiently transfected with Ggust-CASE and pcDNA. For comparison, the concentration-response curves of the same ligands induced in cells overexpressing the indicated receptors were implemented from Fig. 3. Data show mean ± SEM of three independent experiments.

Supplementary Figure 12

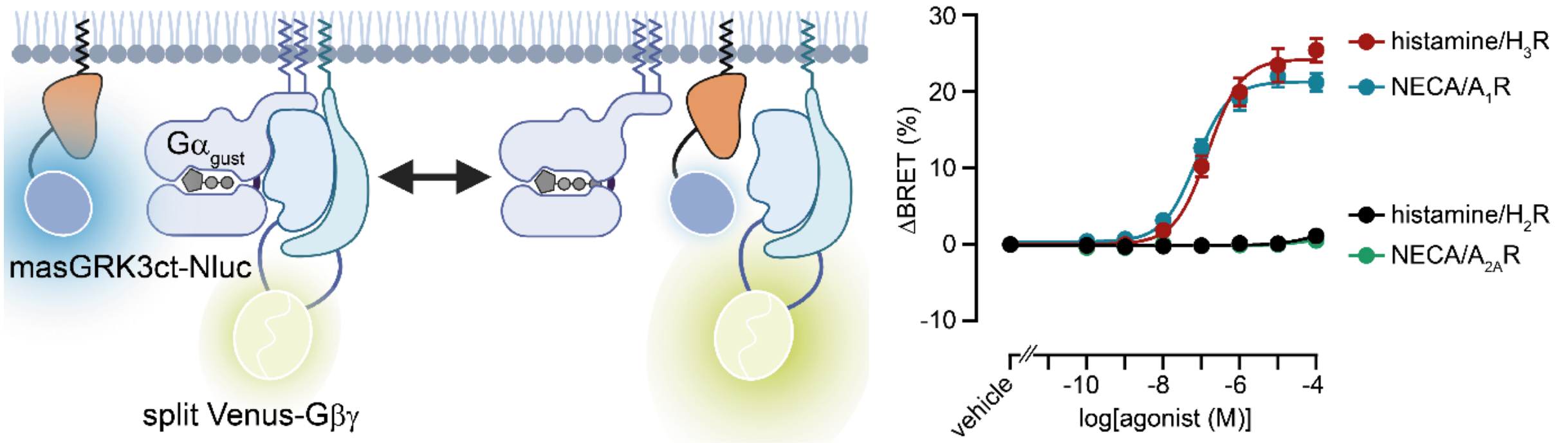

Supplementary Figure 12. GPCR-mediated Gβγ release from G_{gust} heterotrimers. Intermolecular BRET assay scheme based on the interaction of Nluc-tagged masGRK3ct with split Venus-Gβγ upon G_{gust} heterotrimer dissociation and GPCR agonist-induced BRET changes measured in transiently transfected HEK-293A cells devoid of endogenous Gα proteins. Data show mean ± SEM of three independent experiments.

Supplementary Figure 13

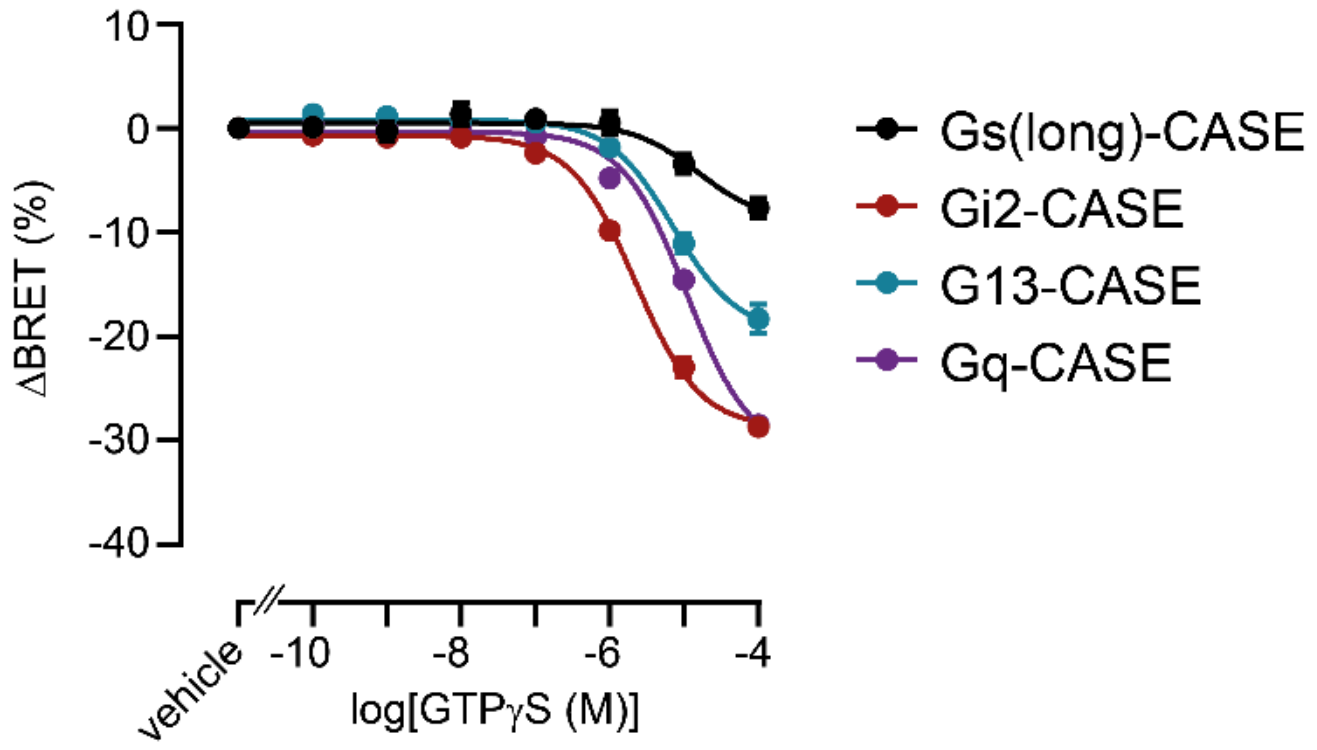

Supplementary Figure 13. GTPγS-induced G-CASE heterotrimer dissociation in permeabilized cells. Concentration-dependent BRET changes induced by GTPγS in permeabilized HEK-293A cells that have been transiently transfected with the indicated G-CASE biosensor. Data show mean ± SEM of three independent experiments.

Supplementary Figure 14

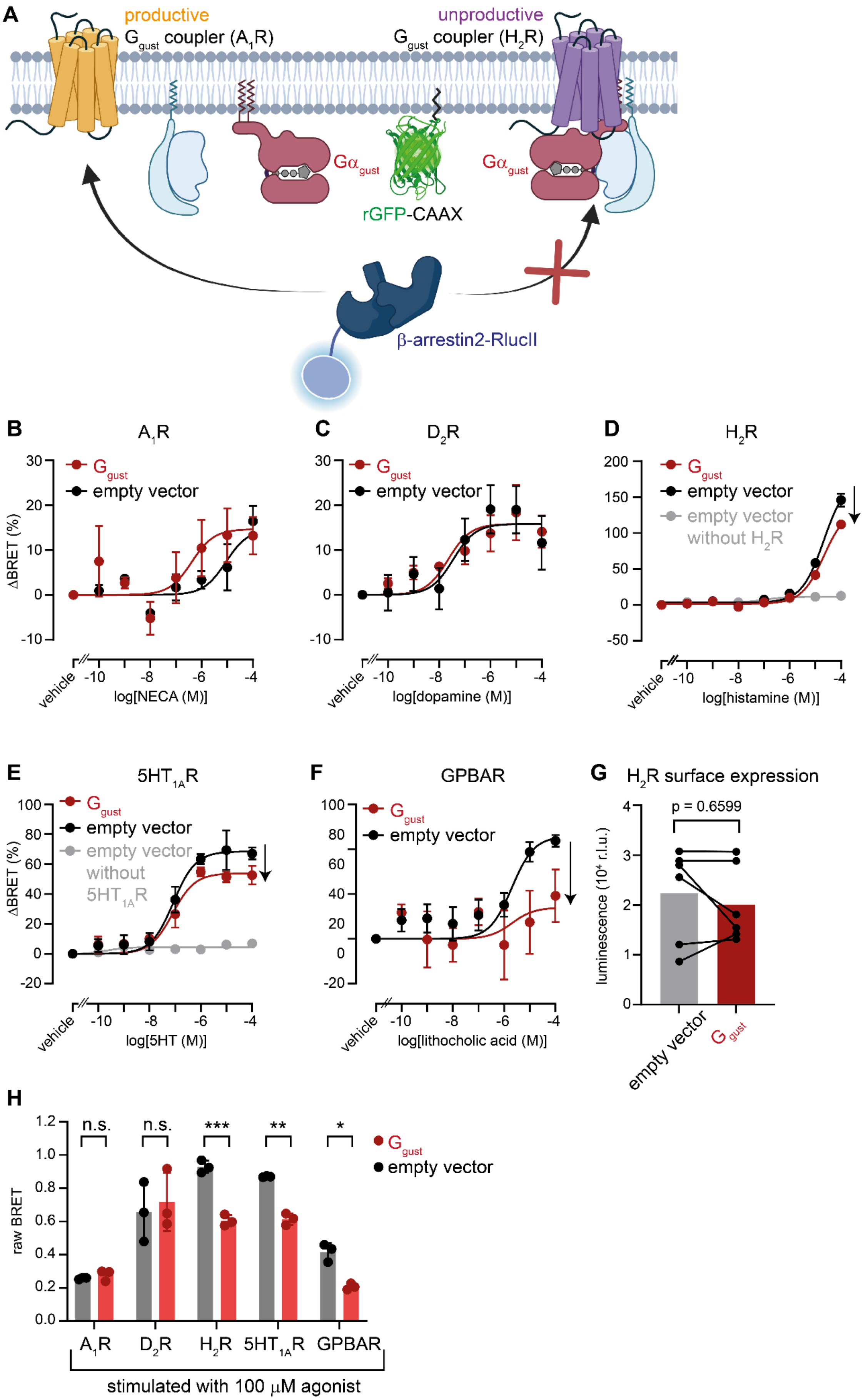

Supplementary Figure 14. Unproductive GPCR-G_{gust} complexes suppress β-arrestin2 recruitment. A) Scheme of the intermolecular BRET assay. **B, C)** GPCR agonist-induced β-arrestin2 recruitment to productive G_{gust}-couplers A_1R and D_2R in the presence and absence of overexpressed G_{gust}. **D-F)** GPCR agonist-induced β-arrestin2 recruitment to unproductive G_{gust}-couplers H_2R, $5HT_{1A}R$ and GPBAR in the presence and absence of overexpressed G_{gust}. In (D) and (E), control experiments without receptor and G_{gust} overexpression have been added to assess the potential interference by endogenously expressed GPCRs on histamine- and serotonin-induced β-arrestin recruitment, respectively. **G)** Luminescence intensity upon HiBiT-LgBiT complementation representing the surface expression levels of H_2R in HEK-293A cells transiently transfected with the HiBiT-H_2R and empty vector (pcDNA3.1(+)) or double dark Ggust-CASE. **H)** Raw BRET values of the data shown in (B-F) for the highest ligand concentration (100 μM). Data show mean ± SEM of three to six independent experiments conducted in transiently transfected HEK-293A cells. Statistical significance in (B-F) was tested using the extra-sum-of-squares F-test and is indicated by the black arrow ($p < 0.05$). Statistical significance in (H) was tested using 2-way ANOVA followed by Sidak's multiple comparison. *$p < 0.05$; ** $p < 0.01$; ***$p < 0.001$.

Supplementary Figure 15

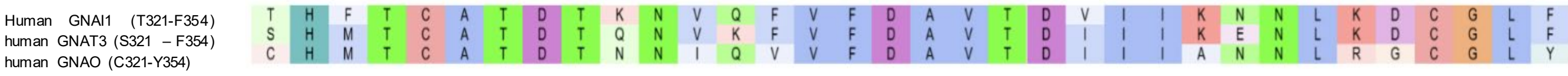

Supplementary Figure 15. Partial alignments of Gα_{i1}, Gα_{gust} and G$\alpha_{o1/2}$. Amino acid sequence alignment of the c-terminal ends of Gα subunits Gα_{i1}, Gα_{gust} and G$\alpha_{o1/2}$.
