# Supplementary material for: Functional diversity of GPCR–Gustducin complexes controls signaling output and suppresses alternative pathways
Source: Nat Commun. 2026 Nov 14;17:11681. doi: 10.1038/s41467-026-79528-z (PMC13769768; doi:10.1038/s41467-026-79528-z)
Supplement: Supplementary file 2 [file 41467_2026_79528_MOESM2_ESM.pdf]

Description of Additional Supplementary Files

File name: Supplementary Data 1
Description: Tissue-level expression of GPCRs in the duodenum and small intestine according to the human protein atlas.

File name: Supplementary Data 2
Description: Single cell coexpression of GPCRs and GNAT3 according to the human protein atlas.
