# Supplementary material for: Functional diversity of GPCR–Gustducin complexes controls signaling output and suppresses alternative pathways
Source: Nat Commun. 2026 Nov 14;17:11681. doi: 10.1038/s41467-026-79528-z (PMC13769768; doi:10.1038/s41467-026-79528-z)
Supplement: Supplementary file 3 [file 41467_2026_79528_MOESM5_ESM.pdf]

nature portfolio

Corresponding author(s): Dr. Hannes Schihada (hannes.schihada@uni-marburg.de)

Last updated by author(s): Sep 28, 2026

# Reporting Summary

Nature Portfolio wishes to improve the reproducibility of the work that we publish. This form provides structure for consistency and transparency in reporting. For further information on Nature Portfolio policies, see our Editorial Policies and the Editorial Policy Checklist.

## Statistics

For all statistical analyses, confirm that the following items are present in the figure legend, table legend, main text, or Methods section.

| n/a | Confirmed | |
|---|---|---|
| ☐ | ☒ | The exact sample size (*n*) for each experimental group/condition, given as a discrete number and unit of measurement |
| ☐ | ☒ | A statement on whether measurements were taken from distinct samples or whether the same sample was measured repeatedly |
| ☐ | ☒ | The statistical test(s) used AND whether they are one- or two-sided<br>*Only common tests should be described solely by name; describe more complex techniques in the Methods section.* |
| ☒ | ☐ | A description of all covariates tested |
| ☐ | ☒ | A description of any assumptions or corrections, such as tests of normality and adjustment for multiple comparisons |
| ☐ | ☒ | A full description of the statistical parameters including central tendency (e.g. means) or other basic estimates (e.g. regression coefficient) AND variation (e.g. standard deviation) or associated estimates of uncertainty (e.g. confidence intervals) |
| ☐ | ☒ | For null hypothesis testing, the test statistic (e.g. *F*, *t*, *r*) with confidence intervals, effect sizes, degrees of freedom and *P* value noted<br>*Give P values as exact values whenever suitable.* |
| ☒ | ☐ | For Bayesian analysis, information on the choice of priors and Markov chain Monte Carlo settings |
| ☒ | ☐ | For hierarchical and complex designs, identification of the appropriate level for tests and full reporting of outcomes |
| ☒ | ☐ | Estimates of effect sizes (e.g. Cohen's *d*, Pearson's *r*), indicating how they were calculated |

*Our web collection on statistics for biologists contains articles on many of the points above.*

## Software and code

Policy information about availability of computer code

| | |
|---|---|
| Data collection | Plate reader data ws obtained using a PheraStar FSX and ClarioStar Plus plate reader and the MARS software for analysis and raw data export from BMG Labtech. |
| Data analysis | The following softwares were used for raw data analysis: Microsoft Office Excel (Version 2603), GraphPad Prism 10. No new code was generated. |

For manuscripts utilizing custom algorithms or software that are central to the research but not yet described in published literature, software must be made available to editors and reviewers. We strongly encourage code deposition in a community repository (e.g. GitHub). See the Nature Portfolio guidelines for submitting code & software for further information.

## Data

Policy information about availability of data

All manuscripts must include a data availability statement. This statement should provide the following information, where applicable:

- Accession codes, unique identifiers, or web links for publicly available datasets
- A description of any restrictions on data availability
- For clinical datasets or third party data, please ensure that the statement adheres to our policy

All data generated or analyzed during this study are provided in the supplementary information files and source data files.

## Research involving human participants, their data, or biological material

Policy information about studies with human participants or human data. See also policy information about sex, gender (identity/presentation), and sexual orientation and race, ethnicity and racism.

| | |
|---|---|
| Reporting on sex and gender | N/A |
| Reporting on race, ethnicity, or other socially relevant groupings | N/A |
| Population characteristics | N/A |
| Recruitment | N/A |
| Ethics oversight | N/A |

Note that full information on the approval of the study protocol must also be provided in the manuscript.

# Field-specific reporting

Please select the one below that is the best fit for your research. If you are not sure, read the appropriate sections before making your selection.

☒ Life sciences ☐ Behavioural & social sciences ☐ Ecological, evolutionary & environmental sciences

For a reference copy of the document with all sections, see nature.com/documents/nr-reporting-summary-flat.pdf

# Life sciences study design

All studies must disclose on these points even when the disclosure is negative.

| | |
|---|---|
| Sample size | All experiments were performed as three or more (as indicated) independent experiments with independent transfections. |
| Data exclusions | No datapoints were excluded. |
| Replication | All experiments could be reproduced in independent biological replicates. |
| Randomization | N/A |
| Blinding | N/A |

# Reporting for specific materials, systems and methods

We require information from authors about some types of materials, experimental systems and methods used in many studies. Here, indicate whether each material, system or method listed is relevant to your study. If you are not sure if a list item applies to your research, read the appropriate section before selecting a response.

## Materials & experimental systems

| n/a | Involved in the study | |
|---|---|---|
| ☒ | ☐ | Antibodies |
| ☐ | ☒ | Eukaryotic cell lines |
| ☒ | ☐ | Palaeontology and archaeology |
| ☒ | ☐ | Animals and other organisms |
| ☒ | ☐ | Clinical data |
| ☒ | ☐ | Dual use research of concern |
| ☒ | ☐ | Plants |

## Methods

| n/a | Involved in the study | |
|---|---|---|
| ☒ | ☐ | ChIP-seq |
| ☒ | ☐ | Flow cytometry |
| ☒ | ☐ | MRI-based neuroimaging |

## Eukaryotic cell lines

Policy information about cell lines and Sex and Gender in Research

| | |
|---|---|
| Cell line source(s) | HEK-293A cells were obtained from ThermoFisher Scientific (catalog number R70507). HEK-293A cells devoid of endogenous Galpha subunit expression were described previously (Y. Hisano, M. Kono, A. Cartier, E. Engelbrecht, K. Kano, K. Kawakami, Y. Xiong, W. Piao, S. Galvani, K. Yanagida, A. Kuo, Y. Ono, S. Ishida, J. Aoki, R.L. Proia, J.S. Bromberg, A. Inoue, T. Hla, Lysolipid |

| | |
|---|---|
| | receptor cross-talk regulates lymphatic endothelial junctions in lymph nodes, J Exp Med. 216 (2019) 1582–1598. https://doi.org/10.1084/JEM.20181895) and kindly provided by Prof. Asuka Inoue (Kyoto University, Tohoku Universiy, Japan) |
| Authentication | None of the cell lines used were authenticated further by the authors. |
| Mycoplasma contamination | All cell lines used were regularly checked for mycoplasma infection using a color reaction-based commercial test kit and were found to be negative. |
| Commonly misidentified lines (See ICLAC register) | We did not use commonly misidentified cell lines. |

## Plants

| | |
|---|---|
| Seed stocks | N/A |
| Novel plant genotypes | N/A |
| Authentication | N/A |
