# Supplementary material for: Functional diversity of GPCR–Gustducin complexes controls signaling output and suppresses alternative pathways
Source: Nat Commun. 2026 Nov 14;17:11681. doi: 10.1038/s41467-026-79528-z (PMC13769768; doi:10.1038/s41467-026-79528-z)
Supplement: Supplementary file 4 [file 41467_2026_79528_MOESM6_ESM.pdf]

nature portfolio

Peer Review File

Functional diversity of GPCR-Gustducin complexes controls signaling output and suppresses alternative pathways

Corresponding Author: Dr Hannes Schihada

This file contains all reviewer reports in order by version, followed by all author rebuttals in order by version.

Version 0:

Reviewer comments:

Reviewer #1

(Remarks to the Author)
I greatly enjoyed reading this manuscript. This refreshing and elegant study from the Schihada lab sheds light on the coupling properties of gustducin, a heterotrimeric Gα subunit that remains relatively understudied despite its important role in sensory taste cells. The study is timely for two reasons: (1) gustducin is now known to be expressed beyond taste cells, in additional cellular contexts; and (2) several Gα proteins have recently been shown, under certain conditions, to engage in non-productive coupling behaviour. Although difficult to capture in physiologically relevant cellular models, this type of coupling has emerged as an additional mechanism by which GPCR signalling may be shaped through interference with productive downstream receptor signalling.

Overall, the work presented is rigorous, methodologically sound, and largely supports the authors' conclusions and claims. However, a few additional experiments, outlined below, would further strengthen the manuscript and enhance the robustness of the conclusions. Please find my comments and suggestions below:

• For TRUPATH, the heterotrimeric BRET2 biosensor used to monitor gustducin activation employs Gβ3 with Gγ1. What was the rationale for using the Gβ3/Gγ9 combination for Ggust-CASE instead of Gβ3/Gγ1 (as in the TRUPATH version)? This rationale should be stated in the Results section introducing Ggust-CASE.

• Similarly, the rationale for using cpVenus instead of Venus should also be provided.

• Figure 1C-D: Why was a Gi-coupled receptor used here to assess the functionality of Ggust-CASE rather than TAS2Rs or TAS1Rs?

• Figure S3E-F: AC5 appears to be a suboptimal effector for Gi or Gs, given the very small signal-to-noise ratio observed. Why not use Rap1GAP for Gi, as described in the effector membrane translocation assay (EMTA; DOI: 10.7554/eLife.74101)? For Gs, why not use the KB1691 peptide reported to bind specifically to GTP-bound Gs (DOI: 10.1016/j.cell.2024.01.028)?

• In the section entitled "Productive and unproductive GPCR-Ggust complexes revealed by nucleotide sensitivity experiments", the sentence "The C-terminally Nluc-tagged receptors were co-expressed the Venus-labeled Gβγ dimer and wildtype Gαgust in HEK-293 cells lacking endogenous Gα subtypes [41]." is missing the word "with" between "co-expressed" and "the Venus-labeled".

• Figure 7A-B: The impact of gustducin on A1R-mediated Gq activation (unaffected) and H2R-mediated Gs activation (impaired) was examined. Did the authors also verify whether H3R- and BK2R-mediated signalling are unaffected, and whether 5HT1AR- and A2AR-mediated signalling are similarly impaired? If such data exist, they should be added to Figure 7. If not, these experiments would be important to generalise the concept across a broader range of receptors engaging gustducin. The same comment applies to Figure S8 regarding β-arrestin recruitment to the plasma membrane.

• Figure S8 (Comment 1): The authors show that cell-surface expression of H2R is equivalent in cells expressing empty vector or double-dark Ggust-CASE, which is an appropriate control. However, did the authors also verify whether transient expression levels of rGFP-CAAX and β-arrestin2-RlucII (and thus the acceptor/donor ratio) remain comparable between

empty vector- and double-dark Ggust-CASE-expressing cells? In my experience, enhanced bystander BRET (ebBRET) can be challenging, as ΔBRET values often increase linearly with the acceptor/donor ratio and rarely reach saturation, unlike NanoBRET, BRET1, or BRET2. Consequently, with rGFP and RlucII, ΔBRET values are highly sensitive to variations in acceptor/donor ratio. Please therefore report the acceptor/donor ratios at maximal agonist concentration for both empty vector and double-dark Ggust-CASE conditions for each receptor tested, to exclude artefactual interpretations.

• Figure S8 (Comment 2): In panel B, the dose-response curve obtained with double-dark Ggust-CASE does not appear to be fitted with basal constrained to zero. In addition, an unpaired Student's t-test is not appropriate for comparing agonist-induced maximal efficacy. Please instead use an extra sum-of-squares F-test, as performed in Figure 3.

Reviewer #2

(Remarks to the Author)
Arce et al. report new BRET biosensors for gustducin activation and test whether non-taste GPCRs can engage gustducin. The key finding is that some GPCRs couple productively to gustducin, while others are unproductive gustducin-couplers.

Noteworthy results
The authors identify GPCRs co-expressed with GNAT3 in different tissues and test 24 selected in transfected HEK293A cells. In this system, they define two functional classes of GPCR-gustducin interactions: productive (H3R, A1R, BK2R, and D2R, µOR, APLNR) and unproductive couplers (A2AR, H2R, 5HT1AR). I believe, a third group would be non-interactors. One mechanistic claim is that unproductive GPCR-gustducin complexes suppress alternative G protein signaling pathways, but the supporting data are very limited.

Significance and originality
The findings are likely significant, specifically for GPCR chemosensing and biased signaling. I believe the significance could be increased if not only a selection of receptors were validated, but also a broader panel.
The phenomenon itself is likely not Ggust-specific, but also relevant to other GPCR-G protein interactions. It would increase the study's significance if such a hypothesis was tested for selected receptors.
As the study builds on previous results showing Ggust's involvement in pancreatic β cells, I would expect at least a few of the receptors reported in the cited studies to be included in the proper validation of the Ggust activation sensors, such as taste receptors, T1Rs, and T2Rs (reference 44) and/or GPR40, GPR41, GPR43, GPR119, GPR120, TGR5 (reference 45). This would enhance the study's significance by experimentally relating to previous findings.

Do the data support the conclusions?
Mostly, the biosensor data support the notion that GPCRs can elicit distinct gustducin conformational/signaling outcomes, although the receptor selection in the validating assays should not be narrowed down. The PDE6 assays should be performed for the full set of receptors shown in Figure 3A, as both productive and non-coupling should be demonstrated consistently for both systems.
Further, in vivo physiological relevance remains to be tested, but is surely out of the scope of the present manuscript. However, this specifically means that additional evidence is needed to support broader claims about physiology, as the current evidence is largely from transiently transfected HEK293A cells rather than native intestinal, taste, pancreatic, or neuronal cells. The claim that these mechanisms regulate nutrient sensing or hormone secretion in vivo is plausible but not demonstrated.

Flaws or revisions needed
The main limitation is biological context: overexpression in HEK293A cells can alter receptor/G-protein stoichiometry, basal activity, coupling selectivity, and BRET geometry. This does not prohibit publication, but it requires careful wording and ideally validation in native or more physiologically relevant cells. According to the expression atlas, e.g., SW48 cells express high levels of GNAT3 and presumably also at least one of the chosen GPCRs, which could potentially serve as a preliminary model cell line.
The interpretation of the increased BRET as evidence of unproductive coupling mechanisms is reasonable but not conclusively proven. Again, alternative approaches using endogenously GNAT3-expressing cells in combination with knockdown strategies and/or other assay readouts could strengthen the model.
Further, the switching between several (histamine) receptors across different assay systems is unclear, which is more specifically outlined below.

In Figure 2B, it is not clear why H1R Gαq-Nluc, as "control," is compared to H3R Gαgust-Nluc in Figure 2C. It would make more sense to show in Figure 2B Gi signaling of H3R as control, and in Figure 2D that Gαi-Nluc does not interact with PDE6γ-cpVenus upon H3R activation. As it is now, H1R is always flat, and the comparison between H1R and H3R here is confusing.

It becomes clear from Figure S3 that H1R is a good Gq-coupler, but that H3R Gi2-coupling is as low as its coupling to Ggust. Why was Gi2 chosen and not Gi1 or Gi3? If the robustness of the system is to be established, the data for "standard" coupling should be more convincing, showing robust YFP-AC5 Gi and AC5-YFP-Gs interactions, thereby supporting the negative results for H3R. For H2R, Figure S3F does not support agonist-induced increases in BRET for Gs. If so, I would suggest adapting Figure S3F to look like S3E. It is also not clear why H2R was chosen here. It is only a secondary Gs-coupler. Similarly, TPα is primarily coupled to Gq and not G13.

According to the supplementary Table, it should be 201, not 202, GPCRs co-expressed with GNAT3.

Further, H3R is not co-expressed with GNAT3 in GIT/accessory tissues, brain, or adipose tissue, but was chosen as a productive coupler. I don't see the rationale for choosing H3R as a prototypical Gi/o-coupled GPCR to validate Ggust initially. It rather seems like a coincidence that the most productively coupling Gi-coupled GPCR was chosen.

I agree with the concentration-dependence of the deltaBRET signal, but think the time-dependence is a bit far-fetched, as it is neither interpreted nor does the signal change very much over time. Real kinetics analyses would need to be conducted in a much shorter time frame.

"Our selection included GPCRs with G protein coupling profiles ranging from very selective (e.g., V1BR, P2YR1) to very promiscuous (e.g., BK2R, H1R) and covered all four major Gα families." I do not find this reasoning for selecting the GPCRs very convincing. Although the cited publication, Hauser et al. 2022, shows that P2RY1 would very selectively couple to G15, several publications report Gq coupling for P2YR1, which is also inhibited by the Gq/11-selective inhibitor YM-254890. Similarly, in the cited publication, how is V1BR an example of a very selective G protein-coupling profile, whereas ADRA1D is, for example, equally selective?

Wouldn't it, in turn, be more reasonable to argue that GPCRs that are indeed co-expressed with GNAT3 from co-expression analyses were chosen, specifically those that show the highest percentage of co-expression?

Regarding co-expression, I believe the reader would benefit from knowing the percentage of cells in which GNAT3 co-expresses with receptors in a selected cell type. A statement about a percentage range would surely be helpful, enabling the reader to know that it is not 20-100% of cells co-expressing both, but rather much lower numbers to put the findings in context. This would not affect the significance of the findings but would make it clearer for follow-up studies when designing their experiments.

Similar to H2R, I would suggest including GCASE controls for the primary and secondary signal transduction pathways for the other 14 receptors tested to ensure they function in the assay system.

As G-CASE sensors have been reported before, the design itself is not new.
However, two additional GCASE sensors are mentioned in the text, G11-CASE and Go2-CASE. In Figure S5, H2R shows no response with G11-CASE and a very low response with Go2-CASE. Figure S7 shows a histamine-dependent G11-CASE activation for H1R.
For G11, several of the selected receptors make good candidates to properly validate the G11 CASE sensor, as they are in Hauser et al 2022 depicted as G11-couplers, such as A1R, A2AR, P2YR11, M1R, M2R, M3R, HT2R (which one a, b or c?), V1B, BK1, BK2.
The same is true for GoB and A1R, M1R, M3R, M4R, HT1R (which one a, b, d, e or f?) HT2R (which one a, b or c?), V1B, BK1, BK2, the other Histamine receptor, D2R, D5, CB1, APLNR, etc.
I suggest including a proper validation of these two sensors by showing data for more than two selected GPCRs. The rationale for choosing these two is not clear.

In Figure 3B, some receptor responses are difficult to distinguish, such as MT2R and A1R. I also think the figure would benefit from rearrangement, not grouping by primary G protein-coupling, but rather, for example, by ligand type and receptor family (all 4 HTR in one graph, P2Y with adenosine receptors, histamine receptors with dopamine receptors).
Are there other examples in which receptor activation actually causes an increase in GCASE-BRET responses? It seems likely that this is not a Ggust-specific phenomenon.

The low potency of GTP S should not be stated as pEC50 but rather as the actual value to help the reader with the direct comparison (~30-60 fold lower potency).

Although the GTPγS-induced decrease in BRET may indicate that Ggust is partially active, the GTPγS concentrations required to observe this decrease, as stated by the authors, were very high. Again, here I would speculate that this is not a Ggust CASE-specific phenomenon.

Furthermore, HEK293 cells endogenously express several GPCRs that are relevant to the observed results. I think that an analogous analysis with other GCASE sensors that show or do not show basal activity using these GTP assays would be valuable, potentially also to compare the different sensors and draw broader conclusions.

In Figure 3, BK2R is a productive Ggust-coupler like A1R and D2R and H3R (Figure 1C), but in Figure 6B, presumably, "biphasic behavior". Because the concentration of each agonist is unspecified and a single concentration is used, the data do not adequately support the point.

The authors state that GPCR-Ggust impacts other G protein-GPCR couplings and use a double dark Ggust-CASE.

What is not clear here is why A1R was chosen to analyze for Gq, while it primarily couples to Gi, and why H2R, a primarily Gq-coupled receptor, was chosen to test Gs (long). A1R's secondary signaling mechanisms are Gs- and Gq-coupling. However, to make the point that Ggust is the alternative pathway to the respective secondary transduction mechanisms, similar experiments should be performed testing the GCASE sensor for the primary signal transduction mechanism.

Although Figure 7C effectively illustrates the broader validity of the results, the experimental evidence is insufficient at present. BK2R, D2R, D2R, and APLNR as productive couplers could be included as additional examples supporting the conclusion. Similarly, for unproductive couplers A2AR, 5HT1AR, 5HT4R, and M4R.

The authors controlled for H2R cell surface expression and observed no significant differences, but saw reduced arrestin translocation to the plasma membrane with Ggust-CASE double dark. Figure S8D suggests that 2 of the 6 experiments showed the opposite direction, and that if these were excluded, H2R cell surface expression would be decreased. It would be helpful if there were lines between the matching experiments, or if another, more robust assay system was used to support this conclusion.
HEK293A cells endogenously express GPCRs that may be activated by the chosen ligands, such as 5-HT receptor subtypes. Can interferences with the assay readout be excluded?

Methodology
The methodology is generally sound for live-cell GPCR signaling pharmacology with NanoLuc/cpVenus BRET sensors. The sensors are likely to be valuable to the broader community, although further validation within the present manuscript is required. The inclusion of dark/double-dark variants strengthens the results, as does the GTPγS/GDPβS nucleotide manipulation.
However, there is a need for broader validation of the new sensors G11 and GoA, specifically to compare them to the Ggust-CASE signals. Similarly, showing that the primary and, if applicable, secondary G protein signal transduction pathways can be reliably detected with the respective (for each GPCR) relevant GCASE sensor is mandatory to ensure the system itself is robust. As it stands, the comparison with other G-protein biosensors is insufficient.
The work meets expected standards for a mechanistic cell-signaling study, but not yet for strong physiological claims.

Reproducibility
The methods provide details on reagents, constructs, cell handling, permeabilization, nucleotide treatments, BRET assays. However, it is not clear which datapoint(s) was/were used from the time-curve(s), such as 2C, to generate curves, such as shown in 2D or 3B. Since deltaBRET is still reported as a percentage, I assume the AUC was not interpolated. The statement in the method section, "time points with saturating response of all concentrations tested were selected," is insufficiently clear and suggests that the choice differed between receptors.

Reviewer #3

(Remarks to the Author)
In this manuscript Jaime Arce et al. develop biosensors for the detection of gustducin coupling and activation and describe the ability of this G protein to interact with non-taste GPCRs to form what they call productive or unproductive complexes. By building on the TRUPATH and G-CASE toolbox, the authors have generated Ggust-CASE, a biosensor that detects the dissociation of the Ggust heterotrimer. Additionally, they have developed a BRET approach that monitors the proximity between Galphagust and PDE6gamma, as an orthogonal measure of G protein activation. These two sensors are potentially of interest to the wide GPCR community. In the second, more conceptual part of this manuscript, the authors describe a mechanism whereby coupling of some GPCRs to Ggust can result in two outcomes: productive coupling and G protein heterotrimer dissociation or unproductive coupling and sequestration of GPCRs from coupling to other G proteins.
Overall, this is an interesting study with potential technical and conceptual advances. However, in the present form, the manuscript lacks data to support the generalisability of these advances, as claimed by the authors. Thus, with the acknowledgement that this work presents a potentially useful tool for GPCR research, there are a few considerations that limit our enthusiasm for this manuscript in the current form.

General comments:
1. While the focus of the study is to describe the activation of and interactions between Ggust and non-gustatory receptors, it is surprising that the authors did not include Ggust-CASE experiments with any tastant-activated GPCRs. These experiments would confirm that the newly created sensor is suited to also pick up signalling responses of receptors that are primarily coupled to Ggust.

2. The data displayed in Figure 3B appears to be one of the main findings of this study. The panel of used receptors is nicely justified by the tissue and single-cell expression data analysis, however, the authors could find a way to better showcase the behaviours of these different GPCRs, in addition to concentration-response curves. For example, a clustering heatmap of those responses would allow for the evaluation of all tested GPCRs and aid in the classification of receptors based on these.

3. In line with the previous comment, they authors should show that for those GPCRs with productive coupling, this coupling sensitive to antagonist treatment.

4. One of the main conclusions of the study relies on the measurements in Figure 7 and S8, which are only performed at two receptors: the A1R and H2R. While the G protein activation data in Figure 7A and B is convincing, the data in S8 does not show robust effects. In addition, to support the proposed mechanism, these (G protein activation and arrestin bystander recruitment experiments) should also be performed for the entire set of receptors featured in Figure 3, or at least a more substantial panel of receptors. This will allow for a comprehensive assessment of the proposed sequestration mechanism.

5. Ggust belongs to the Gi/o/z family, and its description of coupling outside the taste receptor family is interesting. However, there are a few characterisation steps of the sensor and descriptions that are missing:
a. Ggust transduces signal via AC inhibition. Does the "productive" coupling of Ggust by non-taste GPCRs decrease cAMP levels?
b. Is the sensor and the potential cAMP response sensitive to PTx? It seems a missed opportunity to use PTx to provide further insight into the distinction of productive/unproductive coupling

6. In line with the comment above, sequestration and "unproductivity" would be more relevant if tested for downstream, second messenger assays.

7. Finally, there is no consideration of the GDP/GTP exchange rate or GTPase activity of gustducin? Could differences among other G protein isoforms explain some of the data? While, if different this could be experimentally validated, the authors should at least discuss this possibility.

Specific/methodological comments:
8. The kinetic resolution of data of Figure 1C and similar could use some kinetic curve fitting.

9. Raw baseline data is not presented in any figure, this is particularly important for GDP/GTPgS experiments (but also in the different GPCR coupling description) and their interpretation. The authors should provide and discuss this.

Minor comments:
Abstract:
• "…characterization of Ggust activity by non-taste GPCRs." Should be: "…characterization of Ggust activity induced by non-taste GPCRs."

• Throughout the manuscript, the authors use a mixture of hyphens and EN-dashes to describe protein–protein interactions e.g. "…unproductive GPCR-G protein complexes,…" Here, the EN-dash should be used instead of a hyphen: "…unproductive GPCR–G protein complexes,…" and this should be consistent throughout the manuscript.

Methods:
• Clarify how deltaBRET was calculated (from AUC vs at a particular time point).

Introduction:
• "Here, Gαgust couples taste receptors to glucagon-like peptide 1 (GLP-1) secretion, ghrelin release and expression of sodium-dependent glucose transporter isoform 1 (SGLT1)[11–13]." We suggest to change "couples" to "links" to avoid confusion about G protein-coupling.

• GPCR-specific conformational responses in the Ggust heterotrimer: Instead of the possessive "Ggust's", we recommend rephrasing to "the primary physiological function of Ggust…" to improve readability.

Discussion:
• "…complements recent reports of unproductive GPCR–G protein complexes and raises intriguing question regarding the physiological consequences…" should be "…raising intriguing questions..."

Reviewer #4

(Remarks to the Author)
I co-reviewed this manuscript with one of the reviewers who provided the listed reports. This is part of the Nature Communications initiative to facilitate training in peer review and to provide appropriate recognition for Early Career Researchers who co-review manuscripts.

Version 1:

Reviewer comments:

Reviewer #1

(Remarks to the Author)
The authors have addressed my previous concerns carefully and constructively, and the revised manuscript has been substantially improved. I appreciate the authors' thorough responses to my previous comments. I have only a few minor concerns remaining:

• Line 183: The authors state that they confirmed that all 26 receptors were able to activate sensors of the primary and/or secondary G proteins upon co-expression and agonist stimulation, referring to Figure S6. I am not sure that "activation" is the appropriate term here, as the ΔBRET values observed for BK1R and 5-HT2BR with their primary G protein, Gq, are positive rather than negative. With the G-CASE sensor, a positive ΔBRET would appear to indicate non-productive coupling rather than G protein activation. The wording should therefore be revised to distinguish receptor-G protein engagement from productive G protein activation.

• Figure 3B: Ggust is a member of the Gi/o family. It is therefore expected that receptors in cluster B, which are productive Ggust couplers, predominantly activate members of the Gi/o family. However, BK2R appears to represent an interesting exception. According to Figure S6, BK2R primarily couples to Gq and secondarily to G13, yet it is classified as a productive Ggust coupler. Do the authors have an explanation for why BK2R does not appear to activate the canonical Gi/o proteins tested, while nevertheless showing productive coupling to Ggust? At minimum, this apparent exception should be acknowledged in the text accompanying Figure 3B.

• Line 190: The authors state that GPCR activation induced not only decreases in Ggust-CASE BRET, which were sensitive to pre-incubation with GPCR agonists, but also positive BRET changes. I believe the authors mean antagonists rather than agonists here. Please correct.

• Figure S14: The newly added panel H of Figure S14 is not described in the Results section. Please ensure that this panel and its main finding are appropriately introduced and discussed in the Results.

• Figure S15: Similarly, I could not identify a corresponding description of Figure S15 in the Results section. Since this supplementary figure is subsequently referred to in the Discussion (line 414), the relevant results should first be presented in the Results section.

Reviewer #2

(Remarks to the Author)
The authors substantially expanded the study's experimental scope and addressed my previous concerns. They extended the set of non-tastant GPCRs used to assess productive and unproductive coupling and added further examples of unproductive GPCR-G protein complexes. They also included Tas2R14 and TGR5 in their evaluation, with the latter providing a convincing example of unproductive Ggust coupling. Together, these additions broaden the relevance of the findings and strengthen the overall significance of the study.
I agree with the authors that it is not necessary to examine all receptors expressed in pancreatic β cells, although I disagree that all GPRs lack suitable tool ligands.
The authors also considerably strengthened the experimental validation of their approach. They expanded the PDE6γ validation from 5 to 16 receptors and added an orthogonal Gβγ-release assay. In addition, G11-CASE and Go2-CASE were validated with further receptors, and primary and secondary G-protein signaling was examined across the receptor panel. Further, Figure 7 was expanded, and additional controls were included to address potential interference from endogenous receptors.
In summary, I believe these complementary data strengthen the manuscript's conclusions. They further support grouping GPCRs into productive and unproductive couplers. The authors have addressed my concerns, and I support publication of the revised manuscript. I look forward to future studies in cells endogenously expressing GNAT3 and the relevant GPCRs to establish the physiological relevance of these different coupling behaviors.

Minor comments
Figure S6: Was the two-way ANOVA followed by Fisher’s LSD multiple-comparisons test performed against a normalized value of zero without associated error? If so, I am not sure that this comparison is appropriate. I also do not think it is necessary to plot a vehicle (VH) value of zero for each receptor. More generally, I do not see a clear need for statistical testing in this analysis.
“Flufenamic acid” appears as “lufenamic acid” at one point before Figure 1.
The Methods section refers to KB1694, whereas the Results section refers to KB1691. Please verify the correct identifier and use it consistently.

Reviewer #3

(Remarks to the Author)
The authors have addressed our concerns appropriatedly. Just note a couple of typos:
- Page 3 end of the first paragraph: name of TasR ligand is misspelled.
- Page 6 :"...which were sensitive to pre-incubation with GPCR agonists (Fig. S7),..." presumably the authors mean antagonists?

Reviewer #4

(Remarks to the Author)
I co-reviewed this manuscript with one of the reviewers who provided the listed reports. This is part of the Nature Communications initiative to facilitate training in peer review and to provide appropriate recognition for Early Career Researchers who co-review manuscripts.

Open Access This Peer Review File is licensed under a Creative Commons Attribution 4.0 International License, which permits use, sharing, adaptation, distribution and reproduction in any medium or format, as long as you give appropriate credit to the original author(s) and the source, provide a link to the Creative Commons license, and indicate if changes were made.
In cases where reviewers are anonymous, credit should be given to 'Anonymous Referee' and the source.
The images or other third party material in this Peer Review File are included in the article's Creative Commons license, unless indicated otherwise in a credit line to the material. If material is not included in the article's Creative Commons license and your intended use is not permitted by statutory regulation or exceeds the permitted use, you will need to obtain permission directly from the copyright holder.
To view a copy of this license, visit https://creativecommons.org/licenses/by/4.0/

Point-by-point response to reviewer comments on

"Functional diversity of GPCR-Gustducin complexes controls signaling output and suppresses alternative pathways"

Reviewer #1 (Remarks to the Author):

I greatly enjoyed reading this manuscript. This refreshing and elegant study from the Schihada lab sheds light on the coupling properties of gustducin, a heterotrimeric Gα subunit that remains relatively understudied despite its important role in sensory taste cells. The study is timely for two reasons: (1) gustducin is now known to be expressed beyond taste cells, in additional cellular contexts; and (2) several Gα proteins have recently been shown, under certain conditions, to engage in non-productive coupling behaviour. Although difficult to capture in physiologically relevant cellular models, this type of coupling has emerged as an additional mechanism by which GPCR signalling may be shaped through interference with productive downstream receptor signalling.

Overall, the work presented is rigorous, methodologically sound, and largely supports the authors' conclusions and claims. However, a few additional experiments, outlined below, would further strengthen the manuscript and enhance the robustness of the conclusions. Please find my comments and suggestions below:

For TRUPATH, the heterotrimeric BRET2 biosensor used to monitor gustducin activation employs Gβ3 with Gγ1. What was the rationale for using the Gβ3/Gγ9 combination for Ggust-CASE instead of Gβ3/Gγ1 (as in the TRUPATH version)? This rationale should be stated in the Results section introducing Ggust-CASE.

We thank reviewer #1 for evaluating our manuscript and for the positive feedback received.

The combination β3γ9 was used for the design of Ggust-CASE because this dimer has been proven to be a valuable pair for some of our previous G-CASEs, including Go1, Gq, and G13 (c.f.; DOI: 10.1126/scisignal.abf1653). Furthermore, differences in the response of TRUPATH sensors comprising β3γ1 vs. β3γ9 are very small, presumably because Gγ1 and Gγ9 are highly similar and belong to the same subclass of Gγ proteins.

This information is now provided with our revised manuscript.

Similarly, the rationale for using cpVenus instead of Venus should also be provided.

The yellow fluorescent protein cpVenus has served as our standard BRET acceptor in all previous G-CASE sensors that we developed (c.f.; DOI: 10.1126/scisignal.abf1653 and DOI: 10.1016/j.jbc.2022.102328) because it has been shown in earlier studies to enhance the dynamic range of resonance-energy-transfer-based sensors (DOI: 10.1016/j.chembiol.2013.12.012; DOI: 10.1073/pnas.0400417101; DOI: 10.1016/j.bbrc.2006.07.136).

We added this rationale to our results section as suggested by the reviewer.

Figure 1C-D: Why was a Gi-coupled receptor used here to assess the functionality of Ggust-CASE rather than TAS2Rs or TAS1Rs?

The Gi/o coupled H_3R was used for practical reasons; specifically, because we didn't have plasmids coding for any of the taste receptors in our lab at the time. Additionally, the Roth lab also used a prototypical Gi/o coupled, non-taste GPCR for validating their Ggust TRUPATH sensor back in 2020.

Yet, we understand the relevance of validating a new sensor for gustducin with one of its primary GPCRs and obtained a plasmid for the expression of the human taste receptor Tas2R14 in HEK293 cells. The 3D structure of this receptor has previously been solved in complex with the agonist flufenamic acid and a chimeric mini G protein of Ggust/s.

Our new experiments showed that flufenamic acid induced an even more pronounced, concentration-dependent decrease in Ggust-CASE BRET in cells transfected with Tas2R14 compared to histamine-treated H_3R-expressing cells. The concentration-response curve of flufenamic acid has been added to **Fig. 1D**.

Figure S3E-F: AC5 appears to be a suboptimal effector for Gi or Gs, given the very small signal-to-noise ratio observed. Why not use Rap1GAP for Gi, as described in the effector membrane translocation assay (EMTA; DOI: 10.7554/eLife.74101)? For Gs, why not use the KB1691 peptide reported to bind specifically to GTP-bound Gs (DOI: 10.1016/j.cell.2024.01.028)?

We thank the reviewer for this smart suggestion. We obtained plasmids encoding the cpVenus-tagged peptides KB1691 (for $G\alpha_s$) and KB1753 (for $G\alpha_{i/o}$) and performed experiments with Nluc-tagged $G\alpha$ proteins. KB1753 was chosen instead of Rap1GAP because of its smaller size and potentially closer proximity between the BRET labels – and hence potentially higher dynamic range of the assay.
Both peptides exhibited GPCR agonist-induced, concentration-dependent association with their cognate $G\alpha$ proteins but also associated with $G\alpha_{gust}$. KB1691 showed a very low ~ 2% BRET increase with $G\alpha$gust, while KB1753 demonstrated an ~ 10% increase in BRET with $G\alpha_{gust}$ – similar to PDE6γ. However, the potency of the agonist (histamine) was substantially lower in the $G\alpha$gust-KB1753 assay compared to our previous data obtained with the same agonist/receptor combination and $G\alpha_{gust}$-PDE6γ (~ 3 µM vs. ~ 10 nM), indicating that the interaction between $G\alpha_{gust}$ and PDE6γ provides a more sensitive readout for $G\alpha_{gust}$ activity among the different protein-protein-interaction setups we tested.

The concentration response curves obtained with the peptides KB1691 and KB1753 were added to **Fig. S3F and Fig. S3H**.

• In the section entitled "Productive and unproductive GPCR-Ggust complexes revealed by nucleotide sensitivity experiments", the sentence "The C-terminally Nluc-tagged receptors

were co-expressed with the Venus-labeled Gβγ dimer and wildtype Gαgust in HEK-293 cells lacking endogenous Gα subtypes [41].” is missing the word “with” between “co-expressed” and “the Venus-labeled”.

We thank the reviewer for spotting this mistake and have added “with” to the sentence.

Figure 7A-B: The impact of gustducin on A1R-mediated Gq activation (unaffected) and H2R-mediated Gs activation (impaired) was examined. Did the authors also verify whether H3R- and BK2R-mediated signalling are unaffected, and whether 5HT1AR- and A2AR-mediated signalling are similarly impaired? If such data exist, they should be added to Figure 7. If not, these experiments would be important to generalise the concept across a broader range of receptors engaging gustducin. The same comment applies to Figure S8 regarding β-arrestin recruitment to the plasma membrane.

We thank the reviewer for this suggestion. To address a comment from reviewer #2, we also included the GPBAR (G protein-coupled bile acid receptor; a.k.a. TGR5) and an additional subset of GPCR/G protein and GPCR/β-arrestin pairs to examine the effect of G_{gust} overexpression on alternative signaling pathways mediated by productive vs. unproductive G_{gust} couplers. Our selection further included $A_{2A}R$/Gi2-CASE and H_2R/Gq-CASE combinations to compare the impact of G_{gust} coupling on primary vs. secondary GPCR-G protein complexes.

Overall, the new dataset aligns very well with our previous findings. The activation of alternative G proteins by productive gustducin couplers H_3R, A_1R (now with Gi2-CASE), APLNR, and D_2R was insensitive to gustducin overexpression. In contrast, alternative G protein activation by unproductive gustducin couplers $A_{2A}R$, GPBAR, $5HT_{1A}R$, and H_2R (now measured with the secondary G protein Gq) was impaired in the presence of overexpressed Ggust.

Also, with respect to β-arrestin recruitment, the productive G_{gust} couplers D_2R and APLNR were unaffected by G_{gust} overexpression, while gustducin limited recruitment to the unproductive couplers $5HT_{1A}R$ and GPBAR. For the $A_{2A}R$ and H_3R, no arrestin recruitment could be detected even in the absence of gustducin overexpression. The new data have been added as additional panels to **Fig. 7** and Supplementary **Fig. S14**.

Figure S8 (Comment 1): The authors show that cell-surface expression of H2R is equivalent in cells expressing an empty vector or double-dark Ggust-CASE, which is an appropriate control. However, did the authors also verify whether transient expression levels of rGFP-CAAX and β-arrestin2-RlucII (and thus the acceptor/donor ratio) remain comparable between empty vector- and double-dark Ggust-CASE-expressing cells? In my experience, enhanced bystander BRET (ebBRET) can be challenging, as ΔBRET values often increase linearly with the acceptor/donor ratio and rarely reach saturation, unlike NanoBRET, BRET1, or BRET2. Consequently, with rGFP and RlucII, ΔBRET values are highly sensitive to variations in acceptor/donor ratio. Please therefore report the acceptor/donor ratios at maximal agonist concentration for both empty vector and double-dark Ggust-CASE conditions for each receptor tested, to exclude artefactual interpretations.

We also thank the reviewer for this suggestion. We added the raw BRET values detected at the highest agonist concentrations for the two productive (A1R and D2R) and three unproductive (GPBAR, 5HT1AR, H2R) Ggust-couplers in the β-arrestin recruitment assay. Our data aligns well with the ∆BRET concentration response curves shown in **Fig. S14**, specifically, that raw BRET was only reduced for the unproductive G_{gust} couplers. This dataset is provided **Fig. S14H**.

Figure S8 (Comment 2): In panel B, the dose-response curve obtained with double-dark Ggust-CASE does not appear to be fitted with basal constrained to zero. In addition, an unpaired Student's t-test is not appropriate for comparing agonist-induced maximal efficacy. Please instead use an extra sum-of-squares F-test, as performed in Figure 3.

We constrained the bottom to zero and used the extra sum of squares F-test to check for statistical differences in the top ∆BRET plateau obtained with A1R, D2R, 5HT1AR, GPBAR, and H2R with and without gustducin overexpression. **Fig. S14** has been updated accordingly.

Reviewer #2 (Remarks to the Author):

Arce et al. report new BRET biosensors for gustducin activation and test whether non-taste GPCRs can engage gustducin. The key finding is that some GPCRs couple productively to gustducin, while others are unproductive gustducin-couplers.

We thank the reviewer for evaluating our manuscript.

Noteworthy results
The authors identify GPCRs co-expressed with GNAT3 in different tissues and test 24 selected in transfected HEK293A cells. In this system, they define two functional classes of GPCR-gustducin interactions: productive (H3R, A1R, BK2R, D2R, µOR, APLNR) and unproductive couplers (A2AR, H2R, 5HT1AR). I believe, a third group would be non-interactors.
One mechanistic claim is that unproductive GPCR-gustducin complexes suppress alternative G protein signaling pathways, but the supporting data are very limited.

We agree with the reviewer regarding the non-couplers and have added a short comment to the revised manuscript.
"Of the 26 receptors tested, 11 did not mediate BRET changes in Ggust-CASE and are therefore considered as non-G_{gust} *couplers."*

Significance and originality
The findings are likely significant, specifically for GPCR chemosensing and biased signaling. I believe the significance could be increased if not only a selection of receptors were validated, but also a broader panel.
The phenomenon itself is likely not Ggust-specific, but also relevant to other GPCR-G protein interactions. It would increase the study's significance if such a hypothesis were tested for selected receptors.

We thank the reviewer for this suggestion and expanded the set of GPCRs validating the concept of productive and unproductive coupling of non-tastant binding GPCRs to gustducin (see details below).

With respect to the generalizability of unproductive GPCR-G protein coupling, this concept has, in fact, been shown by two other labs earlier. The Lambert lab revealed unproductive V_2R-G_{12} complexes using cell-based assays (https://doi.org/10.1073/pnas.2003787117), and the lab of Christopher Garcia revealed the structures of unproductive US28-Gi complexes (DOI: 10.1126/sciadv.abl5442) – both studies are cited in our manuscript. Hence, the concept of unproductive GPCR-G protein complexes is in fact not G_{gust}-specific, but our study is the first one to show that these types of complexes can be formed with G_{gust} and what their implications are for alternative signaling pathways in cells.

As the study builds on previous results showing Ggust's involvement in pancreatic β cells, I would expect at least a few of the receptors reported in the cited studies to be included in the proper validation of the Ggust activation sensors, such as taste receptors, T1Rs, and T2Rs (reference 44) and/or GPR40, GPR41, GPR43, GPR119, GPR120, TGR5 (reference 45). This would enhance the study's significance by experimentally relating to previous findings.

We agree with the reviewer that the role of gustducin in pancreatic cells would be an intriguing scientific endeavor to be followed up. A few of the receptors suggested (in particular the GPRs), are, however, lacking tool ligands which could be used to acutely stimulate receptor activity in our pharmacological setup.

We hence turned our attention to the taste receptor Tas2R14 and the TGR5 (in our manuscript annotated GPBAR according to the IUPHAR nomenclature). We used agonist flufenamic acid (https://www.nature.com/articles/s41467-024-54157-6) to stimulate Tas2R14 and found that this receptor promotes gustducin heterotrimer dissociation as expected from a productive GPCR-G protein complex (**Fig. 1D**).
GPBAR, instead, induced an increase in BRET at Ggust-CASE upon stimulation with the agonist lithocholic acid (**Fig. 3B**), prompting us to examine whether it is an additional unproductive G_{gust}-coupler as observed for, e.g., the H_2R histamine receptor. Our subsequent experiments with GPBAR confirmed this hypothesis: GPBAR reduced $G\alpha_{gust}$-PDE6γ interaction (**Fig. 5**) and its signaling through the G_s-cAMP axis (**Fig. 7J, M**), as well as β-arrestin2 recruitment (**Fig. S14F**), were impaired by gustducin overexpression.

We are convinced that these findings enhance the study's significance, as suggested by the reviewer, and are grateful for this suggestion.

Do the data support the conclusions?
Mostly, the biosensor data support the notion that GPCRs can elicit distinct gustducin conformational/signaling outcomes, although the receptor selection in the validating assays should not be narrowed down. The PDE6γ assays should be performed for the full set of receptors shown in Figure 3A, as both productive and non-coupling should be demonstrated consistently for both systems.

To address the reviewer's concern, we performed $G\alpha_{gust}$-PDE6γ interaction experiments with 11 additional receptors, expanding the list of receptors that have been validated in both assays from 5 to 16 (**Fig. 5A**). The additional receptors included 2 more unproductive gustducin couplers (GPBAR and $5HT_4R$), 6 non-couplers ($5HT_{2B}R$, $5HT_6R$, D_5R, BK_1R, M_1R, $V_{1B}R$) and 3 productive couplers (MOR, APLNR and D_2R). In line with our previous findings, productive gustducin couplers induced a BRET increase, while unproductive couplers reduced BRET in the $G\alpha_{gust}$-PDE6γ interaction assay. For the 6 non-couplers, no concentration-dependent BRET change was observed upon agonist addition.

Further, in vivo physiological relevance remains to be tested, but is surely out of the scope of the present manuscript. However, this specifically means that additional evidence is needed to support broader claims about physiology, as the current evidence is largely from transiently transfected HEK293A cells rather than native intestinal, taste, pancreatic, or neuronal cells. The claim that these mechanisms regulate nutrient sensing or hormone secretion in vivo is plausible but not demonstrated.

We thank the reviewer for this comment and agree that it should be assessed in future studies, whether and to what extend these unproductive GPCR-gustducin complexes can be translated into in vivo settings.

Flaws or revisions needed

The main limitation is biological context: overexpression in HEK293A cells can alter receptor/G-protein stoichiometry, basal activity, coupling selectivity, and BRET geometry. This does not prohibit publication, but it requires careful wording and ideally validation in native or more physiologically relevant cells. According to the expression atlas, e.g., SW48 cells express high levels of GNAT3 and presumably also at least one of the chosen GPCRs, which could potentially serve as a preliminary model cell line.

We thank the reviewer for this comment and agree that receptor/G protein stoichiometry and cellular context can influence signaling behavior, and that heterologous systems do not fully recapitulate endogenous expression environments. However, our study aims to define whether gustducin can functionally couple to a set of non-taste GPCRs and to characterize this interaction using a controlled heterologous system. The primary conclusion is that these receptors are capable of engaging gustducin-mediated signaling under defined conditions. A key mechanistic finding of our study is the existence of two distinct classes of GPCR-gustducin complexes, corresponding to productive and unproductive states.

HEK293A cells are widely used for mechanistic GPCR–G protein coupling studies because they provide a controlled background, enabling direct assessment of coupling specificity and signaling outcomes. While we agree that more native systems can provide additional physiological context, such approaches introduce additional variables (other endogenous GPCRs, scaffolding proteins, competing G proteins, and variable expression levels) that can obscure direct interpretation of coupling specificity and mechanism, which is the primary focus of this work.

Regarding SW48 cells specifically, we agree that these or other GNAT3-expressing cell lines could serve as an interesting complementary model. However, a full characterization in such systems would require systematic optimization of receptor expression, endogenous signaling components, and assay calibration, and would therefore constitute a separate study. We consider this beyond the scope of the present manuscript, which focuses on defining coupling capability and mechanistic features in a reductionist system.

The interpretation of the increased BRET as evidence of unproductive coupling mechanisms is reasonable but not conclusively proven. Again, alternative approaches using endogenously GNAT3-expressing cells in combination with knockdown strategies and/or other assay readouts could strengthen the model.

We thank the reviewer for this comment and agree that alternative approaches such as studies in endogenously GNAT3-expressing cells combined with genetic perturbation and orthogonal readouts could further refine and extend the model. As the aim of our study is to establish and mechanistically characterize these coupling modes in a defined heterologous system, we consider more physiologically complex systems beyond the scope of the present manuscript.

Our interpretation of BRET changes is based on a consistent pattern observed across assays (e.g., an increase in BRET of Ggust-CASE correlates with a decreased BRET signal in the $G\alpha_{gust}$-PDEγ interaction assay and *vice versa*). Yet, as constant BRET users, we are aware of the fact that BRET signals are highly dependent on relative orientation changes of the energy partners and do therefore not always undoubtedly report on enhanced or reduced interaction of proteins. To address this concern particularly with respect to the interpretation of BRET changes reported by Ggust-CASE, we expanded the list of receptors tested in these orthogonal assays (see responses to other comments) and, additionally, performed an established $G\beta\gamma$ release assay in cells expressing only $G\alpha_{gust}$ but no other $G\alpha$ subtypes.

Importantly, this assay is considered as mostly independent from relative orientation changes of subunits in the G_{gust} heterotrimer because the interaction of masGRK3ct occurs only with $G\beta\gamma$ dimers liberated from $G\alpha$.

Using this assay, we found that productive G_{gust} couplers induced the release of $G\beta\gamma$ from $G\alpha_{gust}$, while the unproductive couplers H_2R and $A_{2A}R$ did not enhance BRET between $G\beta\gamma$ and the $G\beta\gamma$-scaffolding protein masGRK3ct labeled with Nluc. This complementary approach supports our interpretation of our previous results obtained with different assays and the data has been incorporated into the revised manuscript as Supplementary **Fig. S12**.

Further, the switching between several (histamine) receptors across different assay systems is unclear, which is more specifically outlined below.
In Figure 2B, it is not clear why H1R Gαq-Nluc, as “control,” is compared to H3R Gαgust-Nluc in Figure 2C. It would make more sense to show in Figure 2B Gi signaling of H3R as control, and in Figure 2D that Gαi-Nluc does not interact with PDE6γ-cpVenus upon H3R activation. As it is now, H1R is always flat, and the comparison between H1R and H3R here is confusing.

The H_1R and H_3R have been chosen due to their divergent coupling preferences. H_1R primarily couples to $G_{q/11}$ while H_3R couples $G_{i/o}$. $G\alpha_q$ does not share common effector proteins with members of the $G\alpha_{i/o}$ family, including G_{gust}, and we therefore implied that $G\alpha_q$ would serve as a valid negative control. This is confirmed by our data that shows no change in BRET between $G\alpha_q$-Nluc and PDE6γ-cpVenus.

We expected that using H_3R in combination with $G\alpha_{i2}$- or $G\alpha_{i3}$-Nluc would not serve as a valid negative control due to the high sequence homology of $G\alpha_{i1-3}$ proteins with $G\alpha_t$ and $G\alpha_{gust}$. In fact, we have assessed the interaction of PDE6γ with $G\alpha_{i2}$ and $G\alpha_{i3}$ and found a concentration dependent increase in BRET upon H_3R stimulation. To provide a clear negative control for comparison in our manuscript, we left the H_1R/ $G\alpha_q$ data in **Fig. 2B** but added the data obtained with $G\alpha_{i3}$ to the new supplementary **Fig. S4**.

It becomes clear from Figure S3 that H1R is a good Gq-coupler, but that H3R Gi2-coupling is as low as its coupling to Ggust. Why was Gi2 chosen and not Gi1 or Gi3? If the robustness of the system is to be established, the data for “standard” coupling should be more convincing, showing robust YFP-AC5 Gi and AC5-YFP-Gs interactions, thereby supporting the negative results for H3R. For H2R, Figure S3F does not support agonist-induced increases in BRET for Gs. If so, I would suggest adapting Figure S3F to look like S3E. It is also not clear why H2R was chosen here. It is only a secondary Gs-coupler. Similarly, TPα is primarily coupled to Gq and not G13.

The aim in **Fig. S3** was to identify an interaction partner of active $G\alpha_{gust}$ based on known effectors of other Gα subtypes. Our selection of GPCR/Gα pairs serving as positive controls was based on our own data and published literature (including meta-analyses as in Hauser et al., 2022 eLife) about GPCR-G protein couplings. Multiple studies have confirmed that the H_3R is a primary G_i/o coupling receptor, while the H_2R couples to G_s.

The very low response obtained with H_3R/G_{i2} in the $G\alpha_{i2}$-Nluc/AC5-YFP interaction assay is most likely a result of suboptimal BRET partner orientation or too long distances exceeding the range for sufficient energy transfer in the induced $G\alpha_{i2}$-AC5 complex. The same applies to the weak response obtained with H_2R/ $G\alpha_s$ and AC5. This is supported by our own data in **Fig. S8**, which shows substantial G_s activation by H_2R and by the data provided below, showing that the H_3R can equally activate Gi1-, Gi2- and Gi3-CASE.

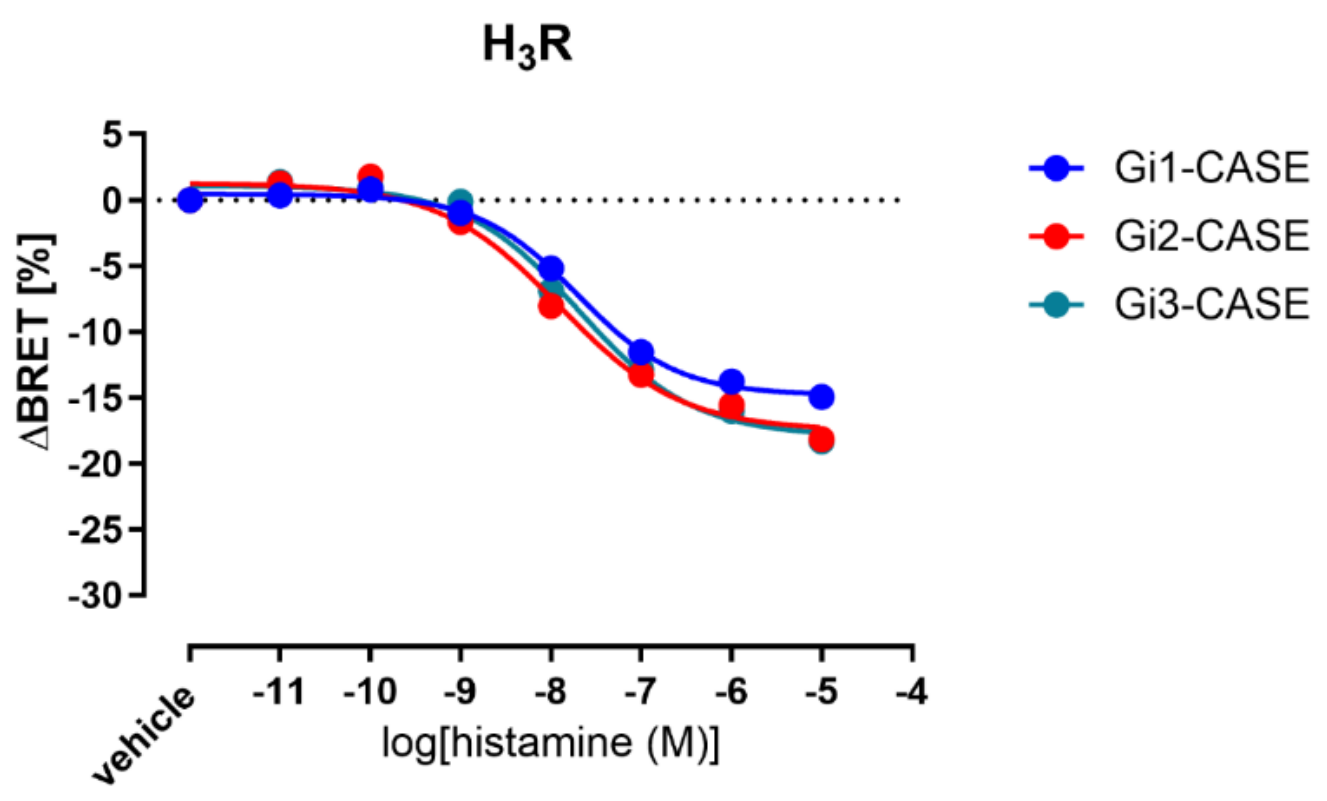

ΔBRET concentration-response curve of histamine in HEK-293A cells co-transfected with the H_3R and the indicated G-CASE sensors.

That H_2R primarily couples to and activates G_s is further supported by studies from the Bouvier (Avet et al. 2022 eLife; https://doi.org/10.7554/eLife.74101), Lambert (Okashah et al. 2019 Proc Nat Acad Sci U S A; https://doi.org/10.1073/pnas.1905993116) and Martemyanov lab (Masuho et al. 2023 Cell Rep; DOI: 10.1016/j.celrep.2023.113173). Notably, our protein-protein interaction data (requested by reviewer #1) obtained with the $G_{i/o}$(GTP)-coupling peptide KB1691-cpVenus and H3R/Gai2-Nluc confirms that H_3R can sufficiently activate G_{i2} and henceforth supports our hypothesis that BRET between $G\alpha_{i2}$ and $G\alpha_s$ with fluorescently labeled AC5 is sterically limited.

For the TPα, Inoue et al. 2019 Cell (DOI: 10.1016/j.celrep.2023.113173) and Masuho et al. 2023 Cell Rep report primary G12/13 coupling and we have used this receptor ourselves to validate G13-CASE sensors (Schihada et al. 2021 Sci Signal; DOI: 10.1126/scisignal.abf1653) while Okashah et al. 2019 Proc Nat Acad Sci U S A claims primary G_q coupling by the TPα. Importantly, the preference of the receptor for $G\alpha_{13}$ over $G\alpha_q$ is not relevant for our data in **Fig. S3** as long as the respective G protein can be activated by the chosen receptor because $G\alpha_{13}$ is directly labeled with the BRET donor – conferring specificity of the detected protein-protein interaction assay.
Regarding the reviewer's comment on the representation of **Fig. S3F** (now **Fig. S3G**), we agree with the suggestion and adapted its design to that of **Fig. S3E**.

According to the supplementary Table, it should be 201, not 202, GPCRs co-expressed with GNAT3.

Our supplementary file has two different sheets; one for the GPCRs expressed in the duodenum (202 GPCRs in total) and another sheet for the GPCRs expressed in the small intestine (201 GPCRs; all of which are also found in the duodenum). Our initial statement in the results section reads "In total, 202 GPCRs are co-expressed with $G\alpha_{gust}$ (GNAT3) in the duodenum and other parts of the small intestine (Supplementary Data 1)." and we revised it to "In total, 202 GPCRs are co-expressed with $G\alpha_{gust}$ (GNAT3) in the duodenum and *201 GPCRs are expressed in* the small intestine (Supplementary Data 1)." to specify our analysis.

Further, H3R is not co-expressed with GNAT3 in GIT/accessory tissues, brain, or adipose tissue, but was chosen as a productive coupler. I don't see the rationale for choosing H3R as a prototypical Gi/o-coupled GPCR to validate Ggust initially. It rather seems like a coincidence that the most productively coupling Gi-coupled GPCR was chosen.

We chose to use the H_3R to validate Ggust-CASE because this receptor for two practical reasons: (1) The H_3R is known for its preferential coupling to members of the $G_{i/o}$ protein family, (2) the H_3R is well expressed at the plasma membrane of HEK293 cells upon transient expression. We reasoned that the high sequence similarity of $G\alpha_{gust}$ to $G\alpha_{i1}$ and other members of the $G\alpha_{i/o}$ family could allow this receptor to couple to and activate G_{gust}, too.

During the course of our study, our single cell expression analysis also revealed that the H_3R is co-expressed with *GNAT3* at a single cell level. Specifically, 0.14% of brain excitatory neurons, 0.15% of brain inhibitory neurons and 1.34% of other brain neurons express both the H3R and GNAT3 (c.f., Supplementary Data 2), indicating that this receptor/G protein pair might in fact play a physiological role in the central nervous system. This information has been added to the manuscript.

I agree with the concentration-dependence of the deltaBRET signal, but think the time-dependence is a bit far-fetched, as it is neither interpreted nor does the signal change very much over time. Real kinetics analyses would need to be conducted in a much shorter time frame.

We agree with the reviewer's comment and removed the term "time-dependent" from the manuscript.

"Our selection included GPCRs with G protein coupling profiles ranging from very selective (e.g., V1BR, P2YR1) to very promiscuous (e.g., BK2R, H1R) and covered all four major Gα families." I do not find this reasoning for selecting the GPCRs very convincing. Although the cited publication, Hauser et al. 2022, shows that P2RY1 would very selectively couple to G15, several publications report Gq coupling for P2YR1, which is also inhibited by the Gq/11-selective inhibitor YM-254890. Similarly, in the cited publication, how is V1BR an example of a very selective G protein-coupling profile, whereas ADRA1D is, for example, equally selective?

We understand the reviewer's criticism. The classification of receptors as "selective" or "promiscuous" G protein couplers can be misleading as different studies may have come to different results. We corrected it in the revised manuscript and state that we selected a set of GPCRs whose collective coupling profiles cover all four major Gα families.

Wouldn't it, in turn, be more reasonable to argue that GPCRs that are indeed co-expressed with GNAT3 from co-expression analyses were chosen, specifically those that show the highest percentage of co-expression?

We agree with the reviewer that the degree of co-expression overlap could have served as a criterion for GPCR selection. However, this would have required a comprehensive pairwise single-cell co-expression analysis of *GNAT3* with all 202 GIT GPCRs, posing a substantial computational effort beyond the scope of the present study. Moreover, co-expression frequency alone does not necessarily predict functional receptor–gustducin coupling, as highly expressed GPCRs may show broad co-expression without being the most relevant signaling partners. Therefore, we used a combination of transcriptomic and biological considerations to prioritize candidates for pharmacological testing.

Regarding co-expression, I believe the reader would benefit from knowing the percentage of cells in which GNAT3 co-expresses with receptors in a selected cell type. A statement about a percentage range would surely be helpful, enabling the reader to know that it is not 20-100% of cells co-expressing both, but rather much lower numbers to put the findings in context. This would not affect the significance of the findings but would make it clearer for follow-up studies when designing their experiments.

We agree with the reviewer's comment and added the following sentence to the manuscript:

"*Maximum expression overlap with GNAT3 was detected for CHRM3 and P2YR1 in stomach tuft cells (17%), while the minimum degree of co-expression was observed for AVPR1B, BDKRB2 and HTR1A (0.02 – 0.03% in pancreatic islet cells, ependymal glial cells, and brain excitatory neurons, respectively;* ***Supplementary Data 2****).*"

Additionally, a column stating the degree of co-expression overlap was added to supplementary data 2.

Similar to H2R, I would suggest including GCASE controls for the primary and secondary signal transduction pathways for the other 14 receptors tested to ensure they function in the assay system.

We thank the reviewer for this comment. We tested all GPCRs included in our study for activating their primary and secondary G proteins using the appropriate G-CASE sensors (note that for the GPBAR only coupling to G_s has been reported; therefore, no secondary G-protein was included). Our results show that all 26 non-tastant binding receptors included in our manuscript mediate G-CASE BRET changes upon stimulation with agonists (**Fig. S6**). Of note, BK_1R and $5HT_{2B}R$ induced increases in BRET with sensors of their primary G proteins, indicating receptor-mediated rearrangement of the G protein heterotrimer, as reported earlier (https://doi.org/10.1073/pnas.2536719100; https://doi.org/10.1074/jbc.M414630200; https://doi.org/10.1038/nsmb1134).

As G-CASE sensors have been reported before, the design itself is not new.
However, two additional GCASE sensors are mentioned in the text, G11-CASE and Go2-CASE.
In Figure S5, H2R shows no response with G11-CASE and a very low response with Go2-CASE.
Figure S7 shows a histamine-dependent G11-CASE activation for H1R.
For G11, several of the selected receptors make good candidates to properly validate the G11 CASE sensor, as they are in Hauser et al 2022 depicted as G11-couplers, such as A1R, A2AR,

P2YR11, M1R, M2R, M3R, HT2R (which one a, b or c?), V1B, BK1, BK2.
The same is true for GoB and A1R, M1R, M3R, M4R, HT1R (which one a, b, d, e or f?) HT2R (which one a, b or c?), V1B, BK1, BK2, the other Histamine receptor, D2R, D5, CB1, APLNR, etc.
I suggest including a proper validation of these two sensors by showing data for more than two selected GPCRs. The rationale for choosing these two is not clear.

We thank the reviewer for this suggestion and performed additional experiments. We validated the sensor Go2-CASE in agonist concentration-response experiments with the receptors MOR, D_2R and M_4R. The new G11-CASE sensor was validated using the $\alpha_{1A}AR$, M_5R and BK_2R as model receptors. Our new data, which has been added to Supplementary **Fig. S9C** and **Fig. S10C**, shows concentration-dependent decrease in BRET and confirm the functionality of both G-CASE sensors.

In Figure 3B, some receptor responses are difficult to distinguish, such as MT2R and A1R. I also think the figure would benefit from rearrangement, not grouping by primary G protein-coupling, but rather, for example, by ligand type and receptor family (all 4 HTR in one graph, P2Y with adenosine receptors, histamine receptors with dopamine receptors).

We agree with the reviewer that the initial arrangement in **Fig. 3** was not ideal. We decided to re-group the datasets in **Fig. 3B** according to the response induced by the receptors: no change in BRET (= non-couplers; group A) – decrease in BRET (= productive couplers; group B) – increase in BRET (= unproductive couplers; group C).

Are there other examples in which receptor activation actually causes an increase in GCASE-BRET responses? It seems likely that this is not a Ggust-specific phenomenon.

Yes, this has been observed before in the study of Okashah et al., 2020, PNAS; https://doi.org/10.1073/pnas.2003787117). This study describes unproductive complex formation of the V2R receptor with G12 and supplementary figure S5B shows BRET increases with sensors equivalent to our G-CASE probes. We've added a short statement regarding these equivalent results to our manuscript.

The low potency of GTPγS should not be stated as pEC50 but rather as the actual value to help the reader with the direct comparison (~30-60 fold lower potency).

We agree with this comment and changed the pEC_{50} value to EC_{50}.

Although the GTPγS-induced decrease in BRET may indicate that Ggust is partially active, the GTPγS concentrations required to observe this decrease, as stated by the authors, were very high. Again, here I would speculate that this is not a Ggust CASE-specific phenomenon.

The GTPγS concentrations required to elicit Ggust-CASE activation are in fact in the μM range with an EC_{50} value of around 3 μM. However, intracellular GTP concentrations are around 100 – 300 μM, i.e., 30 – 100-fold over the EC_{50}. This indicates that G_{gust} elicits constitutive activity at cellular nucleotide concentrations in intact cells.

We also agree with the reviewer that this apparently low potency of GTPγS might not be a Ggust-CASE specific phenomenon and performed similar experiments with other G-CASE probes. Our results confirm that also other G-CASE sensors have EC_{50} values of GTPγS in the single- to even double-digit micromolar range. More details can be found in our reply to the comment below.

Furthermore, HEK293 cells endogenously express several GPCRs that are relevant to the observed results. I think that an analogous analysis with other GCASE sensors that show or do not show basal activity using these GTP assays would be valuable, potentially also to compare the different sensors and draw broader conclusions.

We agree with the reviewer's idea that this low apparent potency of GTPγS is not a Ggust-specific phenomenon and conducted similar experiments in cells expressing other G-CASE sensors representing each of the four major Gα families (G_{i2} for $G_{i/o}$; G_{13} for $G_{12/13}$; G_q for $G_{q/11}$ and $G_{s(long)}$ for $G_{s/olf}$). The concentration-response curves obtained with GTPγS reveal similarly low potency with EC_{50} values, ranging from 2 µM at Gi2-CASE to 15 µM at Gs(long)-CASE. This new data has been added as Supplementary **Fig. S13**.

In Figure 3, BK2R is a productive Ggust-coupler like A1R and D2R and H3R (Figure 1C), but in Figure 6B, presumably, "biphasic behavior". Because the concentration of each agonist is unspecified and a single concentration is used, the data do not adequately support the point.

→ We understand that our wording can be misleading as "biphasic" is often associated with concentration-response curves which show two phases of response. In Figure 3, we were referring to the temporal biphasicity of the response but to avoid misconception of the readers, we edited our wording accordingly:

Before: GTPγS dissociated the receptor complexes with G_{gust} for H_3R and A_1R, while BK_2R exhibited a biphasic behavior, indicating initial complex rearrangement followed by dissociation (**Fig. 6B**).

After: GTPγS dissociated the receptor complexes with G_{gust} for H_3R and A_1R, while BK_2R exhibited a two-phase temporal response, indicating initial complex rearrangement followed by dissociation (**Fig. 6B**).

The authors state that GPCR-Ggust impacts other G protein-GPCR couplings and use a double dark Ggust-CASE.
What is not clear here is why A1R was chosen to analyze for Gq, while it primarily couples to Gi, and why H2R, a primarily Gq-coupled receptor, was chosen to test Gs (long). A1R's secondary signaling mechanisms are Gs- and Gq-coupling. However, to make the point that Ggust is the alternative pathway to the respective secondary transduction mechanisms, similar experiments should be performed testing the GCASE sensor for the primary signal transduction mechanism.

The reviewer is right and the A_1R primarily couples to $G_{i/o}$ and not G_q. We've added experiments with A_1R and Gi2-CASE with and without G_{gust} overexpression. This new dataset

confirms what we already observed with G_q. G_{gust} overexpression does not affect the activation of the primary or secondary transduction partner, G_{i2} or G_q, by the A_1R (**Fig. 7B**).

The H_2R is, however, a primary G_s coupler (not G_q) as supported by studies from the labs of Michel Bouvier (Avet et al. 2022 eLife; https://doi.org/10.7554/eLife.74101), Nevin Lambert (Okashah et al. 2019 Proc Nat Acad Sci U S A; https://doi.org/10.1073/pnas.1905993116) and Kirill Martemyanov (Masuho et al. 2023 Cell Rep; DOI: 10.1016/j.celrep.2023.113173), and the meta-analysis from Hauser et al., 2022 eLife; https://doi.org/10.7554/eLife.74107). Therefore, our initial dataset for H_2R-mediated G_s activation in the presence of overexpressed G_{gust} already represents the primary transduction pathway. To additionally assess the impact of G_{gust} overexpression on the activation of secondary G_q coupling by H_2R, we also performed these experiments and found a substantial reduction in G_q activity due to unproductive H_2R-Ggust coupling (**Fig. 7G**), in line with our previous experiments which showed that unproductive Ggust coupling impairs the activation of alternative signaling pathways.

Although Figure 7C effectively illustrates the broader validity of the results, the experimental evidence is insufficient at present. BK2R, D2R, D2R, and APLNR as productive couplers could be included as additional examples supporting the conclusion. Similarly, for unproductive couplers A2AR, 5HT1AR, 5HT4R, and M4R.

We thank the reviewer for this suggestion. We conducted additional experiments to demonstrate the broader validity of our results. As can be seen from the new datasets implemented into **Fig. 7**, the activation of primary G proteins remains unaffected by G_{gust} for productive Ggust-couplers (**Fig. 7A-E**), whereas unproductive G_{gust} couplers are substantially impaired in activating alternative G proteins (**Fig. 7F-J**) and promote changes in second messenger levels in cells (**Fig. 7K-M**).

We believe these additional datasets provide sufficient experimental evidence for the validity of our results sketched in **Fig. 8** (formerly **Fig. 7C**).

The authors controlled for H2R cell surface expression and observed no significant differences, but saw reduced arrestin translocation to the plasma membrane with Ggust-CASE double dark. Figure S8D suggests that 2 of the 6 experiments showed the opposite direction, and that if these were excluded, H2R cell surface expression would be decreased. It would be helpful if there were lines between the matching experiments, or if another, more robust assay system was used to support this conclusion.

We added lines to **Fig. S14G** (formerly **Fig. S8D**) to connect the paired data points. These pairings show that the 2-times 2 datapoints with opposite direction spotted by the reviewer originate from different biological replicates, confirming that co-expression of dd Ggust-CASE did not alter the surface expression level of the H_2R.

HEK293A cells endogenously express GPCRs that may be activated by the chosen ligands, such as 5-HT receptor subtypes. Can interferences with the assay readout be excluded?

The reviewer raises a valid point. We used two different readouts, Ggust-CASE activation/rearrangement (**Fig. S11**) and β-arrestin2 translocation to the membrane (**Fig. S14D, E**), in cells without GPCR overexpression and stimulated with the endogenous agonists of serotonin and histamine receptors. Our data shows no interference by endogenous receptors in either of the two readouts.

Methodology

The methodology is generally sound for live-cell GPCR signaling pharmacology with NanoLuc/cpVenus BRET sensors. The sensors are likely to be valuable to the broader community, although further validation within the present manuscript is required. The inclusion of dark/double-dark variants strengthens the results, as does the GTPγS/GDPβS nucleotide manipulation.

However, there is a need for broader validation of the new sensors G11 and GoA, specifically to compare them to the Ggust-CASE signals. Similarly, showing that the primary and, if applicable, secondary G protein signal transduction pathways can be reliably detected with the respective (for each GPCR) relevant GCASE sensor is mandatory to ensure the system itself is robust. As it stands, the comparison with other G-protein biosensors is insufficient. The work meets expected standards for a mechanistic cell-signaling study, but not yet for strong physiological claims.

We would like to thank the reviewer again for the feedback provided. As mentioned above, we have carried out a broader validation of G11- and Go2-CASE and examined primary and secondary G protein activation by the receptors used in our study.

Reproducibility

The methods provide details on reagents, constructs, cell handling, permeabilization, nucleotide treatments, BRET assays. However, it is not clear which datapoint(s) was/were used from the time-curve(s), such as 2C, to generate curves, such as shown in 2D or 3B. Since deltaBRET is still reported as a percentage, I assume the AUC was not interpolated. The statement in the method section, "time points with saturating response of all concentrations tested were selected," is insufficiently clear and suggests that the choice differed between receptors.

To generate concentration-response curves, the time courses of ligand-induced BRET changes were manually inspected, and the time points at which the response obtained with the highest ligand concentrations was at its maximum were selected. To account for the different ligand-binding kinetics underlying the interaction between agonists and receptors, as well as the different coupling kinetics between receptors and G proteins, a universal time point could not be taken across all receptors tested.

We've added more information to the data analysis section of our manuscript to provide the readers with more background information regarding those concentration-response curves.

Reviewer #3 (Remarks to the Author):

In this manuscript Jaime Arce et al. develop biosensors for the detection of gustducin coupling and activation and describe the ability of this G protein to interact with non-taste GPCRs to form what they call productive or unproductive complexes. By building on the TRUPATH and G-CASE toolbox, the authors have generated Ggust-CASE, a biosensor that detects the dissociation of the Ggust heterotrimer. Additionally, they have developed a BRET approach that monitors the proximity between Galphagust and PDE6gamma, as an orthogonal measure of G protein activation. These two sensors are potentially of interest to the wide GPCR community. In the second, more conceptual part of this manuscript, the authors describe a mechanism whereby coupling of some GPCRs to Ggust can result in two outcomes: productive coupling and G protein heterotrimer dissociation or unproductive coupling and sequestration of GPCRs from coupling to other G proteins.
Overall, this is an interesting study with potential technical and conceptual advances. However, in the present form, the manuscript lacks data to support the generalisability of these advances, as claimed by the authors. Thus, with the acknowledgement that this work presents a potentially useful tool for GPCR research, there are a few considerations that limit our enthusiasm for this manuscript in the current form.

We thank both reviewers for critically assessing the quality of our work and the feedback provided.

General comments:
1. While the focus of the study is to describe the activation of and interactions between Ggust and non-gustatory receptors, it is surprising that the authors did not include Ggust-CASE experiments with any tastant-activated GPCRs. These experiments would confirm that the newly created sensor is suited to also pick up signalling responses of receptors that are primarily coupled to Ggust.

We thank the reviewers for this comment. For the revision of our manuscript, we obtained a plasmid for the expression of the human taste receptor Tas2R14 in HEK293 cells. The 3D structure of this receptor has previously been solved in complex with the agonist flufenamic acid and a chimeric mini G protein of Ggust/s and we assessed whether Tas2R14 stimulation with flufenamic acid would induce a substantial BRET change at our Ggust-CASE biosensor.

Our new experiments showed that flufenamic acid induced a concentration-dependent decrease in Ggust-CASE BRET in cells transfected with the Tas2R14 (**Fig. 1D**), confirming the validity of our biosensor and indicating that this taste receptor forms productive complexes with gustducin.

2. The data displayed in Figure 3B appears to be one of the main findings of this study. The panel of used receptors is nicely justified by the tissue and single-cell expression data analysis, however, the authors could find a way to better showcase the behaviours of these different GPCRs, in addition to concentration-response curves. For example, a clustering heatmap of those responses would allow for the evaluation of all tested GPCRs and aid in the classification of receptors based on these.

We agree with the reviewers that the data presentation was not ideal and re-organized **Fig. 3B**. In the new version, the receptors are clustered into three different groups, based on the response they induced in Ggust-CASE: group A - non-couplers (no significant BRET change); group B - productive couplers (BRET decrease); group C - unproductive couplers (BRET increase).

3. In line with the previous comment, they authors should show that for those GPCRs with productive coupling, this coupling sensitive to antagonist treatment.

We performed the suggested experiment for four representative, productive G_{gust} couplers. We used

- 10 µM clobenpropit to block 1 µM histamine-induced Ggust activation by the H_3R,
- 10 µM naloxone to block 100 nM DAMGO-induced Ggust activation by the MOR,
- 10 µM risperidone to block 100 nM dopamine-induced Ggust activation by the D_2R,
- and 10 µM icatibant to block 10 nM bradykinin-induced Ggust activation by the BK_2R.

Our results, shown in Supplementary **Fig. S7** show that all antagonists blocked agonist-induced G_{gust} activation by these productive G_{gust} couplers.

4. One of the main conclusions of the study relies on the measurements in Figure 7 and S8, which are only performed at two receptors: the A1R and H2R. While the G protein activation data in Figure 7A and B is convincing, the data in S8 does not show robust effects. In addition, to support the proposed mechanism, these (G protein activation and arrestin bystander recruitment experiments) should also be performed for the entire set of receptors featured in Figure 3, or at least a more substantial panel of receptors. This will allow for a comprehensive assessment of the proposed sequestration mechanism.

Based on the reviewers' suggestion, we performed additional experiments with 7 GPCR-G protein pairs and 4 GPCR-arrestin pairs. The outcome of these experiments aligns with our previous observation:

The activation of alternative G proteins by productive gustducin couplers H_3R, A_1R (now with Gi2-CASE), APLNR and D_2R was <u>in</u>sensitive to gustducin overexpression (Fig. 7A-E). In contrast, alternative G protein activation by <u>un</u>productive gustducin couplers $A_{2A}R$, GPBAR, $5HT_{1A}R$, H_2R (now measured with the secondary G protein Gq) was impaired in the presence of overexpressed G_{gust} (**Fig. 7F-J**). These findings are further corroborated by the fact that impaired activation of Gs due to unproductive complexes with Ggust translates into reduced cAMP production downstream of activated H2R, A2AR and GPBAR (**Fig. 7K-M**; additional control experiment showing that Ggust overexpression without unproductive coupling does not affect cAMP in **Fig. 7N**).

Also, with respect to β-arrestin2 recruitment, the productive G_{gust} coupler D_2R was unaffected by G_{gust} overexpression (**Fig. S15B**), while gustducin limited recruitment to the unproductive couplers $5HT_{1A}R$ and GPBAR (**Fig. S15E, F**). We also tested the $A_{2A}R$ and H_3R in

this arrestin recruitment assay, but arrestin translocation could not be detected even in the absence of gustducin overexpression.

5. Ggust belongs to the Gi/o/z family, and its description of coupling outside the taste receptor family is interesting. However, there are a few characterisation steps of the sensor and descriptions that are missing:
a. Ggust transduces signal via AC inhibition. Does the “productive” coupling of Ggust by non-taste GPCRs decrease cAMP levels?

The questions raised by the reviewers are interesting and we performed a set of experiments to understand the signaling pathway mediated by gustducin.

We assessed whether gustducin activation induced by the non-taste, productive receptor H_3R promotes a decrease in cAMP by modulating adenylyl cyclase activity. To exclude the effect of other AC-modulating G proteins (G_s and $G_{i/o}$), we used HEK293 cells devoid of any endogenous $G\alpha$ proteins and transfected them with H_3R, the cAMP GloSensor and $G\alpha_{gust}$ wildtype. We pre-stimulated with 100 µM forskolin to raise the basal level of cAMP in the cell and then activated the H_3R with histamine.

Our results (**Fig. S3J**) show that H_3R-mediated G_{gust} activation does not induce changes in cAMP. Possible explanations include the hypotheses (a) that activation of Ggust by non-taste receptor is not efficient enough to promote Gagust-mediated AC inhibition and (b) that the adenylyl cyclase pool expressed in these HEK-cells might not comprise $G\alpha_{gust}$-sensitive enzymes.

b. Is the sensor and the potential cAMP response sensitive to PTx? It seems a missed opportunity to use PTx to provide further insight into the distinction of productive/unproductive coupling

This is an interesting suggestion raised by the reviewer. We performed Ggust-CASE experiments with the H_2R (representative for unproductive couplers) and the A_1R (representative for productive couplers) with and without PTX treatment. As expected, A1R-induced BRET changes in Ggust-CASE were blocked by PTX (**Fig. 6D**), compatible with the conventional mode of GPCR-G protein coupling that involved the engagement of the C-terminal α5 helix of $G\alpha$ with the open receptor core. Surprisingly, H_2R-mediated increases in BRET changes of Ggust-CASE are **not** affected by PTX treatment (**Fig. 6E**). This data suggests that the C-terminal α5 helix of $G\alpha_{gust}$ is not involved in the unproductive H_2R-Ggust complex (**Fig. 6F**).

Interestingly, the concept of α5-independent unproductive GPCR-Ggust complexes is supported by cryo-EM structures of unproductive virus-encoded GPCRs complexed with Gi1 (Tsutsumi et al., 2022, Sci Advances; OI: 10.1126/sciadv.abl5442). These structures show little to no engagement of the G protein’s α5 helix with the core of US27 – compatible with the PTX-insensitive coupling mechanism suggested by our data.

6. In line with the comment above, sequestration and "unproductivity" would be more relevant if tested for downstream, second messenger assays.

We agree to the reviewer's comment and performed cAMP GloSensor experiments to confirm the sequestration of unproductive receptors by G_{gust}.

For all our cAMP measurements, we used HEK293 cells devoid of endogenous Gα proteins. We transfected these cells with the cAMP GloSensor, an unproductive G_{gust} coupler which would primarily couple to G_s (GPBAR, H_2R, $A_{2A}R$), wildtype Gαs, and double dark Ggust-CASE or empty vector. These experiments showed that the co-expression of Ggust reduced receptor-mediated constitutive and agonist-induced production of cAMP for all three receptors tested (**Fig. 7K-M**). In a control experiment, we confirmed that this effect is not due to constitutive inhibition of cAMP production by G_{gust} overexpression. Ggust overexpression had no effect on basal or FSK-induced cAMP levels in these cells (**Fig. 7N**).

Overall, these results confirm that G_{gust}-mediated sequestration of GPCRs impairs the activation of alternative pathways in cells.

7. Finally, there is no consideration of the GDP/GTP exchange rate or GTPase activity of gustducin? Could differences among other G protein isoforms explain some of the data? While, if different this could be experimentally validated, the authors should at least discuss this possibility.

We agree with the reviewer criticism that the GTPγS data obtained with Ggust-CASE can be further examined. Of note, the assay barely reflects on GTPase activity because the GTP derivative used, GTPγS, cannot be cleaved by Gα. To better understand whether the GTPγS-induced effects at Ggust-CASE are similar at other G proteins, we conducted analogous experiments in cells expressing other G-CASE sensors representing each of the four major Ga families (G_{i2} for $G_{i/o}$; G_{13} for $G_{12/13}$; G_q for $G_{q/11}$ and $G_{s(long)}$ for $G_{s/olf}$). The concentration-response curves obtained with GTPγS reveal similarly low potency with EC_{50} values, ranging from 2 µM at Gi2-CASE to 15 µM at Gs-CASE (**Fig. S13**).

Specific/methodological comments:
8. The kinetic resolution of data of Figure 1C and similar could use some kinetic curve fitting.

The temporal resolution in these time course datasets is usually too low to allow for meaningful interpretation of rate constants. If the kinetic aspect of G_{gust} activation and signal transduction would have been a focus of our study, we would have performed these experiments in well (not plate) mode and combined automated injectors for ligand application. The kinetics aspects of these G protein responses are, however, confounded by distinct kinetics of GPCR agonist-receptor binding and go beyond the scope of our study.

9. Raw baseline data is not presented in any figure, this is particularly important for GDP/GTPgS experiments (but also in the different GPCR coupling description) and their interpretation. The authors should provide and discuss this.

The reviewer touches upon an important point. By default, we always check the raw baseline BRET in all our projects before transforming them into vehicle-corrected ΔBRET values. If these raw baseline values provide any indication of differences between samples prior to ligand-induced stimulation of the system, we add these raw values to our manuscript.

We've added this background information to the methods section of our manuscript.

Throughout this project, no such differences have occurred to us with the exception of the baseline cAMP GloSensor luminescence values induced by unproductive GPCRs with and without G_{gust} overexpression (Fig. 7K-M) where we provide the pre-normalized data only.

Minor comments:

Abstract:

• "…characterization of Ggust activity by non-taste GPCRs." Should be: "…characterization of Ggust activity induced by non-taste GPCRs."

Thank you. We added "induced" as suggested.

• Throughout the manuscript, the authors use a mixture of hyphens and EN-dashes to describe protein–protein interactions e.g. "…unproductive GPCR-G protein complexes,…" Here, the EN-dash should be used instead of a hyphen: "…unproductive GPCR–G protein complexes,…" and this should be consistent throughout the manuscript.

Thank you for the comment. We checked our manuscript to make sure that EN-dashes are used for all protein–protein interactions.

Methods:

• Clarify how deltaBRET was calculated (from AUC vs at a particular time point).

We've added more details to our methods section to clarify how delta BRET for concentration-response curves was determined.

Introduction:

• "Here, Gαgust couples taste receptors to glucagon-like peptide 1 (GLP-1) secretion, ghrelin release and expression of sodium-dependent glucose transporter isoform 1 (SGLT1)[11–13]." We suggest to change "couples" to "links" to avoid confusion about G protein-coupling.

Thank you. We replaced "couples" by "links" as suggested.

• GPCR-specific conformational responses in the Ggust heterotrimer: Instead of the possessive "Ggust's", we recommend rephrasing to "the primary physiological function of Ggust…" to improve readability.

Thank you. We employed the suggested change.

Discussion:

• "…complements recent reports of unproductive GPCR–G protein complexes and raises intriguing question regarding the physiological consequences…" should be "…raising intriguing questions..."

Thank you. We employed the suggested change.

Reviewer #4 (Remarks to the Author):

I co-reviewed this manuscript with one of the reviewers who provided the listed reports. This is part of the Nature Communications initiative to facilitate training in peer review and to provide appropriate recognition for Early Career Researchers who co-review manuscripts.

We thank also this reviewer for evaluating the quality and significance of our study.

Point-by-point response to reviewer comments on

"Functional diversity of GPCR-Gustducin complexes controls signaling output and suppresses alternative pathways"

Reviewer #1 (Remarks to the Author):

The authors have addressed my previous concerns carefully and constructively, and the revised manuscript has been substantially improved. I appreciate the authors' thorough responses to my previous comments.

We thank the reviewer for their time and effort in evaluating the revised manuscript. We are glad to hear that their previous concerns have been addressed.

I have only a few minor concerns remaining:

• Line 183: The authors state that they confirmed that all 26 receptors were able to activate sensors of the primary and/or secondary G proteins upon co-expression and agonist stimulation, referring to Figure S6. I am not sure that "activation" is the appropriate term here, as the ΔBRET values observed for BK1R and 5-HT2BR with their primary G protein, Gq, are positive rather than negative. With the G-CASE sensor, a positive ΔBRET would appear to indicate non-productive coupling rather than G protein activation. The wording should therefore be revised to distinguish receptor-G protein engagement from productive G protein activation.

We thank the reviewer for this comment and completely agree. Accordingly, we changed our wording from "activate" to "engage".

• Figure 3B: Ggust is a member of the Gi/o family. It is therefore expected that receptors in cluster B, which are productive Ggust couplers, predominantly activate members of the Gi/o family. However, BK2R appears to represent an interesting exception. According to Figure S6, BK2R primarily couples to Gq and secondarily to G13, yet it is classified as a productive Ggust coupler. Do the authors have an explanation for why BK2R does not appear to activate the canonical Gi/o proteins tested, while nevertheless showing productive coupling to Ggust? At minimum, this apparent exception should be acknowledged in the text accompanying Figure 3B.

The reviewer's observation is in fact very interesting. We tested BK2R coupling to Gq and G13 in Figure S6 because these G proteins appear as primary and secondary partners in the gpcrdb.org coupling database. However, according to Hauser et al., eLife 2022 (https://elifesciences.org/articles/74107), Avet et al., eLife 2022 (https://elifesciences.org/articles/74101#s2) and the meta analysis of the gpcrdb.org database, this receptor is also activating multiple members of the Gi/o family. We could

confirm BK2R-mediated activation of Gi/o family members with our own G-CASE probes in a separate project and the data is pasted below.

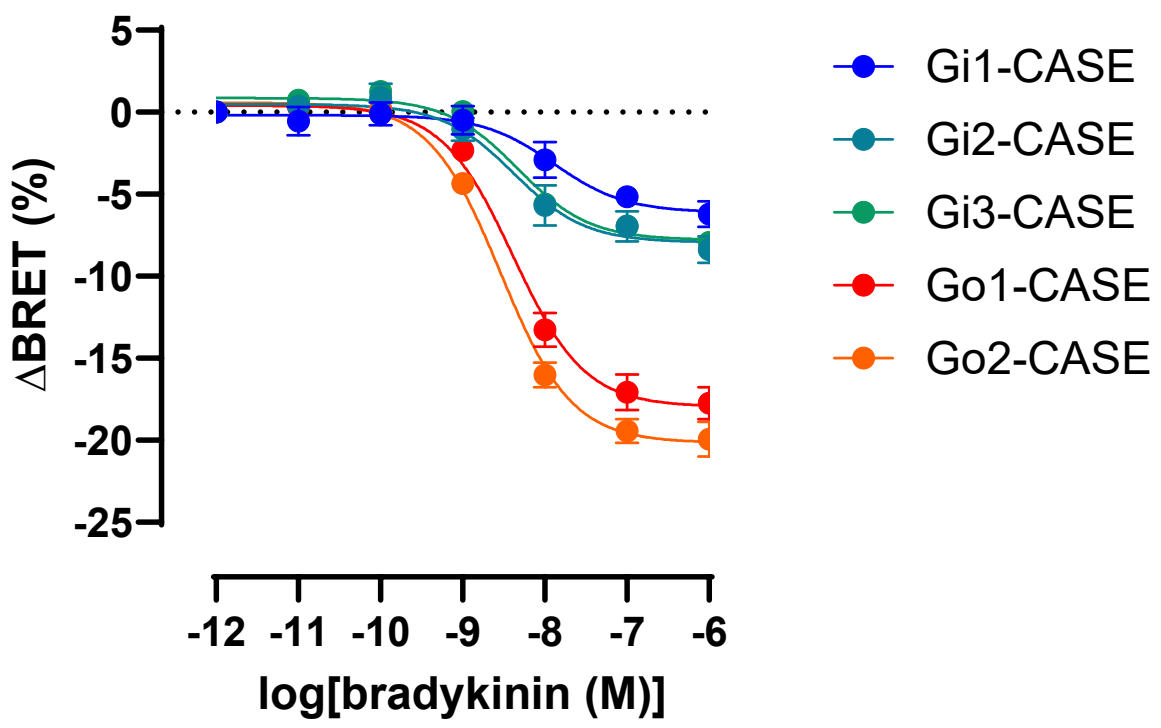

The capacity of the BK2R to activate G_i- and G_o proteins matches well with the other receptors that form productive complexes with Ggust. To address this point in our manuscript, we added a short statement regarding the promiscuous coupling behavior of the BK2R.

Of note, all receptors that induced a decrease in Ggust-CASE BRET have previously been shown to activate $G_{i/o}$ family G proteins, including BK_2R, which exhibits promiscuous G protein coupling[33].

• Line 190: The authors state that GPCR activation induced not only decreases in Ggust-CASE BRET, which were sensitive to pre-incubation with GPCR agonists, but also positive BRET changes. I believe the authors mean antagonists rather than agonists here. Please correct.

The reviewer is correct! Thank you very much for spotting this error. We corrected it accordingly.

• Figure S14: The newly added panel H of Figure S14 is not described in the Results section. Please ensure that this panel and its main finding are appropriately introduced and discussed in the Results.

We agree and added the following statement to the text referring to Fig. S14: *"Importantly, the surface expression levels of the H_2R were similar in experiments with and without double dark Ggust-CASE co-expression (**Fig. S14G**) and the raw BRET values confirmed the effects of G_{gust} overexpression independent of altering basal BRET values (**Fig. S14H**)."*

• Figure S15: Similarly, I could not identify a corresponding description of Figure S15 in the Results section. Since this supplementary figure is subsequently referred to in the Discussion (line 414), the relevant results should first be presented in the Results section.

We thank the reviewer for this comment and added the following statement to the results section:

Mechanistically, the observation that receptors, such as the H2R, which form unproductive complexes with Ggust, conventionally activate other Gi/o family members despite the high

sequence similarity of the Gα subunits in their C-terminal region (Fig. S15), suggests that structural determinants outside the Gα C-terminus contribute to the ability of receptors to promote productive nucleotide exchange of Ggust.

Reviewer #2 (Remarks to the Author):

The authors substantially expanded the study's experimental scope and addressed my previous concerns. They extended the set of non-tastant GPCRs used to assess productive and unproductive coupling and added further examples of unproductive GPCR-G protein complexes. They also included Tas2R14 and TGR5 in their evaluation, with the latter providing a convincing example of unproductive Ggust coupling. Together, these additions broaden the relevance of the findings and strengthen the overall significance of the study. I agree with the authors that it is not necessary to examine all receptors expressed in pancreatic β cells, although I disagree that all GPRs lack suitable tool ligands.
The authors also considerably strengthened the experimental validation of their approach. They expanded the PDE6γ validation from 5 to 16 receptors and added an orthogonal Gβγ-release assay. In addition, G11-CASE and Go2-CASE were validated with further receptors, and primary and secondary G-protein signaling was examined across the receptor panel. Further, Figure 7 was expanded, and additional controls were included to address potential interference from endogenous receptors.
In summary, I believe these complementary data strengthen the manuscript's conclusions. They further support grouping GPCRs into productive and unproductive couplers. The authors have addressed my concerns, and I support publication of the revised manuscript. I look forward to future studies in cells endogenously expressing GNAT3 and the relevant GPCRs to establish the physiological relevance of these different coupling behaviors.

We thank the reviewer for their time and effort in evaluating the revised manuscript. We are glad to hear that their previous concerns have been addressed sufficiently.

Minor comments
Figure S6: Was the two-way ANOVA followed by Fisher's LSD multiple-comparisons test performed against a normalized value of zero without associated error? If so, I am not sure that this comparison is appropriate. I also do not think it is necessary to plot a vehicle (VH) value of zero for each receptor. More generally, I do not see a clear need for statistical testing in this analysis.

We thank the reviewer for bringing this to our attention. Indeed, the analysis was performed using a two-way ANOVA followed by Fisher's LSD multiple-comparisons test against a normalized value of zero without associated error. For the revised manuscript, we've instead used the one-sample t test against the hypothetical value of 0. The legend of Figure S6 has been revised accordingly and the vehicle values have been removed from the figure, as suggested.

“Flufenamic acid” appears as “lufenamic acid” at one point before Figure 1.
The Methods section refers to KB1694, whereas the Results section refers to KB1691. Please verify the correct identifier and use it consistently.

Thank you for spotting this typo. We corrected it in the revised manuscript file.

Reviewer #3 (Remarks to the Author):

The authors have addressed our concerns appropriatedly. Just note a couple of typos:
- Page 3 end of the first paragraph: name of TasR ligand is misspelled.
- Page 6 :"...which were sensitive to pre-incubation with GPCR agonists (Fig. S7),..." presumably the authors mean antagonists?

We thank the reviewer for their time and effort in evaluating the revised manuscript. We are glad to hear that their previous concerns have been addressed sufficiently. We corrected the typos spotted by the reviewer.

Reviewer #4 (Remarks to the Author):

I co-reviewed this manuscript with one of the reviewers who provided the listed reports. This is part of the Nature Communications initiative to facilitate training in peer review and to provide appropriate recognition for Early Career Researchers who co-review manuscripts.

We also thank this reviewer for their time and effort in evaluating the revised manuscript.
